# Supplementary material for: Methylome profiling of SetDB1-deficient ESCs reveals coordinated epigenetic cross-talk during pluripotency
Source: Stem Cell Reports. 2026 Sep 3;21(10):103075. doi: 10.1016/j.stemcr.2026.103075 (PMC13681314; doi:10.1016/j.stemcr.2026.103075)
Supplement: Supplementary file 1 [file mmc1.pdf]

Stem Cell Reports, Volume *21*

Supplemental Information

Methylome profiling of SetDB1-deficient ESCs reveals coordinated epigenetic cross-talk during pluripotency

Nick G.P. Bovee, Ehsan Habibi, Zicong Liu, Stefan H.A. Hoogland, Siebren Frölich, Daniëlle Diepenbroek, Kim C.M. Bosman, Julia Arand, Irina A. Maksakova, Anouk Klein Kranenbarg, Jörn Walter, Arie B. Brinkman, Klaas W. Mulder, Joop H. Jansen, Michiel Vermeulen, Matthew Lorincz, Hendrik G. Stunnenberg, and Hendrik Marks

Supplemental figures

Figure S1

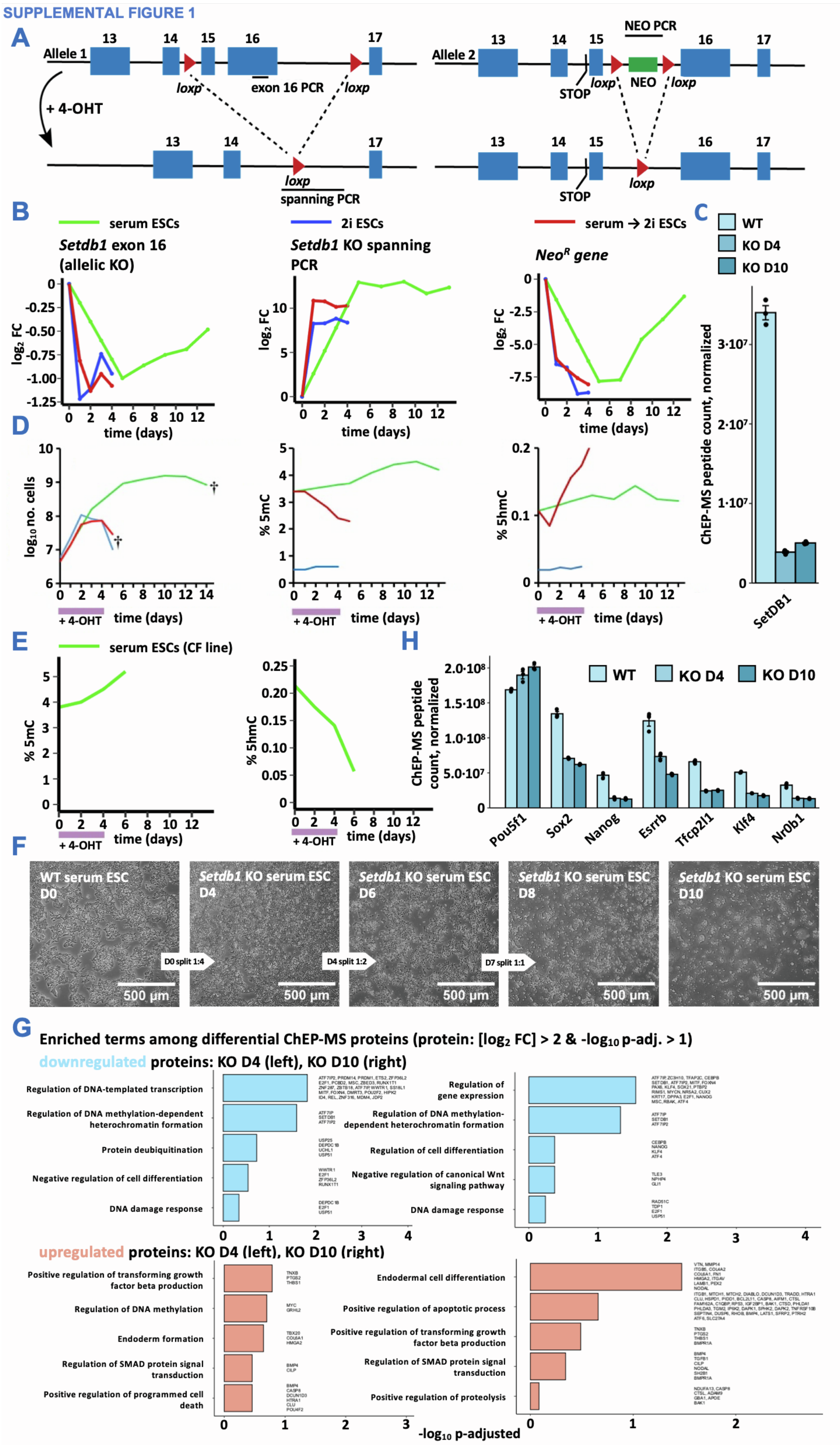

Figure S1: Loss of SetDB1 induces global DNAme increase, differentiation, induction of cell death and change of chromatin-proteome, related to Main Figure 1-4, and Supplemental Figure 2. A, Schematic representation of *Setdb1* KO strategy. Left side illustrates the conditional *Setdb1* KO strategy by exon deletion through Cre-lox recombination upon 4-OHT treatment. Right side illustrates the simultaneous deletion of a neomycin resistance gene (NEO) upon 4-OHT treatment on the other allele. **B**, qPCR data confirming functionality of 4-OHT treatment to induce *Setdb1* KO showing deletion of *Setdb1* exon 15 and 16 and removal of the NEO gene. **C**, Bar graph, similar to Fig. 3F, showing KO of SetDB1 at protein level. **D**, Line graphs of cell count (left), global 5mC (middle) and global 5hmC (right) upon induction of *Setdb1* KO in serum ESCs (green line) and 2i ESCs (blue line). 5(h)mC levels were determined by mass spectrometry. Red lines represent data of *Setdb1* KO ESCs undergoing simultaneous transition from serum to 2i culture. **E**, Line graphs similar to those of panel D (middle and right panel). Data collected from alternative *Setdb1*$^{CKO/-}$ ESC “CF line”. **F**, Microscopic images of WT and *Setdb1* KO serum ESCs on different time points post *Setdb1* KO induction. **G**, Curated lists of enriched biological processes from GO-analyses, shown as bar graphs, among differential proteins measured by ChEP-MS, defined by an absolute $\log_2$ fold-change > 2 and $-\log_{10}$ p-adjusted value > 1. Proteins related to terms indicated behind bars. Horizontal axis represents $-\log_{10}$ p-adjusted value of terms. **H**, Bar graph similar to Fig. 3F, of proteins involved in pluripotency network.

Figure S2

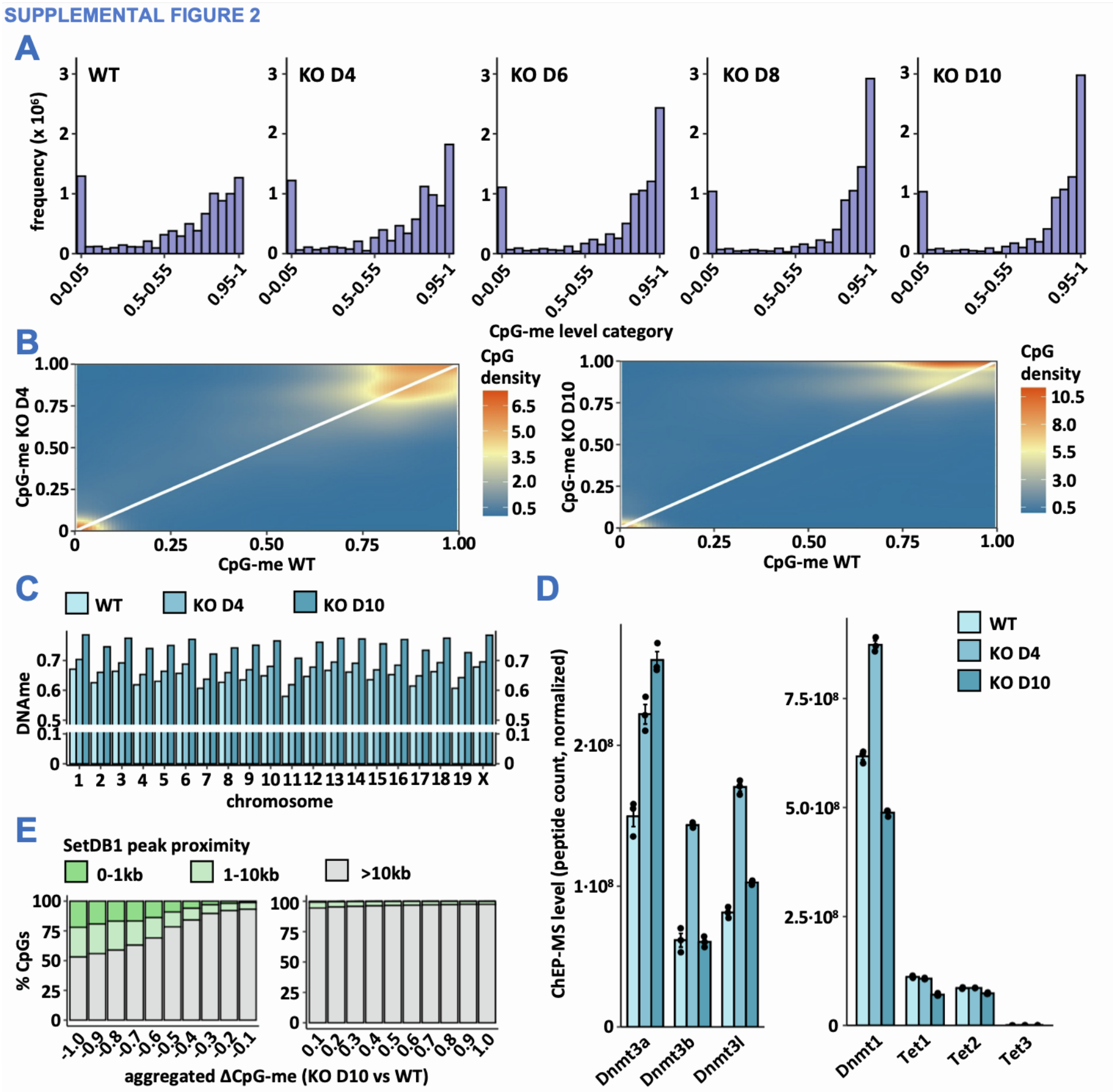
SUPPLEMENTAL FIGURE 2
A
WT
KO D4
KO D6
KO D8
KO D10
frequency (x 10⁶)
0-0.05
0.5-0.55
0.95-1
CpG-me level category
B
CpG-me KO D4
CpG-me WT
CpG density
6.5
5.0
3.5
2.0
0.5
CpG-me KO D10
10.5
8.0
5.5
3.0
C
WT
KO D4
KO D10
DNAme
chromosome
D
ChEP-MS level (peptide count, normalized)
Dnmt3a
Dnmt3b
Dnmt3l
Dnmt1
Tet1
Tet2
Tet3
E
SetDB1 peak proximity
0-1kb
1-10kb
>10kb
% CpGs
aggregated ΔCpG-me (KO D10 vs WT)

Figure S2: Loss of SetDB1 induces global DNAme increase and demethylation near specific SetDB1 targets, as observed by WGBS time course, related to Main Figure 1-2, and Supplemental Figure 1. A, Bar graph of CpG methylation levels in WT and *Setdb1* KO serum ESCs, as in Fig. 1B. **B**, Heatmap showing methylation remodeling of individual CpGs upon *Setdb1* KO day 4 and day 10, relative to WT serum ESCs, as in Fig. 1E. **C**, Bar graph showing average methylation of individual chromosomes in WT and *Setdb1* KO serum ESCs (WT, day 4 and day 10). **D**, Bar graph similar to Fig. 3F, of proteins involved in deposition and enzymatic removal of DNAme. **E**, Bar graph illustrating the percentage of proximally SetDB1-bound CpGs (categorized as 0-1kb, 1-10kb and > 10kb distance between CpG and SetDB1 peak). CpGs were split in demethylated (left) and increasingly methylated (right) groups and subsequently ranked by aggregated ΔDNAme intervals on the horizontal axis.

Figure S3

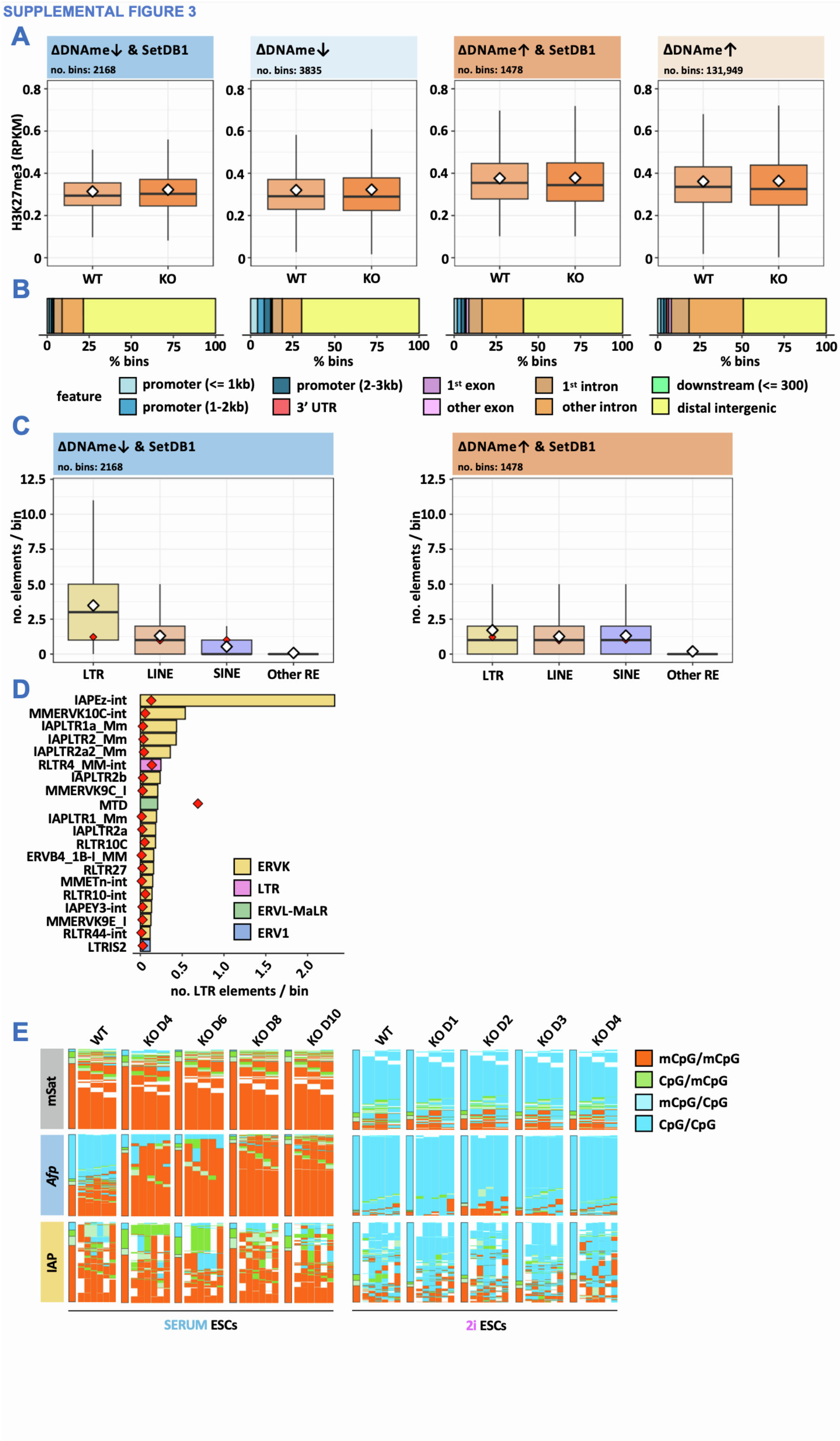

SUPPLEMENTAL FIGURE 3
A
ΔDNAme↓ & SetDB1
no. bins: 2168
ΔDNAme↓
no. bins: 3835
ΔDNAme↑ & SetDB1
no. bins: 1478
ΔDNAme↑
no. bins: 131,949
H3K27me3 (RPKM)
WT
KO
B
% bins
feature
promoter (<= 1kb)
promoter (1-2kb)
promoter (2-3kb)
3' UTR
1st exon
other exon
1st intron
other intron
downstream (<= 300)
distal intergenic
C
ΔDNAme↓ & SetDB1
no. bins: 2168
ΔDNAme↑ & SetDB1
no. bins: 1478
no. elements / bin
LTR
LINE
SINE
Other RE
D
IAPEz-int
MMERVK10C-int
IAPLTR1a_Mm
IAPLTR2_Mm
IAPLTR2a2_Mm
RLTR4_MM-int
IAPLTR2b
MMERVK9C_I
MTD
IAPLTR1_Mm
IAPLTR2a
RLTR10C
ERVB4_1B-I_MM
RLTR27
MMETn-int
RLTR10-int
IAPEY3-int
MMERVK9E_I
RLTR44-int
LTRIS2
ERVK
LTR
ERVL-MaLR
ERV1
no. LTR elements / bin
E
WT
KO D4
KO D6
KO D8
KO D10
KO D1
KO D2
KO D3
mSat
Afp
IAP
mCpG/mCpG
CpG/mCpG
mCpG/CpG
CpG/CpG
SERUM ESCs
2i ESCs

Figure S3: Targets with SetDB1-dependent DNAme and H3K9me3 are enriched with ERVKs, related to Main Figure 2 & 4. A, Boxplots of H3K27me3 levels, as in Fig. 2A. **B**, Bar plot showing the overlapping genomic features at methylome bin centers, as grouped in Fig. 2A. Genomic annotations determined by ChIPseeker. **C**, Boxplots showing the number of overlapping repeat elements per methylome bin from the SetDB1-targeted ∆DNAme↓ and ∆DNAme↑ bin groups. The other RE group includes scRNAs, rRNAs, snRNAs, srpRNAs, DNA repeats, satellites, RCs, Retroposons and tRNAs. White diamonds represent observed averages. Expected averages in any 10kb bin plotted as red diamonds. **D**, Bar graph of observed and expected frequency (red diamonds) of the 20-most-occurring LTRs in SetDB1-targeted ∆DNAme↓ bins. **E**, Heatmap representation of hairpin bisulfite sequencing data collected from WT and *SetDB1* KO serum and 2i ESCs, showing SetDB1 targeted IAPs, and control loci not targeted by SetDB1. Control loci include the *Afp* genic locus that shows an increase in DNAme following *Setdb1* KO in serum ESCs, and major satellite repeats (mSat), which remain hypermethylated in both WT and KO conditions in serum ESCs.

Figure S4

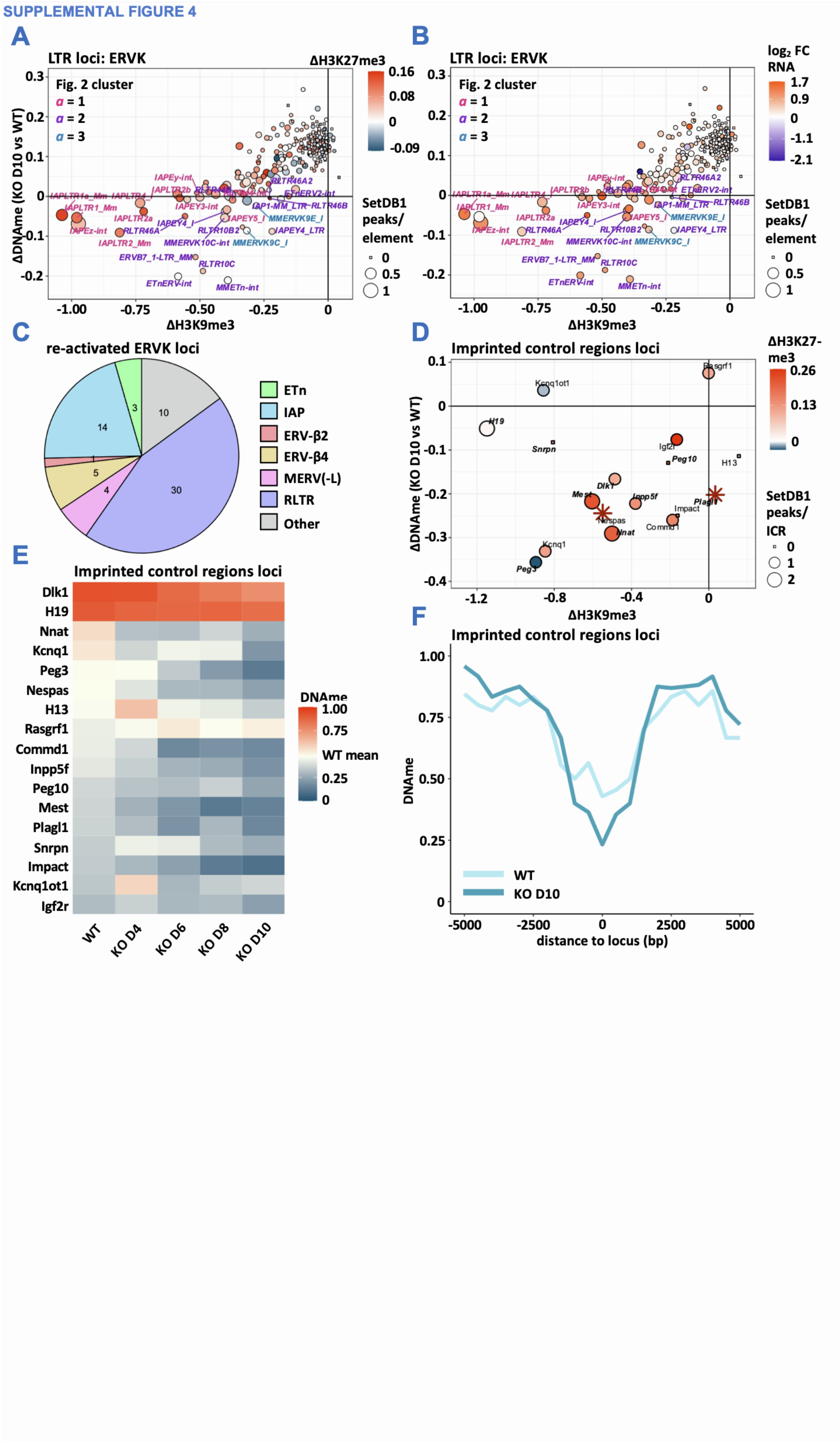

SUPPLEMENTAL FIGURE 4
A
LTR loci: ERVK
Fig. 2 cluster
ΔH3K27me3
SetDB1 peaks/ element
ΔDNAme (KO D10 vs WT)
ΔH3K9me3
B
LTR loci: ERVK
log2 FC RNA
C
re-activated ERVK loci
ETn
IAP
ERV-β2
ERV-β4
MERV(-L)
RLTR
Other
D
Imprinted control regions loci
ΔH3K27-me3
SetDB1 peaks/ ICR
E
Imprinted control regions loci
Dlk1
H19
Nnat
Kcnq1
Peg3
Nespas
H13
Rasgrf1
Commd1
Inpp5f
Peg10
Mest
Plagl1
Snrpn
Impact
Kcnq1ot1
Igf2r
WT
KO D4
KO D6
KO D8
KO D10
DNAme
WT mean
F
Imprinted control regions loci
DNAme
KO D10
distance to locus (bp)

Figure S4: ERVKs and ICRs with SetDB1-dependent DNAme-H3K9me3 are reactivated in serum ESCs following SetDB1 loss, related to Main Figure 2, and Supplemental Figure 5-6. A, Scatter plot of LTR-ERVKs showing ΔH3K9me3 (ΔRPKM, KO vs WT), ΔDNAme (KO day 10 vs WT), and ΔH3K27me3 (ΔRPKM, KO vs WT) of ERVKs in serum ESCs upon *Setdb1* KO. Number of SetDB1 ChIP-seq peaks per ERVK copy (1kb proximity) displayed as point size. Color of name tag reflects clusters of Fig. 2B in which ERVK is predominantly found. **B**, Scatter plot similar to panel A, but color scale now represents difference of expression after *Setdb1* KO, as $\log_2$ fold-change. **C**, Pie chart illustrating the number of increasingly expressed ERVKs upon *Setdb1* KO in serum ESCs from different ERVK-groups (fold-change > 1.5). **D**, Scatter plot of ICRs, similar to panel A. Names in italic font if imprinted gene was transcriptionally upregulated after *Setdb1* KO. **E**, Heatmap of DNAme levels at ICRs in WT and *Setdb1* KO serum ESCs. ICRs are organized by WT DNAme levels. **F**, Line graph illustrating median DNAme levels at ICRs (zero distance) and their immediate genomic surroundings.

Figure S5

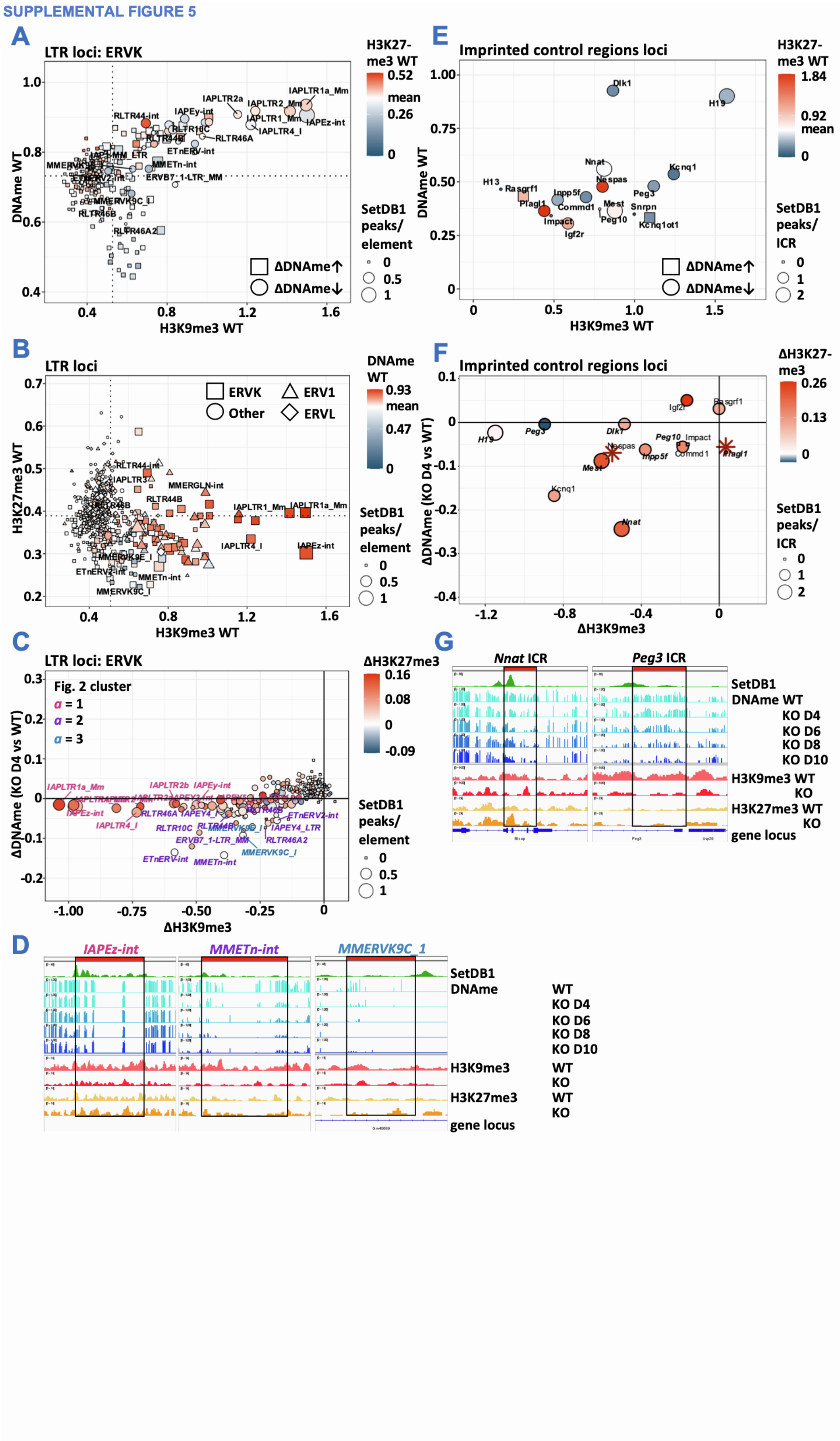

SUPPLEMENTAL FIGURE 5
A
LTR loci: ERVK
DNAme WT
H3K9me3 WT
H3K27-me3 WT
0.52
mean
0.26
0
SetDB1 peaks/ element
ΔDNAme↑
ΔDNAme↓
B
LTR loci
H3K27me3 WT
ERVK
ERV1
Other
ERVL
DNAme WT
0.93
mean
0.47
C
LTR loci: ERVK
Fig. 2 cluster
ΔDNAme (KO D4 vs WT)
ΔH3K9me3
ΔH3K27me3
0.16
0.08
-0.09
D
IAPEz-int
MMETn-int
MMERVK9C_I
SetDB1
DNAme
WT
KO D4
KO D6
KO D8
KO D10
H3K9me3
H3K27me3
gene locus
E
Imprinted control regions loci
H3K27-me3 WT
1.84
0.92
SetDB1 peaks/ ICR
F
ΔH3K27-me3
0.26
0.13
G
Nnat ICR
Peg3 ICR
DNAme WT
H3K9me3 WT
H3K27me3 WT
KO

Figure S5: ERVK and ICR targets with SetDB1-dependent DNAme-H3K9me3 lose both repressive marks following *Setdb1* KO, related to Main Figure 2, and Supplemental Figure 4 & 6. **A**, Scatter plot of LTR-ERVKs showing WT H3K9me3 (RPKM), WT DNAme, and WT H3K27me3 (RPKM) levels at ERVKs in serum ESCs. Number of SetDB1 ChIP-seq peaks per ERVK copy (1kb proximity) displayed as point size. Dotted lines represent averages of their respectively axis parameters. **B**, Scatter plot of all LTRs, similar to panel A, but parameters of vertical axis and color scale swapped. **C**, Scatter plot similar to Supplemental Fig. 4A, but vertical axis changed to ΔDNAme KO day 4 vs WT. **D**, IGV screenshots of IAPEz-int (Chr11:120,212,299-120,218,751), MMETn-int (Chr10:83,236,248-83,240,631) and MMERVK9C_1 (Chr10:70,673,637-70,675,369) loci showing WGBS and ChIP-seq signals and overlap with genic loci. Color of name tags transferred from panel C. **E**, Scatter plot of ICRs, similar to panel A. **F** Scatter plot of ICRs, similar to Supplemental Fig. 4A. Names in italic font if imprinted gene was transcriptionally upregulated after *Setdb1* KO. **G**, IGV screenshots of *Nnat* and *Peg3* ICR, similar to panel D.

Figure S6

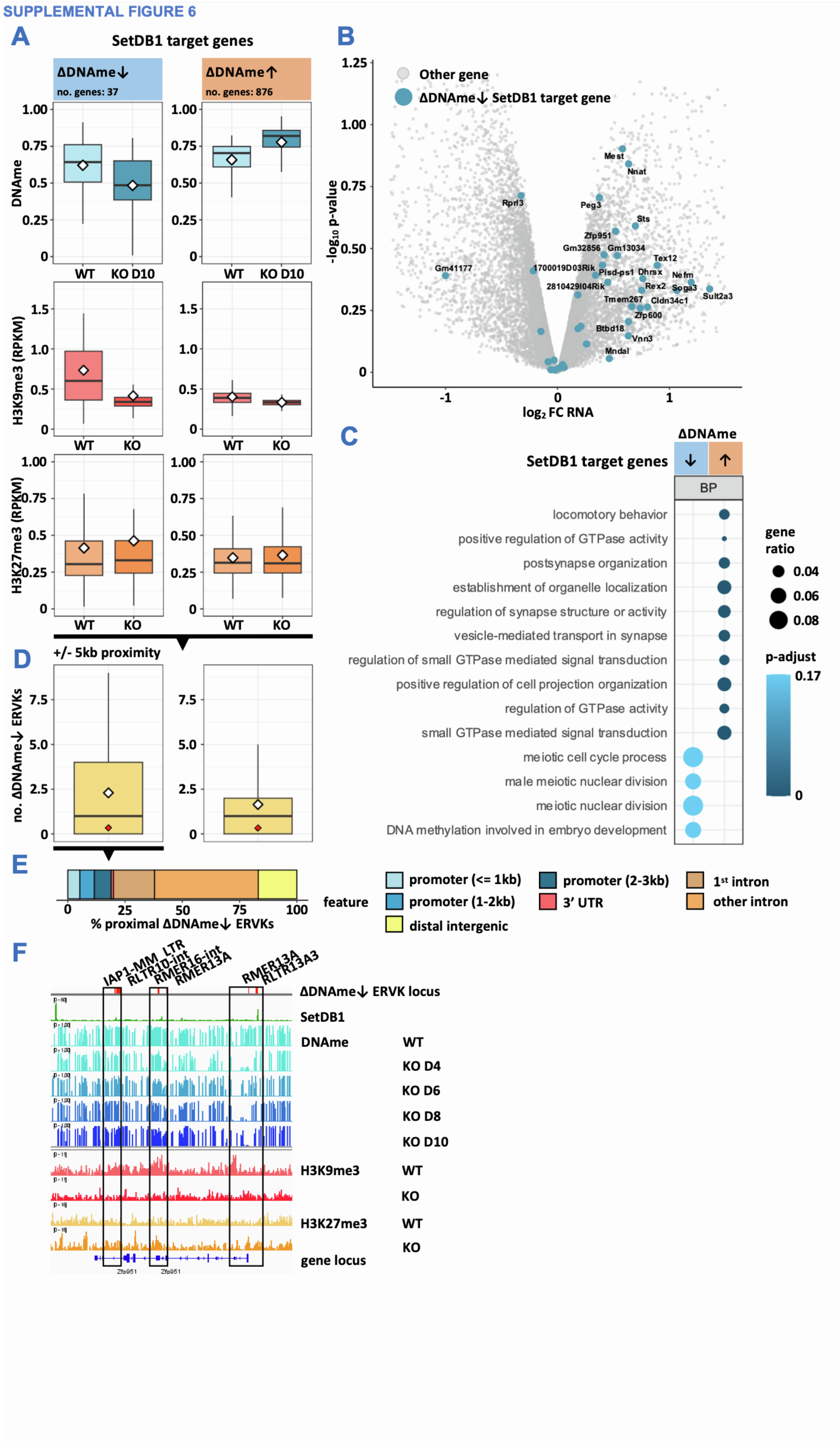

SUPPLEMENTAL FIGURE 6
A
SetDB1 target genes
ΔDNAme↓
no. genes: 37
ΔDNAme↑
no. genes: 876
DNAme
WT
KO D10
H3K9me3 (RPKM)
WT
KO
H3K27me3 (RPKM)
+/- 5kb proximity
B
Other gene
ΔDNAme↓ SetDB1 target gene
-log10 p-value
log2 FC RNA
Mest
Nnat
Rprl3
Peg3
Sts
Zfp951
Gm32856
Gm13034
Tex12
Gm41177
1700019D03Rik
Pisd-ps1
Dhrsx
Nefm
2810429I04Rik
Rex2
Soga3
Sult2a3
Tmem267
Cldn34c1
Zfp600
Btbd18
Vnn3
Mndal
C
ΔDNAme
SetDB1 target genes
BP
locomotory behavior
positive regulation of GTPase activity
postsynapse organization
establishment of organelle localization
regulation of synapse structure or activity
vesicle-mediated transport in synapse
regulation of small GTPase mediated signal transduction
positive regulation of cell projection organization
regulation of GTPase activity
small GTPase mediated signal transduction
meiotic cell cycle process
male meiotic nuclear division
meiotic nuclear division
DNA methylation involved in embryo development
gene ratio
0.04
0.06
0.08
p-adjust
0.17
0
D
no. ΔDNAme↓ ERVKs
E
% proximal ΔDNAme↓ ERVKs
feature
promoter (<= 1kb)
promoter (1-2kb)
promoter (2-3kb)
3' UTR
1st intron
other intron
distal intergenic
F
IAP1-MM_LTR
RLTR10-int
RMER16-int
RMER13A
RMER13A
RLTR13A3
ΔDNAme↓ ERVK locus
SetDB1
DNAme
WT
KO D4
KO D6
KO D8
KO D10
H3K9me3
WT
KO
H3K27me3
WT
KO
gene locus
Zfp951

Figure S6: Genes with SetDB1-dependent DNAme-H3K9me3 are derepressed in serum ESCs following SetDB1 loss, related to Main Figure 2, and Supplemental Figure 4-5. A, Boxplots of DNAme, H3K9me3, and H3K27me3 levels of SetDB1-targeted genes in WT and *Setdb1* KO serum ESCs, grouped by methylation change at day 10 of *Setdb1* KO. White diamonds represent averages. **B**, Volcano plot of differentially expressed genes in *Setdb1* KO serum ESCs compared to WT serum ESCs, as observed in RNA-seq data. Demethylated SetDB1-target genes are highlighted in blue. **C**, Dot plot showing biological processes enriched among SetDB1-targeted genes (demethylated or increasingly methylated by day 10 of *Setdb1* KO), as determined by GO-analysis. **D**, Boxplots showing number of demethylated ERVK copies within 5kb distance of SetDB1-target genes, as categorized in panel A. White diamonds represent observed averages. Red diamonds represent expected number of demethylated ERVKs near any gene. **E**, Barplot showing genomic positions of demethylated ERVK elements near demethylated SetDB1 target genes. Genomic annotations determined by ChIPseeker. **F**, IGV screenshots of the *Zfp951* locus, showing WGBS and ChIP-seq signals. Demethylated ERVK (by day 10 of *Setdb1* KO) loci indicated in red above IGV tracks.

Figure S7

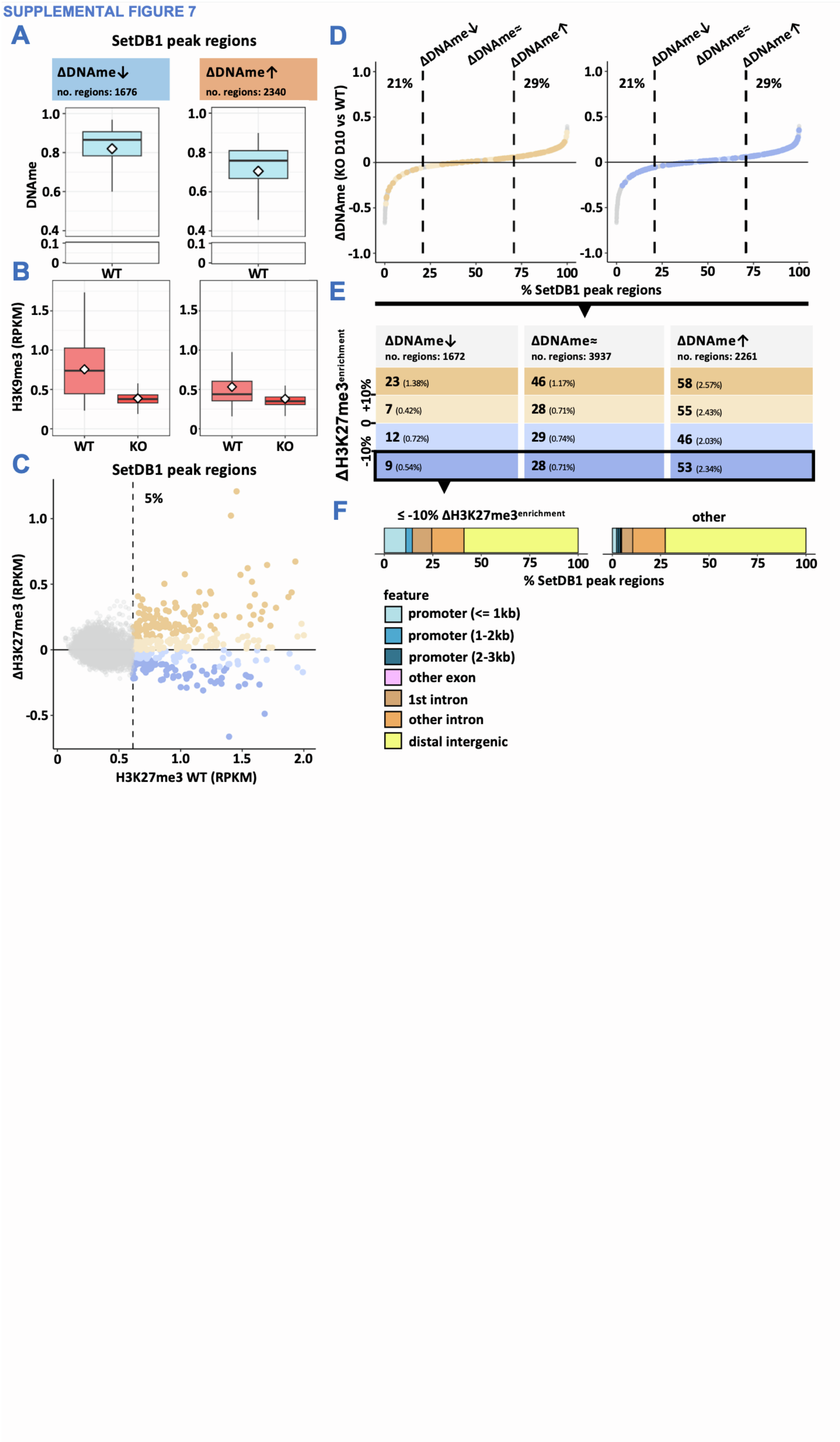

SUPPLEMENTAL FIGURE 7
A
SetDB1 peak regions
ΔDNAme↓
no. regions: 1676
ΔDNAme↑
no. regions: 2340
DNAme
WT
B
H3K9me3 (RPKM)
WT
KO
C
SetDB1 peak regions
5%
ΔH3K27me3 (RPKM)
H3K27me3 WT (RPKM)
D
ΔDNAme↓
ΔDNAme≈
ΔDNAme↑
21%
29%
ΔDNAme (KO D10 vs WT)
% SetDB1 peak regions
E
ΔDNAme↓
no. regions: 1672
ΔDNAme≈
no. regions: 3937
ΔDNAme↑
no. regions: 2261
ΔH3K27me3enrichment
+10%
0
-10%
23 (1.38%)
46 (1.17%)
58 (2.57%)
7 (0.42%)
28 (0.71%)
55 (2.43%)
12 (0.72%)
29 (0.74%)
46 (2.03%)
9 (0.54%)
28 (0.71%)
53 (2.34%)
F
≤ -10% ΔH3K27me3enrichment
other
% SetDB1 peak regions
feature
promoter (<= 1kb)
promoter (1-2kb)
promoter (2-3kb)
other exon
1st intron
other intron
distal intergenic

Figure S7: SetDB1-mediated H3K27me3-regulation is uncoupled from its DNAme-H3K9me3 axis, related to Main Figure 3. A, Boxplots of WT DNAme levels in serum ESCs within SetDB1 peak regions as defined in Fig. 3A (10kb centered on ChIP-seq peak summits). White diamonds represent averages. **B**, Boxplots of H3K9me3 levels in WT and *Setdb1* KO serum ESCs within SetDB1 peak regions as defined in Fig. 3A. White diamonds represent observed averages. **C**, Scatterplot of SetDB1 peak regions showing H3K27me3 in WT and *Setdb1* KO serum ESCs. Peak regions were further filtered relative to panels A and B to exclude regions with excessive H3K27me3 by removing the top 2% of WT H3K27me3 regions. Dashed vertical line represents 95th quantile of filtered regions. Colors match H3K27me3 remodeling category: orange, > 10% H3K27me3 gain; light orange, 0-10% gain; light blue, 0-10% loss; blue, >10% loss. **D**, Remapping of H3K27me3 remodeling categories from panel C to ΔDNAme-based ordering of SetDB1 peak regions of Fig. 3A. **E**, Table showing number of H3K27me3-enriched SetDB1 peak region categories from panel C within ΔDNAme-based categories of SetDB1 peak regions as shown in Fig. 3A. **F**, Barplot showing genomic positions of SetDB1 peaks, highlighting a subset of peaks whose regions lose H3K27me3 enrichment after *Setdb1* KO. Genomic annotations determined by ChIPseeker.

Figure S8

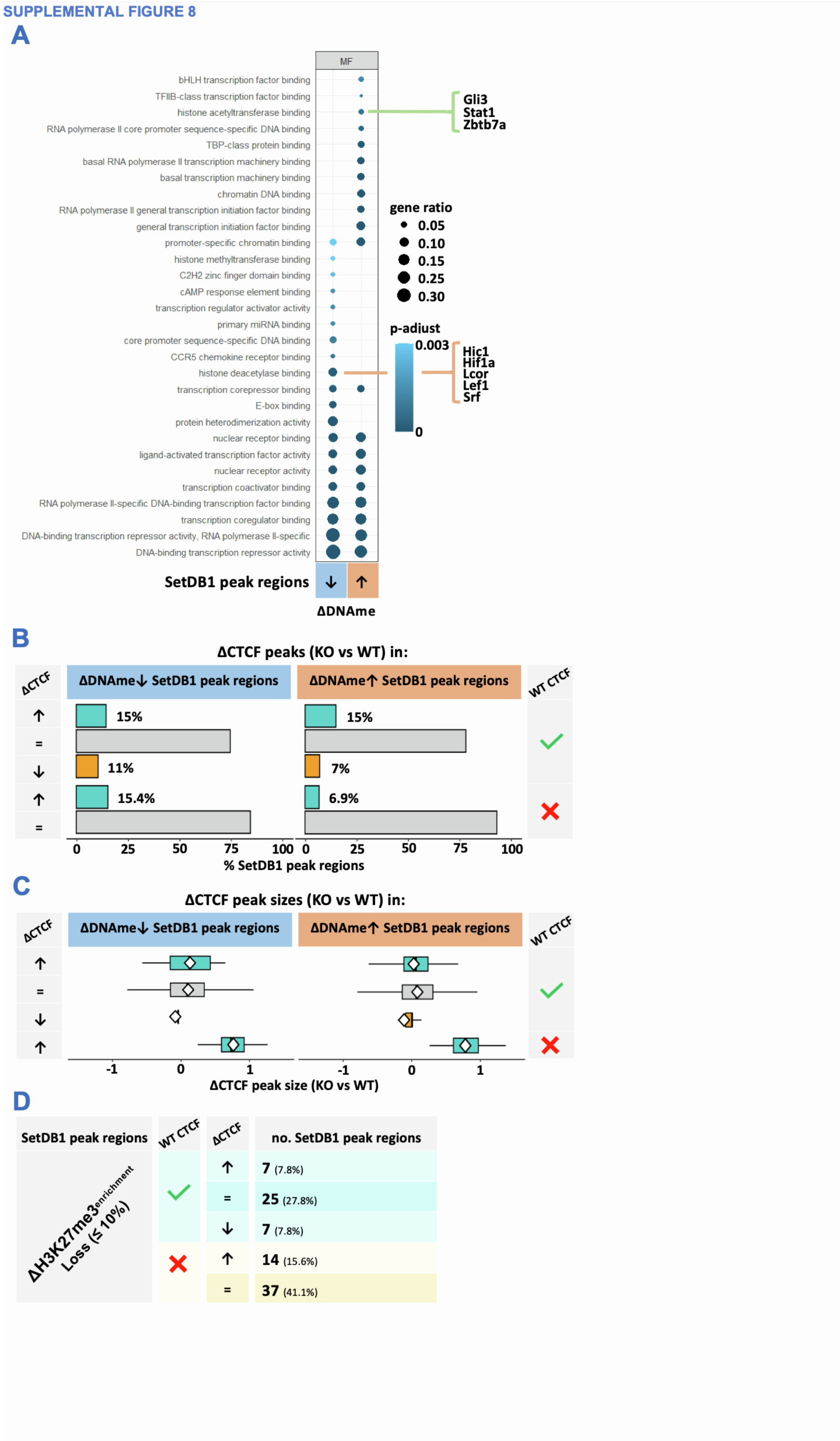
SUPPLEMENTAL FIGURE 8
A
MF
bHLH transcription factor binding
TFIIB-class transcription factor binding
histone acetyltransferase binding
RNA polymerase II core promoter sequence-specific DNA binding
TBP-class protein binding
basal RNA polymerase II transcription machinery binding
basal transcription machinery binding
chromatin DNA binding
RNA polymerase II general transcription initiation factor binding
general transcription initiation factor binding
promoter-specific chromatin binding
histone methyltransferase binding
C2H2 zinc finger domain binding
cAMP response element binding
transcription regulator activator activity
primary miRNA binding
core promoter sequence-specific DNA binding
CCR5 chemokine receptor binding
histone deacetylase binding
transcription corepressor binding
E-box binding
protein heterodimerization activity
nuclear receptor binding
ligand-activated transcription factor activity
nuclear receptor activity
transcription coactivator binding
RNA polymerase II-specific DNA-binding transcription factor binding
transcription coregulator binding
DNA-binding transcription repressor activity, RNA polymerase II-specific
DNA-binding transcription repressor activity
Gli3
Stat1
Zbtb7a
gene ratio
0.05
0.10
0.15
0.25
0.30
p-adjust
0.003
0
Hic1
Hif1a
Lcor
Lef1
Srf
SetDB1 peak regions ↓ ↑
ΔDNAme
B
ΔCTCF peaks (KO vs WT) in:
ΔCTCF
ΔDNAme↓ SetDB1 peak regions
ΔDNAme↑ SetDB1 peak regions
WT CTCF
↑ 15% 15%
=
↓ 11% 7%
↑ 15.4% 6.9%
=
0 25 50 75 100 0 25 50 75 100
% SetDB1 peak regions
C
ΔCTCF peak sizes (KO vs WT) in:
ΔCTCF
ΔDNAme↓ SetDB1 peak regions
ΔDNAme↑ SetDB1 peak regions
WT CTCF
↑
=
↓
↑
-1 0 1 -1 0 1
ΔCTCF peak size (KO vs WT)
D
SetDB1 peak regions
WT CTCF
ΔCTCF
no. SetDB1 peak regions
ΔH3K27me3enrichment Loss (≤ 10%)
↑ 7 (7.8%)
= 25 (27.8%)
↓ 7 (7.8%)
↑ 14 (15.6%)
= 37 (41.1%)

Figure S8: SetDB1-mediated CTCF-regulation is uncoupled from its DNAme-H3K9me3 and H3K27me3 axes, related to Main Figure 3. A, Dot plot showing molecular functions of TFs whose motifs are enriched in SetDB1 peak region categories from Fig. 3A, as determined by GO-analysis. **B**, Bar plots of SetDB1 peak regions, as defined in Fig. 3A and further grouped by the presence of CTCF WT peaks, showing the percentage of peak regions with changing (gain, loss) or equal (same) number of CTCF peaks upon *Setdb1* KO in serum ESCs. **C**, Boxplots showing change in average CTCF peak size between WT and *Setdb1* KO serum ESCs of categories denoted in panel B. White diamonds represent observed averages. **D**, Table summarizing CTCF peak remodeling at H3K27me3-mediated SetDB1 peak regions, as defined in Supplemental Fig. 7. Numbers reflect number and percentage of H3K27me3-mediated SetDB1 peak regions within categories.

Figure S9

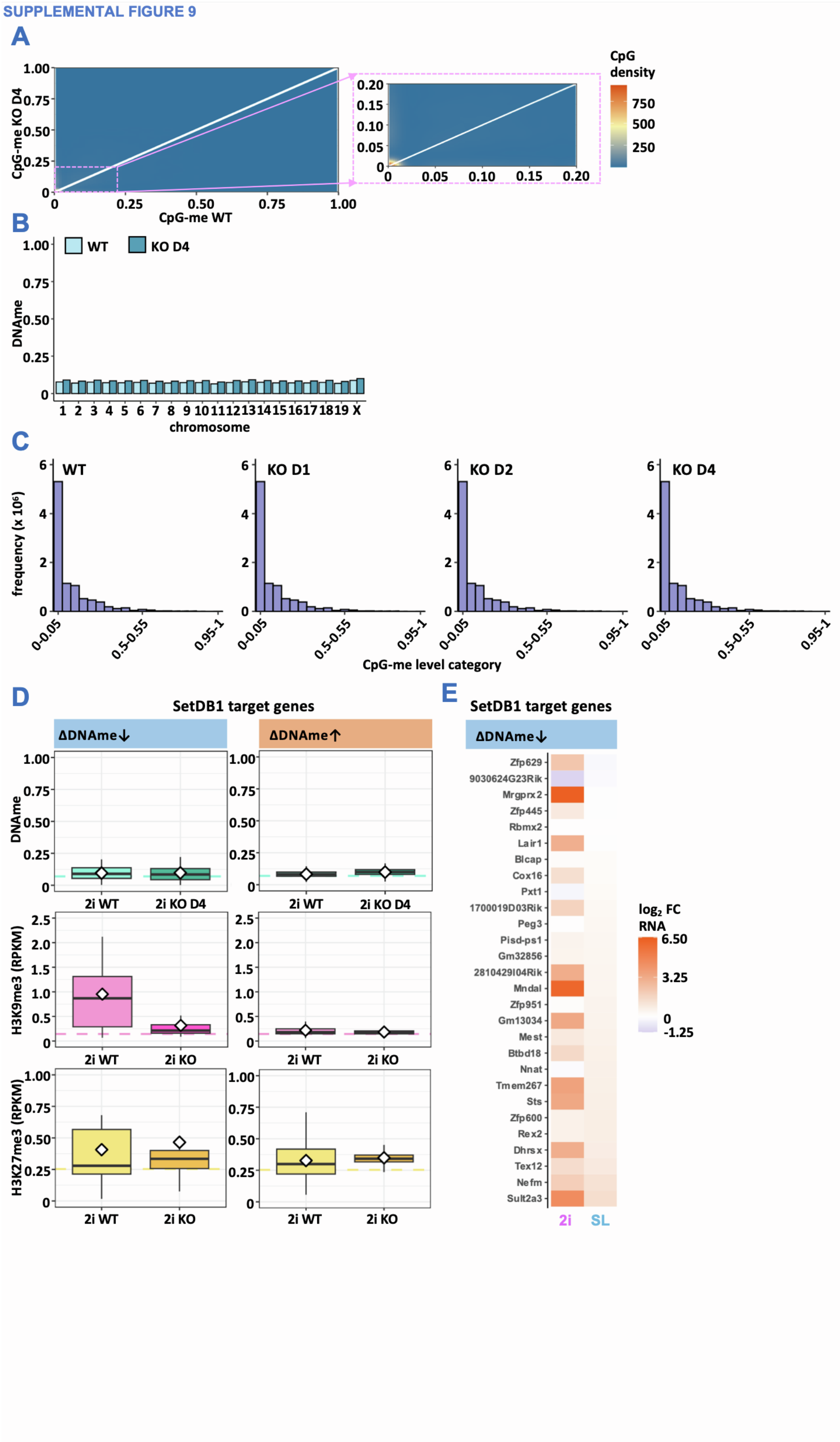

SUPPLEMENTAL FIGURE 9
A
CpG-me KO D4
CpG-me WT
CpG density
750
500
250
B
WT
KO D4
DNAme
chromosome
C
WT
KO D1
KO D2
KO D4
frequency (x 10⁶)
CpG-me level category
D
SetDB1 target genes
ΔDNAme↓
ΔDNAme↑
DNAme
2i WT
2i KO D4
H3K9me3 (RPKM)
2i KO
H3K27me3 (RPKM)
E
SetDB1 target genes
ΔDNAme↓
Zfp629
9030624G23Rik
Mrgprx2
Zfp445
Rbmx2
Lair1
Blcap
Cox16
Pxt1
1700019D03Rik
Peg3
Pisd-ps1
Gm32856
2810429I04Rik
Mndal
Zfp951
Gm13034
Mest
Btbd18
Nnat
Tmem267
Sts
Zfp600
Rex2
Dhrsx
Tex12
Nefm
Sult2a3
2i
SL
log₂ FC RNA
6.50
3.25
0
-1.25

Figure S9: Hypomethylated 2i ESCs rely on SetDB1-mediated H3K9me3 to silence ERVK-proximal genes, related to Main Figure 4, and Supplemental Figure 1-3. A, Heatmaps showing methylation remodeling of individual CpGs upon *Setdb1* KO, relative to WT in 2i ESCs. CpG count density on color scale. Diagonal white lines represent slope = 1. **B**, Bar graph showing average methylation of chromosomes in WT and *Setdb1* KO in 2i ESCs (WT and day 4). **C**, Bar graph of CpG methylation levels in WT and *Setdb1* KO 2i ESCs, similar to Supplemental Fig. 2A. **D**, Boxplots of DNAme, H3K9me3, and H3K27me3 levels in WT and KO 2i ESCs at SetDB1 target genes from serum ESCs (Supplemental Fig. 6A: ΔDNAme↓ category). Dotted lines represent median WT levels in 2i ESCs. White diamonds represent averages. **E**, Heat map showing expression difference (KO vs WT) of SetDB1 target genes defined in panel D. Expression differences are denoted as $\log_2$ fold-change, and shown for ESCs cultured in serum and 2i.

Figure S10

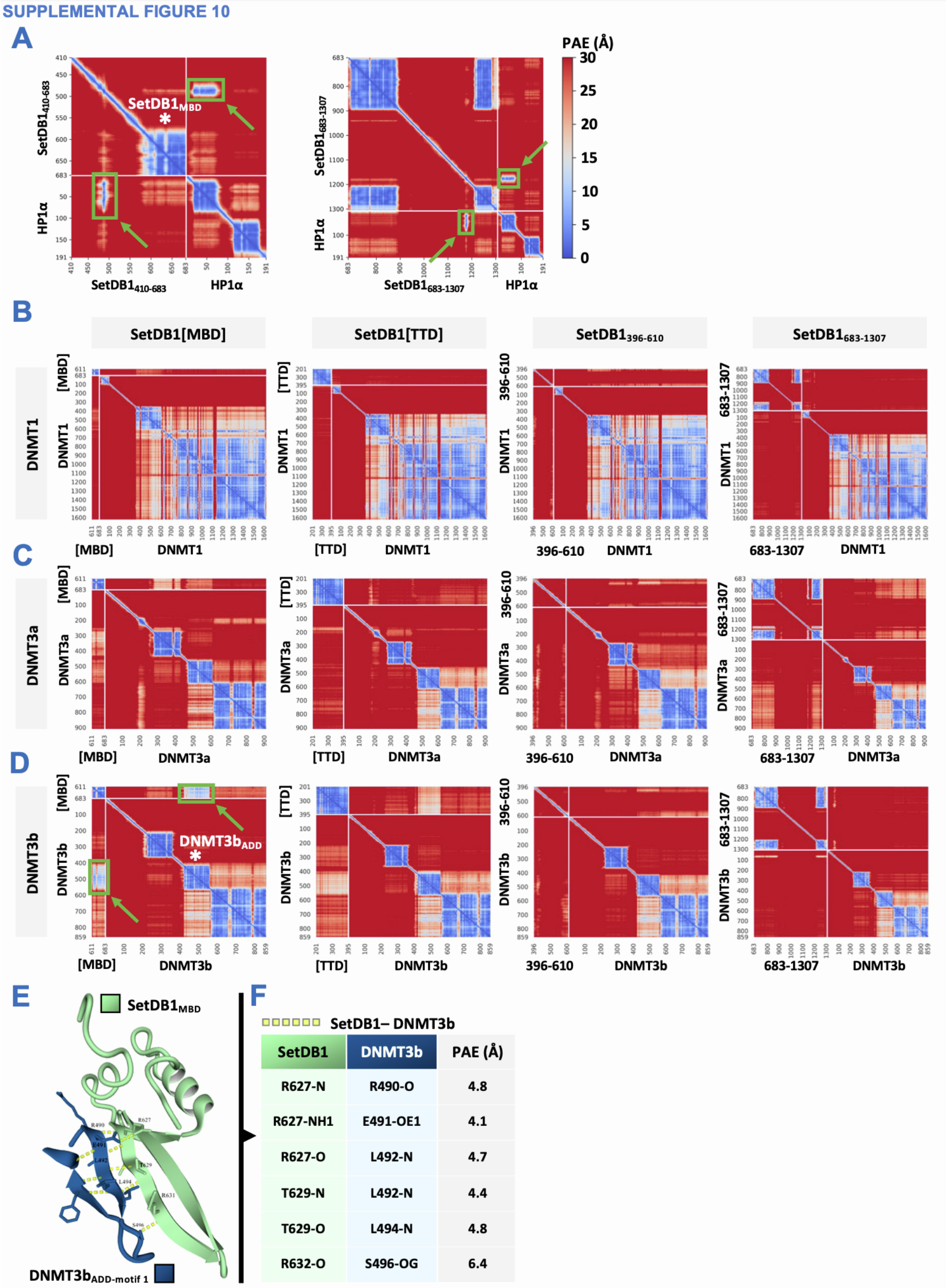

SetDB1	DNMT3b	PAE (Å)
R627-N	R490-O	4.8
R627-NH1	E491-OE1	4.1
R627-O	L492-N	4.7
T629-N	L492-N	4.4
T629-O	L494-N	4.8
R632-O	S496-OG	6.4

Figure S10: Modeling of interactions predicted by AlphaFold3 between domains of SetDB1 and full-length DNMT1, -3a, and -3b, related to Main Figure 5, and Supplemental Figure 11-12. A, PAE heatmaps, similar to Fig. 5A, between SetDB1 residues 410-683 and 683-1307 and full-length HP1α, as positive control. All amino acid sequences from mouse. **B-D**, PAE heatmaps, similar to Fig. 5A, between the MBD and TTD of SetDB1, as well as SetDB1 flexible regions spanning amino acids 396-610 (Flex 1) and 683-1307 (Flex 2), and full-length DNMT1, -3a, and -3b. All amino acid sequences from mouse. **E**, Close-up view of structure of interacting residues from Fig. 5B. Yellow-dotted lines indicate hydrogen bonds, and interacting residues are labeled. **F**, Summary of residues involved in SetDB1-DNMT3b interaction as modeled in panel E, including residue number, interacting atoms, and PAE values in ängstrom.

Figure S11

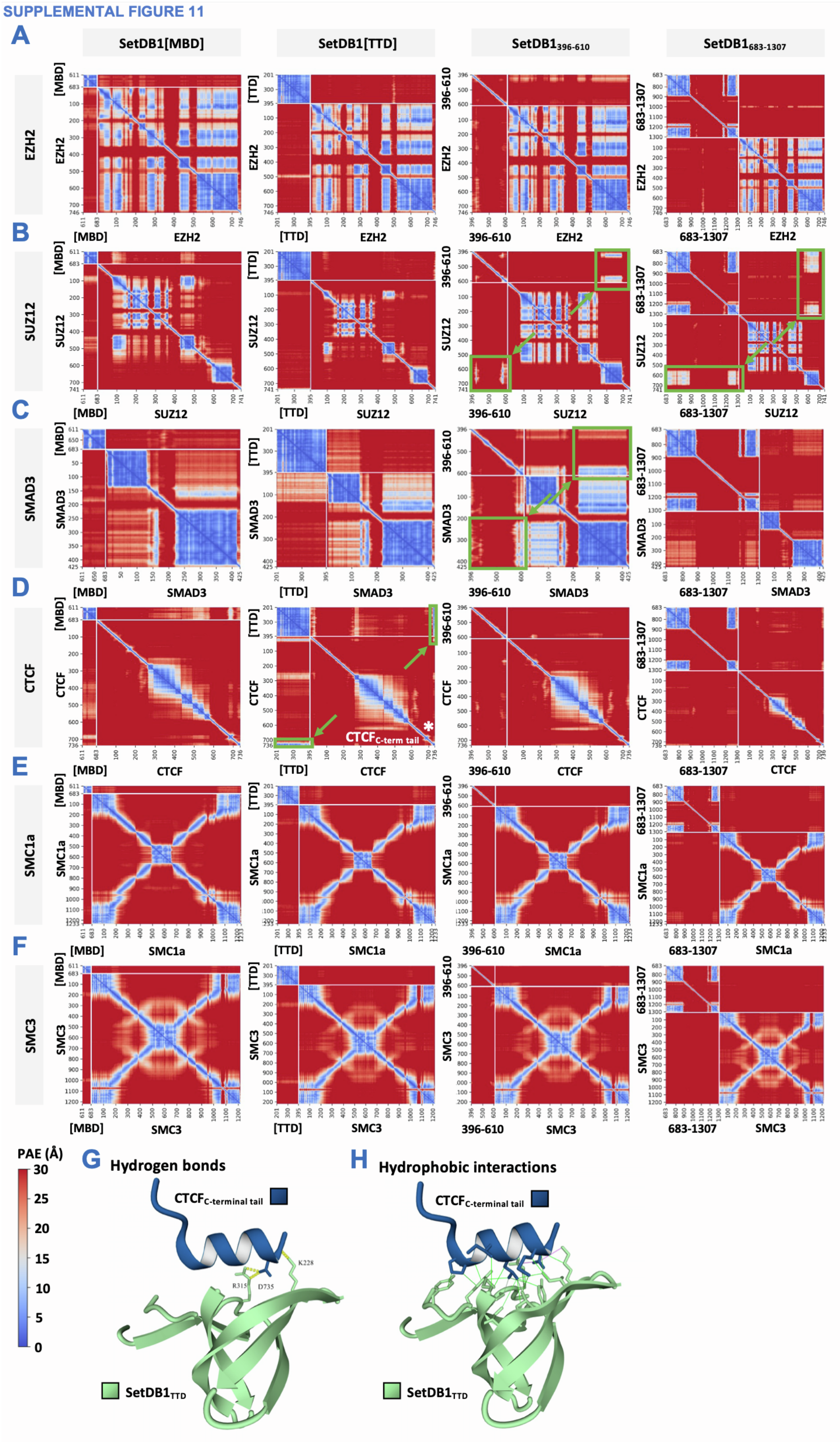
SUPPLEMENTAL FIGURE 11
A
SetDB1[MBD]
SetDB1[TTD]
SetDB1396-610
SetDB1683-1307
EZH2
B
SUZ12
C
SMAD3
D
CTCF
CTCFC-term tail
E
SMC1a
F
SMC3
PAE (Å)
G
Hydrogen bonds
CTCFC-terminal tail
SetDB1TTD
H
Hydrophobic interactions
CTCFC-terminal tail
SetDB1TTD

Figure S11: Modeling of interactions predicted by AlphaFold3 between domains of SetDB1 and full-length EZH2, SUZ12, SMAD3, CTCF, and SMC1a and -3, related to Main Figure 5, and Supplemental Figure 10 & 12. A-F, PAE heatmaps, similar to Fig. 5A, between the MBD and TTD of SetDB1, as well as SetDB1 flexible regions spanning amino acids 396-610 (Flex 1) and 683-1307 (Flex 2), and full-length EZH2, SUZ12, SMAD3, CTCF, SMC1a, and SMC3. All amino acid sequences from mouse. **G-H**, Close-up view of structure of interacting residues from panel D. Yellow-dotted lines in panel G indicate hydrogen bonds, and interacting residues are labeled. Thin lines in panel H indicate hydrophobic interactions.

Figure S12

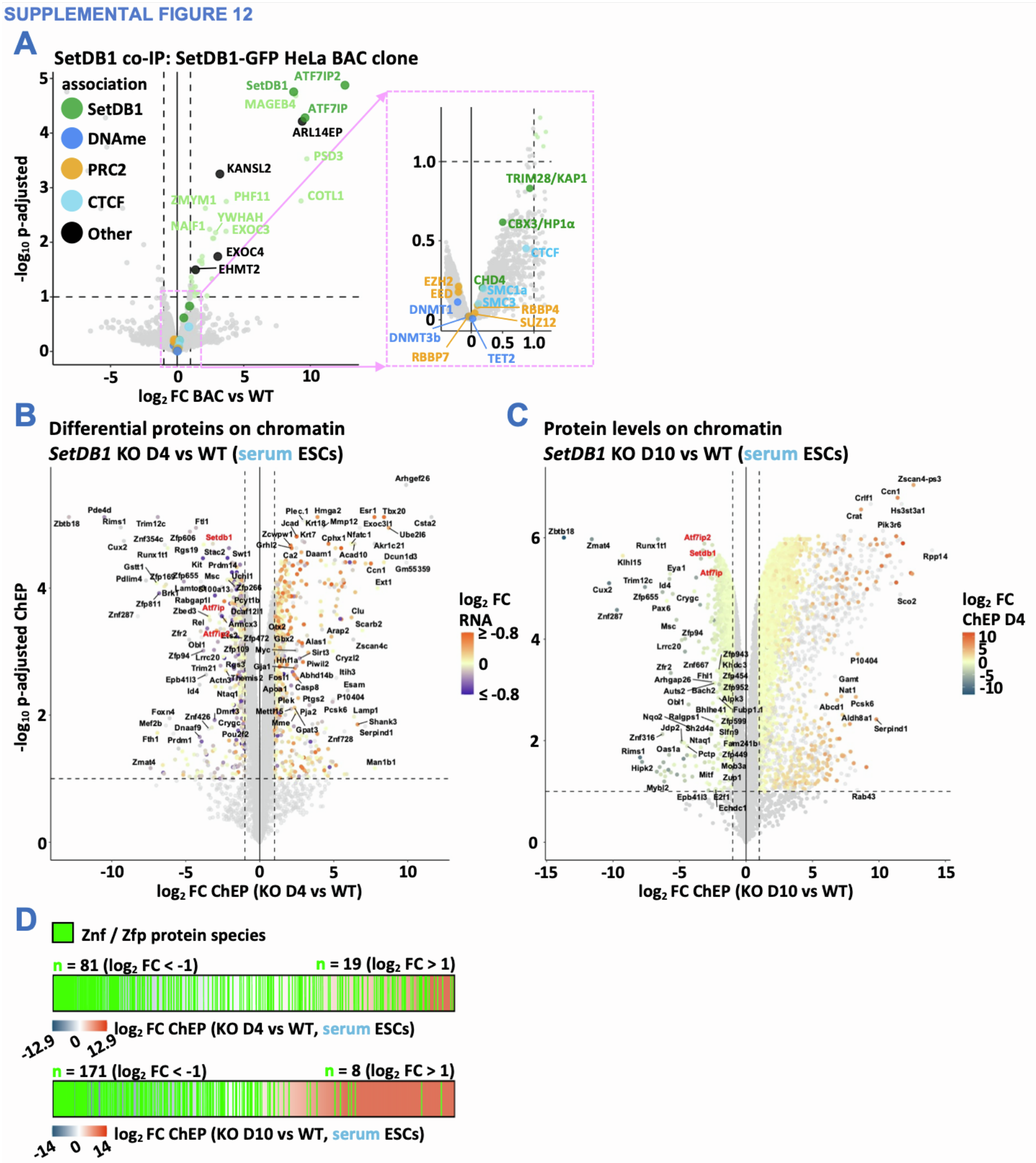

SUPPLEMENTAL FIGURE 12
A
SetDB1 co-IP: SetDB1-GFP HeLa BAC clone
association
SetDB1
DNAme
PRC2
CTCF
Other
-log10 p-adjusted
log2 FC BAC vs WT
B
Differential proteins on chromatin
SetDB1 KO D4 vs WT (serum ESCs)
-log10 p-adjusted ChEP
log2 FC ChEP (KO D4 vs WT)
log2 FC RNA
≥ -0.8
0
≤ -0.8
C
Protein levels on chromatin
SetDB1 KO D10 vs WT (serum ESCs)
log2 FC ChEP (KO D10 vs WT)
log2 FC ChEP D4
D
Znf / Zfp protein species
n = 81 (log2 FC < -1)
n = 19 (log2 FC > 1)
log2 FC ChEP (KO D4 vs WT, serum ESCs)
n = 171 (log2 FC < -1)
n = 8 (log2 FC > 1)
log2 FC ChEP (KO D10 vs WT, serum ESCs)

Figure S12: Proteomics of SetDB1 reveal its interactome, as well as chromatome remodeling following SetDB1 loss, related to Main Figure 5, and Supplemental Figure 10-11. A, Volcano plot of proteins associated with SetDB1, as measured by co-IP MS on nuclear extracts from SetDB1-GFP BAC clone HeLa cells compared to WT HeLa cells. Enrichments are denoted as $\log_2$ fold-change in BAC vs WT extracts, accompanied by $-\log_{10}$ adjusted p-values (vertical axis). Thresholds for enrichment ($\log_2$ fold-change ≥ 1) and significance ($-\log_{10}$ p-adjusted ≥ 1) indicated as dashed lines. **B**, Volcano plot of differential proteins on chromatin after 4 days of *Setdb1* KO in serum ESCs, compared to WT serum ESCs, as measured by ChEP-MS. Significance and differentiality thresholds are indicated by dashed lines corresponding to a $-\log_{10}$ p-adjusted of 1, and $\log_2$ fold-change cutoffs of -1 and 1, respectively. Color of data points are indicated if proteins were significantly differential, and represents their $\log_2$ fold-change as observed in RNA-seq data (at day 5 of *Setdb1* KO induction: Barral et al. 2017). **C**, Volcano plot similar to panel A, but $\log_2$ fold-changes represent difference at 10 days of *Setdb1* KO in serum ESCs, compared to WT. Color scale represents $\log_2$ fold-change difference at 4 days of *Setdb1* KO. Grey dot above thresholds indicates protein is recruited/lost to chromatin at day 10, but not yet at day 4 post-KO induction. **D**, Heatmaps showing $\log_2$ fold-changes of chromatin-bound proteins at day 4 and 10 after *Setdb1* KO induction in serum ESCs, as measured by ChEP-MS. Proteins in heatmaps are ordered by their $\log_2$ fold-change, from low to high. If protein is encoded by *Znf* or *Zfp* gene, it is indicated in green.

Supplemental notes

Supplemental Note 1: SetDB1 loss triggers DNAme-H3K9me3 erasure and reactivation of targeted ERVKs and nearby genes, and ICRs, related to Main Figure 1-2, and Figure S3-S6.

Our binning approach applied to the methylome data collected from SetDB1-deficient serum ESCs does not easily allow investigation of transcriptional responses from individual genomic elements. To facilitate such analysis, we also projected the data on the genomic loci directly. Consistent with results at 10kb binning scale described in Figures 1 and 2, various ERVK loci (11-7710bp, median size: 440bp) are targeted by SetDB1 and enriched with DNAme and H3K9me3 (Supplemental Fig. 5A). Among these, IAPEz elements show the greatest SetDB1 targeting and enrichment of DNAme and H3K9me3. A minority of LTR regions of IAPs and other ERVKs are also marked with H3K27me3. A similar co-occurrence of H3K9me3 and H3K27me3 was observed in PGCs, including at hypermethylated IAPs (Liu et al., 2014). However, H3K27me3 coverage is generally greatest on ERVKs and other LTR subfamilies that lack H3K9me3 (and DNAme) enrichment in serum ESCs (Supplemental Fig. 5B). This aligns with previous findings that H3K9me3 and H3K27me3 mostly mark distinct genomic regions in ESCs (Fei et al., 2015), and supports the hypothesis that H3K27me3 may repress retrotransposons that are not marked by SetDB1-dependent H3K9me3 and DNAme (Prokopuk et al., 2017). Notably, H3K27me3-enriched LTRs are rarely bound by SetDB1 compared to DNAme-H3K9me3-enriched LTRs (Supplemental Fig. 5A), suggesting that SetDB1 preferentially regulates its retrotransposon targets via H3K9me3 in combination with DNAme. Furthermore, consistent with Figures 1 and 2, strongly targeted ERVKs were demethylated and completely lost their H3K9me3 enrichment after *Setdb1* KO (Supplemental Fig. 4A; Supplemental Fig. 5C-D). Following the loss of DNAme and H3K9me3 on ERVKs, we noticed a minor increase in H3K27me3 (Supplemental Fig. 4A), which is in line with our previous observation that loss of DNAme allows PRC2 to deposit diffuse H3K27me3 (Van Mierlo et al., 2019; Walter et al., 2016).

To assay whether the chromatin remodeling at ERVKs following *Setdb1* KO results in their reactivation, we re-analyzed RNA-seq data generated from serum ESCs after *Setdb1* KO (Barral et al., 2022). Over 80 percent of ERVK elements show an increase in transcription after *Setdb1* KO (Supplemental Fig. 4B). SetDB1-targeted ERVKs that lost DNAme and H3K9me3 post-KO were upregulated by up to 3.3-fold. This indicates that the minor increase of H3K27me3 after SetDB1 depletion (Supplemental Fig. 4A) is insufficient to keep these retrotransposons repressed. Notably, upregulated ERVKs (> 1.5-fold increase of expression) include a large range of IAP, ETn, ERV-β2/4 and MERV elements (Supplemental Fig. 4C), all of which are capable of retrotransposition and pose a threat to genome integrity through insertional mutagenesis (Gagnier et al., 2019; Maksakova et al., 2006).

The presence of both DNAme and H3K9me3 is not exclusive to LTR-ERVKs, as this combination has also been observed at imprinted control regions (ICRs) (Fisher et al., 2017; Habibi et al., 2013; Leung et al., 2014), suggesting their potential dependency on SetDB1-mediated regulation. Indeed, 13 out of 17 (76%) ICRs described in the literature (Betto et al., 2021; Santini et al., 2021) are targeted by SetDB1, as indicated by 1-2 SetDB1 peaks within 1kb distance (Supplemental Fig. 4D). The DNAme level across the ICR averaged 47% and many ICRs were densely marked with H3K9me3 (Supplemental Fig. 5E). After *Setdb1* KO in serum ESCs, most ICRs lost H3K9me3 as well as proximal DNAme, the epigenetic mark that is essential for classical imprinting (Supplemental Fig. 4D-F; Supplemental Fig. 5F-G). Some ICRs were more susceptible to demethylation than others (Supplemental Fig. 4E), akin to specific ERVKs. Deposition of H3K27me3 slightly increased following *Setdb1* KO (except on the *Kcnq1ot1* and *Peg3* ICR), but this was insufficient to prevent the derepression of several imprinted genes (Supplemental Fig. 4D; italic names). Overall, these findings highlight a strong resemblance between SetDB1-mediated regulation of ICRs and ERVKs through a repressive DNAme-H3K9me3 axis in serum ESCs.

Our binning approach revealed that the SetDB1-targeted ΔDNAme↓ regions also include a small number of genic elements (Supplemental Fig. 3B). To examine which genes are regulated by the DNAme-mediated component of SetDB1, we investigated SetDB1-targeted genic loci. We observed only 37 demethylated target genes (ΔDNAme < -0.05) within 1kb proximity of at least one SetDB1 ChIP-seq peak. Similar to ERVKs and ICRs, demethylated target genes also lost their H3K9me3 enrichment after *Setdb1* KO (Supplemental Fig. 6A). Following *Setdb1* KO, 78 percent of demethylated target genes were increasingly expressed, up to 2.6 times (i.e. *Sult2a3*) (Supplemental Fig. 6B). GO-

enrichment analyses revealed enriched terms related to meiosis (Supplemental Fig. 6C), associated with *Btbd18*, *Rbm46* and *Tex12*, and consistent with previously reported silencing of meiosis- and gametogenesis-related genes by SetDB1 through DNAme-H3K9me3 (Karimi et al., 2011; Leung et al., 2014; Mochizuki et al., 2021; Wang et al., 2022). To find out whether nearby ERVKs are involved in the regulation of the demethylated target genes, we probed the number of demethylated ERVKs within 5kb proximity of these genes (Supplemental Fig. 6D). An enrichment for demethylated ERVKs was found near demethylated target genes, with more than two copies near demethylated SetDB1 target genes on average. Several of these ERVKs are embedded within regulatory elements of these genes, such as promoters, though the majority is found in introns (Supplemental Fig. 6E). For example, the derepressed *Zfp951* gene, that lost DNAme and H3K9me3 after *Setdb1* KO, embeds six demethylated ERVKs within or near its locus (Supplemental Fig. 6F). Hence, the DNAme-H3K9me3-mediated repression of most of these genes may originate from the targeting and regulation of flanking ERVKs by SetDB1. Notably, Karimi et al. (2011) reported that aberrant reactivation of ERVKs upon *Setdb1* KO is associated with disturbed expression of neighboring genes. Taken together, these observations suggest that ERVKs exert a DNAme- and H3K9me3-dependent repressive influence on nearby target genes, which is disrupted upon SetDB1 depletion.

Supplemental references

Barral, A., Pozo, G., Ducrot, L., Papadopoulos, G. L., Sauzet, S., Oldfield, A. J., ... & Déjardin, J. (2022). SETDB1/NSD-dependent H3K9me3/H3K36me3 dual heterochromatin maintains gene expression profiles by bookmarking poised enhancers. Molecular cell, 82(4), 816-832.

Betto, R. M., Diamante, L., Perrera, V., Audano, M., Rapelli, S., Lauria, A., ... & Martello, G. (2021). Metabolic control of DNA methylation in naive pluripotent cells. Nature genetics, 53(2), 215-229.

Fei, Q., Yang, X., Jiang, H., Wang, Q., Yu, Y., Yu, Y., ... & Shou, J. (2015). SETDB1 modulates PRC2 activity at developmental genes independently of H3K9 trimethylation in mouse ES cells. Genome research, 25(9), 1325-1335.

Fisher, C. L., Marks, H., Cho, L. T. Y., Andrews, R., Wormald, S., Carroll, T., ... & Skarnes, W. C. (2017). An efficient method for generation of bi-allelic null mutant mouse embryonic stem cells and its application for investigating epigenetic modifiers. Nucleic acids research, 45(21), e174-e174.

Gagnier, L., Belancio, V. P., & Mager, D. L. Mouse germ line mutations due to retrotransposon insertions. Mobile DNA. 2019; 10: 15. Epub 2019/04/24. doi: 10.1186/s13100-019-0157-4. PubMed PMID: 31011371.

Habibi, E., Brinkman, A. B., Arand, J., Kroeze, L. I., Kerstens, H. H., Matarese, F., ... & Stunnenberg, H. G. (2013). Whole-genome bisulfite sequencing of two distinct interconvertible DNA methylomes of mouse embryonic stem cells. Cell stem cell, 13(3), 360-369.

Karimi, M. M., Goyal, P., Maksakova, I. A., Bilenky, M., Leung, D., Tang, J. X., ... & Lorincz, M. C. (2011). DNA methylation and SETDB1/H3K9me3 regulate predominantly distinct sets of genes, retroelements, and chimeric transcripts in mESCs. Cell stem cell, 8(6), 676-687.

Leung, D., Du, T., Wagner, U., Xie, W., Lee, A. Y., Goyal, P., ... & Ren, B. (2014). Regulation of DNA methylation turnover at LTR retrotransposons and imprinted loci by the histone methyltransferase Setdb1. Proceedings of the National Academy of Sciences, 111(18), 6690-6695.

Liu, S., Brind’Amour, J., Karimi, M. M., Shirane, K., Bogutz, A., Lefebvre, L., ... & Lorincz, M. C. (2014). Setdb1 is required for germline development and silencing of H3K9me3-marked endogenous retroviruses in primordial germ cells. Genes & development, 28(18), 2041-2055.

Maksakova, I. A., Romanish, M. T., Gagnier, L., Dunn, C. A., Van de Lagemaat, L. N., & Mager, D. L. (2006). Retroviral elements and their hosts: insertional mutagenesis in the mouse germ line. PLoS genetics, 2(1), e2.

Mochizuki, K., Sharif, J., Shirane, K., Uranishi, K., Bogutz, A. B., Janssen, S. M., ... & Lorincz, M. C. (2021). Repression of germline genes by PRC1. 6 and SETDB1 in the early embryo precedes DNA methylation-mediated silencing. Nature communications, 12(1), 7020.

Prokopuk, L., Stringer, J. M., Hogg, K., Elgass, K. D., & Western, P. S. (2017). PRC2 is required for extensive reorganization of H3K27me3 during epigenetic reprogramming in mouse fetal germ cells. Epigenetics & chromatin, 10, 1-20.

Santini, L., Halbritter, F., Titz-Teixeira, F., Suzuki, T., Asami, M., Ma, X., ... & Leeb, M. (2021). Genomic imprinting in mouse blastocysts is predominantly associated with H3K27me3. Nature Communications, 12(1), 3804.

van Mierlo, G., Dirks, R. A., De Clerck, L., Brinkman, A. B., Huth, M., Kloet, S. L., ... & Marks, H. (2019). Integrative proteomic profiling reveals PRC2-dependent epigenetic crosstalk maintains ground-state pluripotency. Cell stem cell, 24(1), 123-137.

Walter, M., Teissandier, A., Pérez-Palacios, R., & Bourc'His, D. (2016). An epigenetic switch ensures transposon repression upon dynamic loss of DNA methylation in embryonic stem cells. elife, 5, e11418.

Wang, Z., Fan, R., Russo, A., Cernilogar, F. M., Nuber, A., Schirge, S., ... & Schotta, G. (2022). Dominant role of DNA methylation over H3K9me3 for IAP silencing in endoderm. Nature communications, 13(1), 5447.
