# Supplementary material for: Methylome profiling of SetDB1-deficient ESCs reveals coordinated epigenetic cross-talk during pluripotency
Source: Stem Cell Reports. 2026 Sep 3;21(10):103075. doi: 10.1016/j.stemcr.2026.103075 (PMC13681314; doi:10.1016/j.stemcr.2026.103075)
Supplement: Supplementary file 2 [file mmc3.pdf]

STEM CELL REPORTS
Article
OPEN ACCESS

Methylome profiling of SetDB1-deficient ESCs reveals coordinated epigenetic cross-talk during pluripotency

Nick G.P. Bovee,[1] Ehsan Habibi,[1] Zicong Liu,[1,9] Stefan H.A. Hoogland,[1,9] Siebren Frölich,[2] Daniëlle Diepenbroek,[1] Kim C.M. Bosman,[1] Julia Arand,[3] Irina A. Maksakova,[4] Anouk Klein Kranenbarg,[1] Jörn Walter,[5] Arie B. Brinkman,[1] Klaas W. Mulder,[2] Joop H. Jansen,[6] Michiel Vermeulen,[7,8] Matthew Lorincz,[4] Hendrik G. Stunnenberg,[1] and Hendrik Marks[1,10,*]

[1]Department of Molecular Biology, Faculty of Science, Radboud Institute for Molecular Life Sciences (RIMLS), Radboud University, Nijmegen, the Netherlands
[2]Department of Molecular Developmental Biology, Faculty of Science, Radboud Institute for Molecular Life Sciences (RIMLS), Radboud University, Nijmegen, the Netherlands
[3]Center for Anatomy and Cell Biology, Division of Cell and Developmental Biology, Medical University of Vienna, Vienna, Austria
[4]Department of Medical Genetics, Life Sciences Institute, University of British Columbia, Vancouver, BC, Canada
[5]Department of Life Sciences, Laboratory of Genetics, Saarland University, Saarbrücken, Germany
[6]Department of Laboratory Medicine, Laboratory of Hematology, Radboud University Medical Center and Radboud Institute for Molecular Life Sciences (RIMLS), Nijmegen, the Netherlands
[7]Department of Molecular Biology, Faculty of Science, Radboud Institute for Molecular Life Sciences (RIMLS), Oncode Institute, Radboud University, Nijmegen, the Netherlands
[8]Division of Molecular Genetics, Netherlands Cancer Institute, Amsterdam, the Netherlands
[9]These authors contributed equally
[10]Lead contact
*Correspondence: hendrik.marks@ru.nl
https://doi.org/10.1016/j.stemcr.2026.103075

SUMMARY

SetDB1 is best known for catalyzing H3K9me3, but it also influences H3K27me3 deposition, CTCF-binding, and DNA methylation (DNAme). Given the interplay between DNAme and the other epigenetic features, we profiled DNAme following *Setdb1* knockout (KO) in ground-state and serum-grown mouse embryonic stem cells (ESCs) to illuminate DNAme-dependent and -independent functions of SetDB1. Time-course whole-genome bisulfite sequencing of serum-grown ESCs shows that nearly half of SetDB1 binding sites are enriched with DNAme and H3K9me3, primarily at retrotransposons. Upon *Setdb1* KO, both H3K9me3 and DNAme are reduced, with DNAme rapidly removed at many sites by TET enzymes. Some retrotransposons, primarily IAPs, are TET-resistant and lose DNAme slowly via passive dilution. Notably, SetDB1-mediated regulation of H3K27me3, CTCF-binding, and SMAD3 are uncoupled from the DNAme-H3K9me3 axis, and from each other. AlphaFold modeling and co-immunoprecipitation mass spectrometry suggest this uncoupling involves competitive binding to distinct SetDB1 protein domains, highlighting the complex coordination underlying SetDB1 functions.

INTRODUCTION

Chromatin regulation is essential during mammalian development, controlling gene expression to establish proper cell identities and lineage decisions. In pluripotent mouse embryonic stem cells (ESCs), the chromatin regulator SetDB1 targets up to 20,000 genomic loci (Bilodeau et al., 2009; Karimi et al., 2011; Yuan et al., 2009) and controls various cellular processes, including maintenance of the pluripotent state, differentiation, and metabolism (Bilodeau et al., 2009). As such, depletion of SetDB1 is lethal during embryonic implantation (Dodge et al., 2004). SetDB1 mainly functions as a histone methyltransferase that catalyzes trimethylation of lysine 9 on histone 3 (H3K9me3) (Markouli et al., 2021), a hallmark of heterochromatin. However, despite widespread deposition of this mark, depletion of SetDB1 results in derepression of only 33 genes that lose H3K9me3 in ESCs (Karimi et al., 2011), suggesting that SetDB1 mainly facilitates H3K9me3-mediated silencing at non-genic regions (Matsui et al., 2010; Leung and Lorincz, 2012; Karimi et al., 2011).

Further complicating the characterization of SetDB1 function, nearly half (46%) of its binding sites in ESCs are not enriched for H3K9me3 (Fei et al., 2015). While SetDB1 was reported to interact with PRC2 subunits to modulate H3K27me3 deposition, this appeared to be restricted to ∼5% of SetDB1 target sites, as shown by the limited overlap between genomic loci bound by SetDB1, H3K27me3, and/or PRC2 (Fei et al., 2015). More recently, SetDB1 was shown to associate with CTCF and Cohesin subunits in ESCs (Warrier et al., 2022). However, colocalization of SetDB1 and CTCF was also observed in only ∼14% of SetDB1 binding sites.

The protein domain composition of SetDB1 suggests the possibility of an additional regulatory mechanism beyond regulation of H3K9me3, H3K27me3, and CTCF-binding. SetDB1 incorporates a methyl-CpG-binding domain (MBD) with two arginine residues that enable DNA binding (Markouli et al., 2021). This MBD, however, primarily

© 2026 The Author(s).
Published by Elsevier Inc. on behalf of International Society for Stem Cell Research.
This is an open access article under the CC BY license (http://creativecommons.org/licenses/by/4.0/).

facilitates protein-protein interactions, including with DNA methyltransferases (DNMTs), which catalyze DNA methylation (DNAme) (Li et al., 2006; Markouli et al., 2021), another major heterochromatin mark. Li et al. (2006) demonstrated that SetDB1 directly interacts with *de novo* DNMT3 enzymes. Although SetDB1 has been implicated in maintaining DNAme at specific retrotransposons and germline promoters, how loss of SetDB1 impacts DNAme dynamics over time has not been addressed.

Moreover, a comprehensive integration of SetDB1's functional mechanisms and their mutual interplay is currently lacking. Given the fact that DNA regions showing SetDB1-dependent H3K9me3 are frequently methylated in serum ESCs, cross-talk with the DNAme machinery may be particularly important (Karimi et al., 2011; Leung et al., 2014; Matsui et al., 2010). In addition, H3K27me3 and DNAme are generally mutually exclusive (Brinkman et al., 2012; van Mierlo et al., 2019), and CTCF-binding is impaired by DNAme (Monteagudo-Sánchez et al., 2024). Given this cross-regulatory role, DNAme could be the missing link that connects other SetDB1-associated regulatory functions, thereby illuminating which functions are coupled or uncoupled. The SetDB1 interactions and associated protein domains underlying these potential connections also remain largely unexplored, and warrant a systematic investigation.

Therefore, we set out to comprehensively characterize the DNAme-mediated regulatory function of SetDB1 in pluripotent mouse ESCs through whole-genome bisulfite sequencing-based methylome time-course analyses following *Setdb1* knockout (KO). These analyses include hypermethylated serum-grown ESCs, as well as hypomethylated 2i-grown pluripotent ground-state ESCs. By integrating our temporally resolved methylome profiles with chromatin proteomic profiling following *Setdb1* KO, RNA-sequencing (RNA-seq) expression data, and DNA-binding profiles of SetDB1, H3K9me3, H3K27me3, TET1, TET2, and CTCF, our analyses define distinct modes of action of SetDB1 and their coordinated interplay with DNAme across diverse genomic elements. To further elucidate how direct interactions with SetDB1 could facilitate these distinct modes, we performed interaction modeling using AlphaFold in combination with SetDB1 co-immunoprecipitation (coIP) followed by mass spectrometry (MS). Altogether, these analyses provide a comprehensive view of SetDB1 function in mouse pluripotency.

RESULTS

Methylome profiling upon loss of SetDB1 reveals two distinct modes of action

To investigate the regulation of DNAme by SetDB1 during pluripotency, we made use of *Setdb1*$^{CKO/-}$ conditional KO mouse ESCs, in which SetDB1 deletion can be induced by addition of 4-hydroxytamoxifen (4-OHT) (Matsui et al., 2010) (Figures 1A and S1A). Four days of 4-OHT treatment induced exon deletion in *Setdb1* (Figure S1B), leading to persistent loss of SetDB1 protein, as shown by label-free quantitative chromatin-enrichment for proteomics followed by MS antitative (ChEP-MS) (Figure S1C). To provide a global overview of DNAme levels after SetDB1 depletion, we quantified DNAme through MS at multiple time points following *Setdb1* KO in ESCs cultured in medium containing serum and LIF (abbreviated as "serum ESCs") (Figure S1D). Similar to SetDB1-deficient primordial germ cells (PGCs) (Liu et al., 2014), DNAme increased substantially after SetDB1 depletion in serum ESCs, rising from day 4 and peaking around day 10. We confirmed this trend in a second, independent *Setdb1*$^{CKO/-}$ line (Fisher et al., 2017) (Figure S1E). Prolonged culturing of SetDB1-deficient serum ESCs resulted in growth arrest (Figures S1D and S1F) (Matsui et al., 2010) and cell death by day 15 (Figures 1A and S1D). Consistently, ChEP-MS revealed the progressive accumulation of proteins involved in programmed cell death, loss of differentiation repressors (Figure S1G), and a collapse of the pluripotency network (Figure S1H).

Next, we performed whole-genome bisulfite sequencing (WGBS) to resolve DNAme dynamics at base-pair resolution. In line with our MS data, SetDB1 depletion in serum ESCs resulted in a global increase in DNAme across CpGs (Figures 1B and S2A). This was reflected by a noticeable shift from low to moderately methylated CpGs (0–0.75) toward highly methylated CpGs (0.80–1.00), with many moderately methylated CpGs becoming hypermethylated by day 10 (Figure S2B). This expansion was genome-wide, affecting CpGs across all chromosomes (Figure S2C). Consistent with the genome-wide increase in DNAme, ChEP-MS showed increased chromatin association of *de novo* DNMT3 enzymes, as well as the maintenance enzyme DNMT1, immediately after SetDB1 depletion (Figure 1C). The elevated recruitment of DNMTs decreased by day 10, coinciding with reduced cell proliferation (Figures S1D and S2D).

To characterize differentially methylated regions in SetDB1-deficient serum ESCs and uncover the DNAme-mediated regulatory mechanisms of SetDB1, we divided the methylome into 10 kb bins. Ranking these bins by their average methylation change on day 10 revealed that most bins gained DNAme after *Setdb1* KO (Figure 1D). To obtain a robust subset of bins with ΔDNAme↑, we filtered out bins with minimal deviation from the global trend, yielding 73% of bins with increased DNAme. A smaller subset showed decreased methylation (3.3%; Figure 1D), which we defined as ΔDNAme↓ subset. Notably, the demethylated ΔDNAme↓ bins had the highest endogenous DNAme levels in serum ESCs (Figure 1E), suggesting a significant divergence from their default chromatin state after *Setdb1* KO.

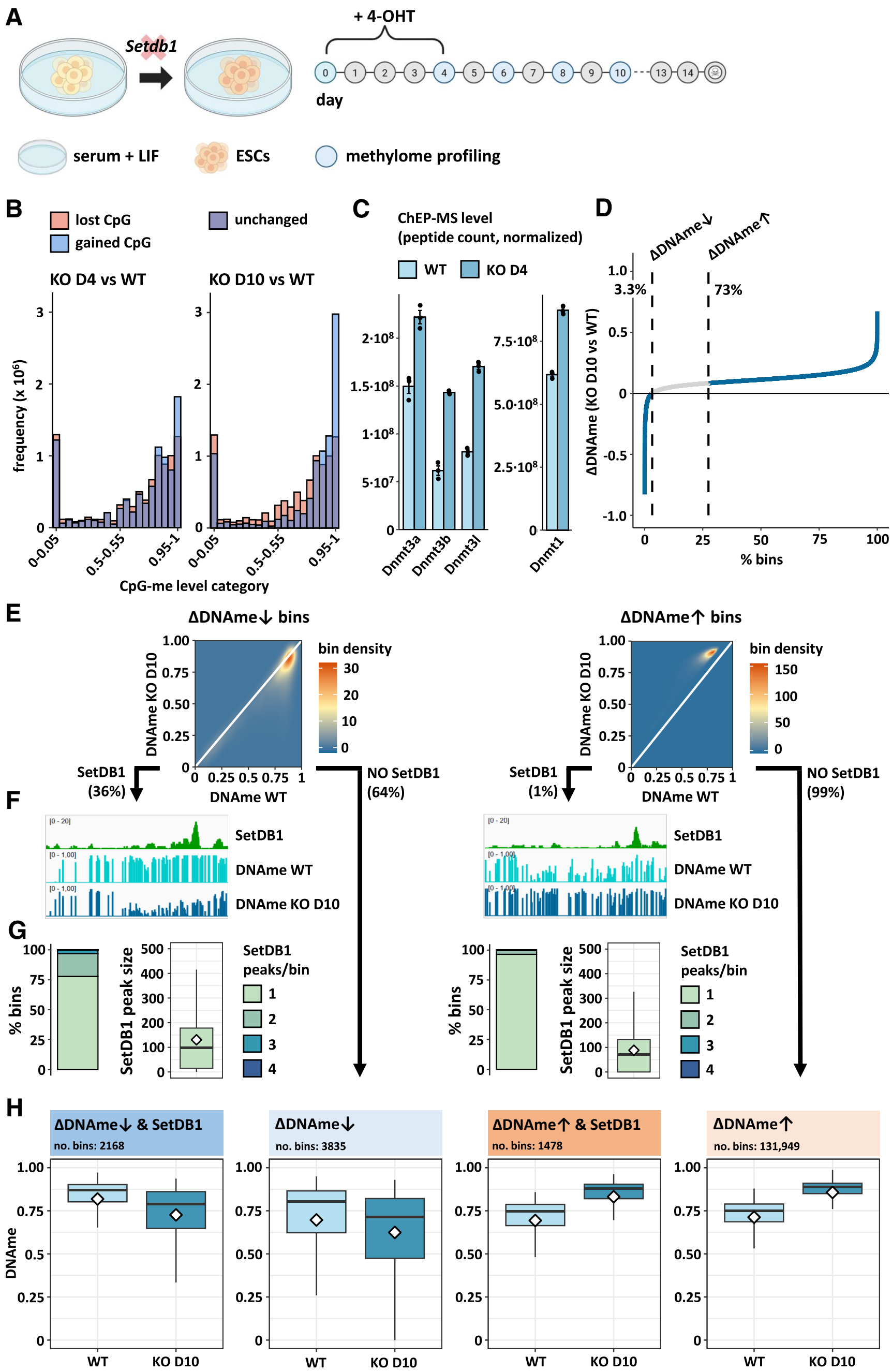

Figure 1. Methylome profiling in serum ESCs upon SetDB1 loss reveals interplay between SetDB1 and DNA methylation

(A) Schematic of workflow for methylome profiling upon induction of *Setdb1* KO in serum ESCs.

(B) Bar graph of differential CpG methylation upon induction of *Setdb1* KO in serum ESCs. CpGs are categorized in aggregated methylation levels with 0.05 step size and sorted from lowest to highest.

(C) Bar graph showing normalized peptide counts (DIA-NN) of DNMT enzymes in WT serum ESCs, and at day 4 post-*Setdb1* KO induction, as measured by ChEP-MS. Dots represent individual values of replicates (n = 3). Error bars indicate standard error from mean.

(D) Line graph illustrating methylome remodeling of 10 kb genomic bins at 10 days post-*Setdb1*-KO induction in serum ESCs, with bins sorted by their average methylation change. Vertical dashed lines represent thresholds for ΔDNAme↓ and ΔDNAme↑ subsets, with left dashed line representing ΔDNAme < 0 and right dashed line representing 25th quantile of ΔDNAme > 0.

(E) Heatmaps showing methylation remodeling of bins categorized in (D) (6,003 ΔDNAme↓ and 133,427 ΔDNAme↑ bins). Bin count density on color scale. Diagonal white lines represent slope = 1.

(F) IGV screenshots of SetDB1 ChIP-seq and WGBS data of exemplary ΔDNAme↓ and ΔDNAme↑ bins with SetDB1 binding.

(G) Integration of previously defined bins in (D) with nearby SetDB1 binding. SetDB1 ChIP-seq peaks are allocated to ΔDNAme↓ bins and ΔDNAme↑ bins if within 1 kb distance. Bar plot displays number of identified peaks near both bin categories. Boxplots represent a quantification of individual peak sizes (TMM-normalized values). White diamonds in boxplots depict mean TMM.

(H) Boxplots of DNAme in WT and *Setdb1* KO serum ESCs on day 10 at bins defined in (D) and (E), further categorized by the presence or absence of SetDB1 peaks as defined in (F). White diamonds represent averages.

To distinguish between direct and indirect effects of SetDB1 depletion on the methylome, we overlapped our methylome bins with SetDB1 chromatin immunoprecipitation sequencing (ChIP-seq) profiles (Warrier et al., 2022). SetDB1 peaks were detected within 1 kb of both ΔDNAme↑ and ΔDNAme↓ bins, with similar peak sizes (Figures 1F and 1G). However, demethylated bins were bound much more frequently by SetDB1 (36%) than bins that gained methylation (1%). CpG-level analysis showed the same trend, with stronger demethylation after KO correlating with closer proximity of SetDB1 binding (Figure S2E). These results indicate that local demethylation was likely a direct result of loss of proximal SetDB1 binding. In contrast, SetDB1-targeted ΔDNAme↑ bins

had low endogenous DNAme levels, and gained DNAme after KO similarly to SetDB1-unbound bins (Figure 1H), suggesting an indirect effect. Together, our findings suggest that SetDB1 operates through two distinct modes of action: (1) SetDB1 binds specific targets and guides the deposition of DNAme, and (2) SetDB1 binding does not influence DNAme, and loci instead exhibit global, SetDB1-independent DNAme increases after KO.

Loss of SetDB1-dependent DNAme is coupled to H3K9me3 loss at ERVKs and driven by pre-loaded TETs

To determine whether regions that gained or lost DNAme following SetDB1 depletion are enriched for H3K9me3 or H3K27me3, we integrated the methylome bins with previously generated H3K9me3 (Fisher et al., 2017) and H3K27me3 ChIP-seq profiles (Fei et al., 2015), both generated from WT and *Setdb1* KO serum ESCs. Since these repressive histone marks cover large genomic regions (Chadwick and Willard, 2004; Pauler et al., 2009; Squazzo et al., 2006), we analyzed changes of these marks across entire methylome bins. This revealed that ΔDNAme↑ bins were not enriched for H3K9me3 under WT conditions, regardless of SetDB1 presence (Figures 1H and 2A: orange categories). In contrast, large H3K9me3 enrichments were almost exclusively found on the ΔDNAme↓ SetDB1-bound bins where DNAme was also elevated before *Setdb1* KO (Figures 1H and 2A: dark blue category). In addition to a reduction in DNAme after KO within these bins, H3K9me3 was reduced to levels comparable to those of other bins. These results indicate that SetDB1-dependent DNAme is closely linked to H3K9me3 deposition. Conversely, H3K27me3 levels changed only modestly across all bin categories after *Setdb1* KO (Figure S3A).

Next, we investigated what genomic features are associated with SetDB1 targets that lose H3K9me3 and DNAme after SetDB1 depletion. SetDB1-targeted ΔDNAme↓ bins were primarily located in distal intergenic regions, whereas SetDB1-bound ΔDNAme↑ bins were relatively enriched with genic features, especially intronic elements (Figure S3B). This suggests SetDB1-dependent H3K9me3 and DNAme are associated with non-coding and repetitive elements, consistent with established roles of SetDB1 (Karimi et al., 2011; Matsui et al., 2010). To refine our analysis, we quantified the overlaps of bins with repetitive elements and compared them with expected frequencies (Figure S3C). The SetDB1-targeted ΔDNAme↓ bins are highly enriched for LTR retrotransposons, particularly ERVK elements, with the highest enrichment observed for IAPEz internal regions (IAPEz-int: 17.9× enrichment) (Figure S3D). This aligns with previous studies identifying SetDB1-dependent H3K9me3 and DNAme as key regulators of IAPEz elements in ESCs (Karimi et al., 2011; Leung et al., 2014), PGCs (Liu et al., 2014), and visceral endoderm cells (Wang et al., 2022). Other enriched ERVK elements in SetDB1-targeted ΔDNAme↓ bins included other IAPs and their flanking LTRs (9.6× enrichment), MMERVKs (7.4×), and RLTRs (4.5×) (Figure S3D). In contrast, SetDB1-targeted ΔDNAme↑ bins are not enriched for LTRs or any other repetitive element (Figure S3C).

Consistent with our observation that SetDB1-dependent H3K9me3-marked bins lose DNAme after KO, Leung et al., (2014) proposed that the SetDB1-H3K9me3 axis protects against demethylation. However, loss of H3K9me3 and DNAme seem to occur with distinct dynamics: SetDB1-dependent H3K9me3 is largely lost in 2–4 days after KO (Fisher et al., 2017; Matsui et al., 2010), whereas DNAme changes peak around day 10 (Figure 1B). To examine their temporal relationship, we used our WGBS time-course to track DNAme dynamics at SetDB1-targeted ΔDNAme↓ bins following SetDB1 depletion. Ward-D clustering identified three clusters within SetDB1-targeted ΔDNAme↓ bins with distinct demethylation trajectories. The largest group, cluster 1, exhibited minor and slow demethylation, and retained relatively high DNAme levels throughout the time-course (Figures 2B and 2C). This pattern is consistent with passive DNAme dilution, potentially resulting from impaired DNMT3 and/or DNMT1 recruitment to H3K9me3-marked regions (Fang et al., 2016). In contrast, clusters 2 and 3 underwent rapid and extensive demethylation immediately after SetDB1 depletion, differing primarily in their initial and residual DNAme levels after KO (Figures 2B and 2C).

To determine whether these demethylation dynamics depend on H3K9me3, we compared H3K9me3 levels across the three clusters of SetDB1-targeted ΔDNAme↓ bins (Figure 2D). Although cluster 1 exhibited slightly higher initial H3K9me3 levels, all clusters lost H3K9me3 by day 6 of *Setdb1* KO. Given the pronounced and continued demethylation of cluster 1 bins between days 8 and 10, the presence of H3K9me3 does not appear to be the main factor preventing excessive demethylation. H3K27me3 also appeared to have little influence on DNAme loss, as H3K27me3 levels were low among most SetDB1-targeted ΔDNAme↓ bins (Figure S3A), with only a modest enrichment in cluster 3 (Figure 2D), potentially due to lower DNAme overall (Figure 2C). Moreover, H3K27me3 levels remained largely unchanged after 3 days of *Setdb1* KO, regardless of clear alterations in DNAme (e.g., Figures S1D and S1E) and H3K9me3 (Fisher et al., 2017; Matsui et al., 2010). Despite a slight misalignment of time points between the integrated datasets, we conclude that demethylation dynamics following SetDB1 loss in ESCs mainly depend on chromatin features other than H3K9me3 and H3K27me3.

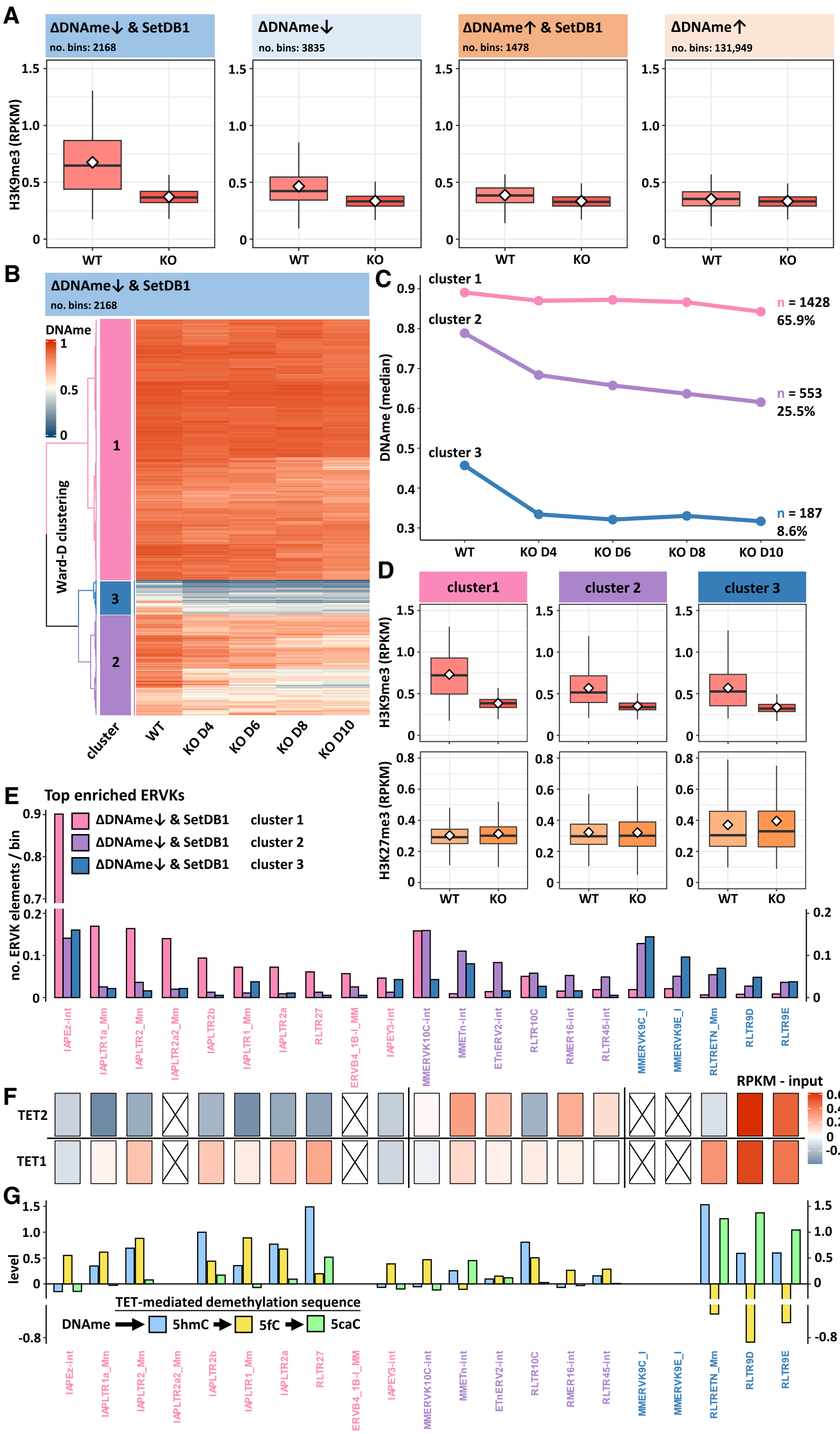

Figure 2. Loss of SetDB1-dependent DNAme is coupled to H3K9me3 loss at ERVKs in serum ESCs, but demethylation speed is driven by pre-loaded TET1/2

(A) Boxplots of H3K9me3 levels over the entirety of methylome bins, as defined in Figure 1H, in WT and *Setdb1* KO serum ESCs. White diamonds represent averages.

(B) Heatmap of DNAme levels at SetDB1-targeted ΔDNAme↓ bins in WT and *Setdb1* KO serum ESCs. Bins were clustered by Ward-D method into three distinct clusters.

(C) Line graph showing the DNAme remodeling dynamics of clustered bins, as identified in (B). Dots represent median DNAme levels over bin categories.

(D) Boxplots of H3K9me3 and H3K27me3 levels over entirety of bin clusters selected from (B), as measured in WT and *Setdb1* KO serum ESCs. White diamonds represent averages.

(E) Bar graph showing the observation frequency of the 10 most-occurring ERVKs in ΔDNAme↓ SetDB1-target bin clusters from (B). Some ERVK species appeared in the top 10 of multiple clusters and were colored according to the cluster in which they were most frequently observed.

(F) Heatmap showing TET1 and TET2 targeting in WT serum ESCs of ERVKs listed in (E). Values represent RPKM values of ChIP-seq profiles subtracted with input ChIP-seq values. Black cross indicates that ChIP-seq reads could not be aligned to specific ERVK elements.

(G) Bar graph showing levels of TET-mediated oxidation products on ERVKs categorized in (E) and (F), mined from genome-wide distribution maps of 5hmC/5fC/5caC generated with modification-specific antibodies. Levels reflect comparative measurements between TDG-deficient serum ESCs and WT ESCs.

Interestingly, we noticed that IAPs were mostly located in the slowly demethylated bins from cluster 1 (Figure 2E). Hairpin bisulfite sequencing confirmed that IAPs undergo only minor demethylation following *Setdb1* KO (Figure S3E). In contrast, MMERVKs, (MM-)ETn elements and their flanking RLTRs were primarily found in the rapidly demethylated bins from clusters 2 and 3 (Figure 2E). As the TET family of proteins are potent

DNA demethylation enzymes (Deniz et al., 2018; Lio et al., 2020; de la Rica et al., 2016), we hypothesized that bins from clusters 2 and 3 lose DNAme quickly through a combination of passive dilution and active removal by TETs (Leung et al., 2014). Given that the abundance of TET enzymes is stable after *Setdb1* KO, as shown by our ChEP-MS (Figure S2D), TETs may already be pre-loaded at rapidly demethylated ERVKs. To test this hypothesis, we mapped TET1 and TET2 ChIP-seq profiles from serum ESCs (Xiong et al., 2016; Williams et al., 2011) to the ERVK elements associated with different clusters (Figure 2F). This indicated that IAP elements that were assigned to cluster 1 lacked TET2 pre-loading. The internal regions of these IAPs (termed "-int") were also devoid of TET1 pre-loading, while their flanking LTRs (termed IAPLTR), which contain the regulatory regions of the provirus, exhibited moderate TET1 enrichment. The ERVKs from cluster 2 displayed comparatively higher TET2 pre-loading. Meanwhile, MMERVK9s from cluster 3 showed the lowest initial DNAme levels and rapid demethylation, and had the greatest pre-loading of both TET1 and TET2. Notably, the greatest pre-loading of TETs was detected in LTR regions (i.e., RLTR9D and RLTR9E), but not within internal regions of proviruses (i.e., MMERVK9C_I and MMERVK9E_1).

To evaluate the enzymatic activity of these pre-loaded TETs, we mined profiles of TET-mediated oxidation products (5hmC, 5fC, and 5caC) upon *Tdg* knockdown in serum ESCs (Shen et al., 2013). Depletion of TDG leads to the accumulation of TET-mediated oxidation products by blocking the final step of active demethylation. Following *Tdg* knockdown, cluster 3 ERVKs showed increased 5caC levels, the final TET-mediated oxidation product, indicating active DNAme turnover (Figure 2G). In contrast, cluster 2 ERVKs, which maintained higher overall DNAme compared to cluster 3 ERVKs, mostly accumulated 5fC, and the slowly demethylated IAP elements from cluster 1 accumulated 5fC and/or 5hmC. Together, our results show that specific ERVK subfamilies are pre-loaded with TET enzymes, especially at their LTR regions, and display active DNAme turnover. Under WT conditions, the active DNAme turnover is counterbalanced by SetDB1-mediated DNAme deposition. After *Setdb1* KO, this balance is disrupted, leading to rapid demethylation by pre-loaded TETs. Specific elements, particularly IAPEz and IAPEY elements, appear shielded from TET pre-loading, especially TET2, and active demethylation. Our results indicate that factors other than H3K9me3 (and H3K27me3) protect against such rapid and excessive demethylation by pre-loaded TETs. Protection of IAP elements against rapid demethylation may be particularly important, as IAPs are still actively retrotransposing in the rodent lineage and are in turn highly mutagenic in both germline and somatic tissues (Maksakova et al., 2006). Consistent with this notion, the direct analysis of individual ERVK loci, including IAPs, revealed that reduced H3K9me3 and DNAme after SetDB1 loss lead to transcriptional derepression of these elements, similar to SetDB1-targeted imprinted control regions (ICRs) (Note S1; Figures S4–S6).

DNAme-H3K9me3-and H3K27me3-mediated regulatory mechanisms of SetDB1 are uncoupled

A substantial fraction of SetDB1-targeted bins does not contain SetDB1-dependent DNAme and H3K9me3, and displays DNAme increases following *Setdb1* KO that mirror the global, non-specific trend (Figures 1 and 2: "ΔDNAme↑ & SetDB1" bin category). Here, we examine what regulatory roles SetDB1 has at these sites. Fei et al. (2015) demonstrated that a subset of SetDB1 targets are not enriched for H3K9me3, but overlap with H3K27me3 enrichments instead, regulating nearby neural development genes. Therefore, we asked whether SetDB1 targets without SetDB1-dependent DNAme represent these H3K27me3-marked loci. To enable direct comparison with our data, we conducted a SetDB1 peak-centered analysis. Thereto, we ranked all 10 kb regions surrounding a SetDB1 peak summit, similar to Fei et al. (2015), based on their DNAme changes after *Setdb1* KO. We selected fractions above the 25th percentiles of demethylated and increasingly methylated groups to obtain subsets with and without SetDB1-dependent DNAme, respectively (Figure 3A). Of note, this peak centered analysis confirmed our previous observations of a tightly coupled DNAme-H3K9me3 mechanism by SetDB1 (Figures 3A, S7A, and S7B).

Plotting the H3K27me3 profiles (Fei et al., 2015) over all SetDB1 peak regions confirmed that approximately 5% of SetDB1 targets are endogenously enriched with H3K27me3 (Fei et al., 2015) (Figure S7C). Remapping the H3K27me3 enrichments to the ΔDNAme-ranking of SetDB1 peak regions (Figure S7D) revealed that most H3K27me3 enrichments were located on SetDB1 targets without SetDB1-dependent DNAme and H3K9me3 (Figures S7A, S7B, and S7E: ΔDNAme↑ column). As such, almost all of these H3K27me3-decorated regions do not overlap with the SetDB1-targeted ΔDNAme↓ bins from Figures 1 and 2. Notably, only 22.8% of H3K27me3-enriched SetDB1 targets (equivalent to 1.14% of total SetDB1 targets) experienced a loss of 10% or more of H3K27me3 following *Setdb1* KO (Figures S7C–S7E: dark purple category), representing targets regulated through SetDB1-mediated H3K27me3. These SetDB1-mediated H3K27me3 targets are almost entirely confined to ΔDNAme↑ SetDB1 peak regions (Figures S7D and S7E) and show relatively strong localization toward promoter

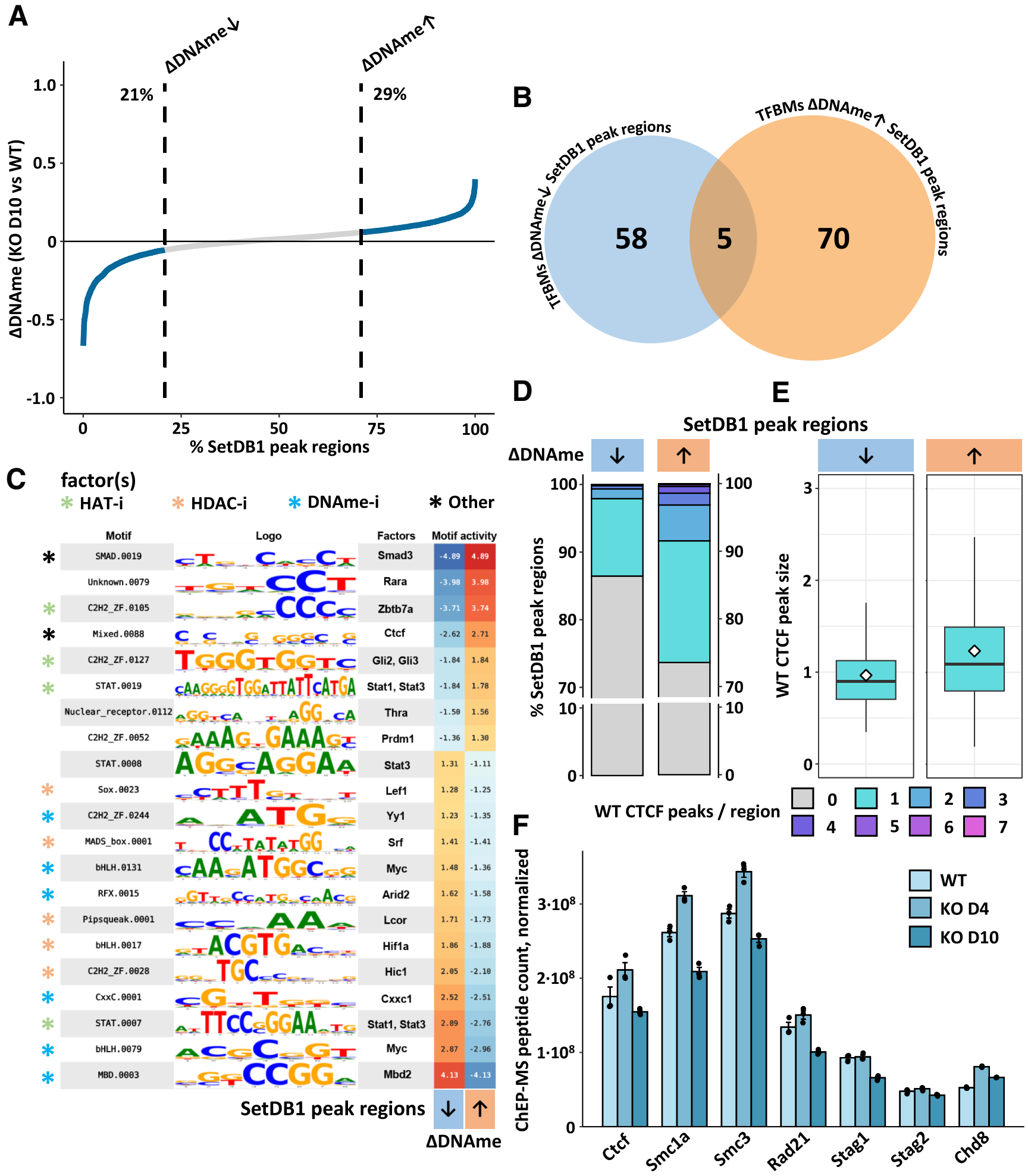

Figure 3. Targets without SetDB1-mediated repression are enriched with binding motifs of SMAD3 and CTCF

(A) Line graph illustrating methylome remodeling of SetDB1 peak regions (10 kb centered on ChIP-seq peak summits) at 10 days post-*Setdb1* KO induction in serum ESCs. Vertical dashed lines indicate 25th quantiles of ΔDNAme < 0 and ΔDNAme > 0 peak regions, which we used to define robust categories of ΔDNAme↓ and ΔDNAme↑ SetDB1 targets, respectively.

(B) Venn diagram showing overlap between TFBMs that are enriched (according to Gimme Maelstrom) in SetDB1 binding sites (200 bp centered on ChIP-seq peak summit) within categories defined in (A).

(C) Schematic representation that highlights selection of enriched TFBMs in SetDB1 peak region categories as defined in (A).

(D) Bar graph showing number of CTCF ChIP-seq peaks from WT serum ESCs near SetDB1 peak regions (within 1 kb distance), as categorized in (A).

(E) Boxplots showing sizes of individual CTCF peaks in WT serum ESCs, as read pileups normalized to 10 bp, calculated by Macs2, near SetDB1 peak region categories from (A). White diamonds represent averages.

(F) Bar graph showing normalized peptide counts (DIA-NN) of CTCF, and of CTCF-associated proteins in WT serum ESCs, and at days 4 and 10 post-*Setdb1* KO induction, as measured by ChEP-MS. Dots represent individual values of replicates ($n = 3$). Errors bars indicate standard error from mean.

and exonic regions of genes (Figure S7F). Together, these findings indicate that the H3K27me3- and DNAme-H3K9me3-mediated regulatory mechanisms of SetDB1 are uncoupled.

SetDB1 targets without SetDB1-dependent DNAme are enriched with TF motifs that allow regulation beyond heterochromatin formation

Considering the limited extent of SetDB1-mediated H3K27me3 regulation among SetDB1 targets without SetDB1-mediated DNAme, we examined what other SetDB1 regulatory mechanisms might be operating at these sites. Thereto, we analyzed transcription factor (TF) binding motifs (TFBMs) among SetDB1 binding sites. We extracted the nucleotide sequences of SetDB1 binding sites (i.e., 200 bp surrounding a peak summit) from peak regions that showed ΔDNAme↑ or ΔDNAme↓ post-KO (Figure 3A), and identified enriched TFBMs using Gimme Maelstrom (Bruse and Heeringen, 2018). We identified 75 enriched TFBMs within ΔDNAme↑ SetDB1 binding sites, 70 of which were not enriched among SetDB1 ΔDNAme↓ binding sites, and 58 motifs unique to ΔDNAme↓ binding sites (Figure 3B; Table S1).

As expected, TFBMs associated with ΔDNAme↓ SetDB1 targets were linked to heterochromatic features, such as DNAme and histone deacetylation. Conversely, TFBMs of ΔDNAme↑ SetDB1 targets were linked to histone acetyltransferase activity and transcriptional activation (Figures 3C and S8A), indicative of SetDB1 recruitment to non-heterochromatic regions. We also observed a strong enrichment of SMAD3 motifs here (Figure 3C), which is the primary mediator of TGF-β signaling. Notably, our ChEP-MS showed increased recruitment of proteins involved in SMAD signaling and TGF-β production after *Setdb1* KO (Figure S1G), together suggesting that SetDB1 may limit these activities in WT serum ESCs, uncoupled from its DNAme-H3K9me3 regulatory mechanism.

The SMAD3-SetDB1 axis was previously implicated in epithelial-mesenchymal transition (EMT) in disease states (Du et al., 2018; Liu et al., 2022), hence the SMAD3-SetDB1 link identified here might be associated with the EMT during embryonic gastrulation.

The co-occurrence of SetDB1-binding and CTCF motifs (Figure 3C) suggests that SetDB1 may also contribute to large-scale 3D chromatin organization (Tam et al., 2024). Interestingly, CTCF-binding is known to be highly sensitive to DNAme levels at its binding sites, with DNAme generally impairing CTCF-binding and chromatin looping (Monteagudo-Sánchez et al., 2024). This aligns with our observation that CTCF motifs are particularly enriched among SetDB1 targets that contain relatively low DNAme under WT conditions (Figure S7A: ΔDNAme↑ category; cf. ΔDNAme↓ category). This observation parallels the results of Tam et al. (2024), who reported that SetDB1-dependent H3K9me3—tightly coupled to DNAme in our data—and CTCF are mutually exclusive.

To substantiate the enrichment of CTCF motifs among ΔDNAme↑ SetDB1 targets, we overlayed the SetDB1 peak regions with CTCF ChIP-seq profiles of WT and *Setdb1* KO serum ESCs (Tam et al., 2024). This revealed that 2.7 times more ΔDNAme↑ SetDB1 targets overlapped with CTCF than ΔDNAme↓ counterparts (Figure 3D; 619/2340 = 26% compared to 227/1676 = 13%). Endogenous CTCF peaks among ΔDNAme↑ SetDB1 peak regions were also slightly larger on average, and contained more pronounced outliers (Figure 3E), consistent with the inhibiting effect of DNAme on CTCF-binding (Monteagudo-Sánchez et al., 2024). Following SetDB1 loss, both ΔDNAme↓ and ΔDNAme↑ SetDB1 peak regions gained CTCF-binding (Figure S8B). This is reflected by a global increase of CTCF, SMC1a, and SMC3 proteins to chromatin immediately after *Setdb1* KO (Figure 3F). Notably, CTCF peak sizes especially increased at ΔDNAme↓ SetDB1 targets after KO (Figure S8C), potentially due to the removal of DNAme that endogenously obstructed CTCF-binding at these sites. Likewise, SetDB1 targets that lacked endogenous CTCF and underwent demethylation after *Setdb1* KO, gained the most CTCF peaks among all SetDB1 targets post-KO (Figure S8B).

To assess whether CTCF modulation by SetDB1 operates independently of its H3K27me3 regulatory mechanism, we assayed the number of CTCF peaks before and after *Setdb1* KO at H3K27me3-mediated targets. Most of these targets were not endogenously bound by CTCF (Figure S8D), and did not acquire CTCF-binding after KO. Although more than a quarter of H3K27me3-mediated SetDB1 targets was natively associated with CTCF, CTCF peak numbers remained unchanged here after SetDB1 loss, suggesting that regulation of CTCF occurred independently of SetDB1. Collectively, our analyses revealed two uncoupled repressive mechanisms of SetDB1. Most repressed targets are regulated through a DNAme-H3K9me3 dual silencing mechanism, whereas a minor subset is regulated through H3K27me3. In addition, SetDB1 modulates CTCF-binding at a subset of targets independently of its repressive functions. SetDB1 targets that lack DNAme-H3K9me3 are also highly enriched for SMAD3 and other TF motifs, highlighting a broader role of SetDB1 in genome regulation beyond heterochromatin formation.

Loss of SetDB1-dependent DNAme reflects repressive buffering deficit in ground-state 2i ESCs

The tight connection between SetDB1-mediated H3K9me3 and DNAme raises the question what regulatory role DNAme fulfills at these targets. While serum ESCs exhibit a hypermethylated metastable state, the hypomethylated ground-state of pluripotency, characterized by the absence of DNMT3 enzymes, can be modeled *in vitro* by ESCs cultured in the presence of two inhibitors for MEK and GSK-3, along with LIF (referred to as “2i ESCs”) (Ficz et al., 2013; Habibi et al., 2013; Leitch et al., 2013). Therefore, we assessed the functional importance of DNAme by extending our integrative methylome analyses to include both WT and *Setdb1* KO 2i ESCs.

In line with previous observations (Wu et al., 2020), SetDB1 depletion after 4-OHT treatment of *Setdb1*$^{CKO/-}$ 2i ESCs (Figure S1B) resulted in reduced cell growth, followed by massive cell death at days 4 and 5 (Figures 4A and S1D). As expected, the chromatin landscape of 2i ESCs is largely demethylated, which persisted upon SetDB1 depletion (Figures 4B and S9A–S9C). We detected a slight global increase in DNAme after *Setdb1* KO through MS (Figure S1D) and WGBS (Figures 4B and S9A–S9C), but this increase in 2i ESCs was much smaller than in serum ESCs (Figures 1B, S1D, and S2A). The ΔDNAme↓ SetDB1 peak regions as defined in serum ESCs had slightly elevated WT DNAme levels in 2i ESCs, but these were much lower compared to serum ESCs (Figures 4C and 4D). Notably, we did not detect any substantial DNAme remodeling following *Setdb1* KO in 2i ESCs at these sites (Figure 4C). This suggests that the small DNAme deposits in 2i ESCs are maintained independently of SetDB1, at least within the 4-day time frame of *Setdb1* KO.

To provide a comparison between SetDB1-repressed targets in serum and 2i ESCs, we integrated our methylome analyses with publicly available H3K9me3 and H3K27me3 ChIP-seq data, both from WT and *Setdb1* KO 2i ESCs (Wu et al., 2020). In 2i ESCs, SetDB1 targets with elevated DNAme relative to other targets (Figure 4D: ΔDNAme↓ SetDB1 peak regions) were also densely covered with H3K9me3, with H3K9me3 levels exceeding those in serum ESCs (Figure 4E). After inducing *Setdb1* KO in 2i ESCs, these

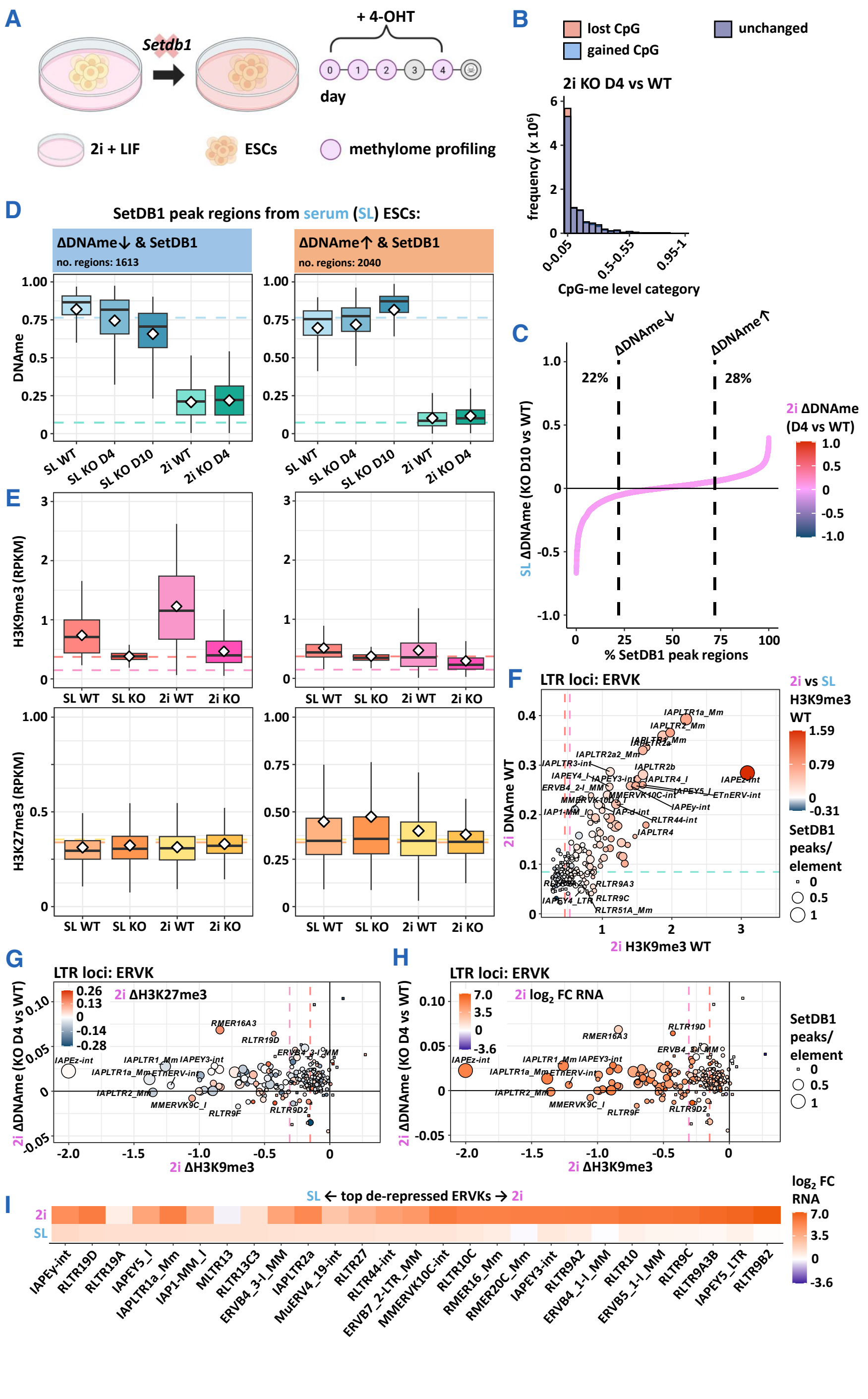

Figure 4. Hypomethylated 2i ESCs strongly depend on SetDB1-mediated H3K9me3 to repress ERVKs

(A) Schematic of workflow for methylome profiling upon induction of *Setdb1* KO in 2i ESCs.

(B) Bar graph of differential CpG methylation upon *Setdb1* KO in 2i ESCs, similar to Figure 1B.

(C) Line graph, similar to Figure 3A, but dot colors reflect DNAme change observed upon induction of *Setdb1* KO in 2i ESCs.

(D) Boxplots of DNAme in WT and *Setdb1* KO serum and 2i ESCs at target region categories defined in Figure 3A. Dotted lines reflect median WT DNAme levels in serum ESCs (light blue) and 2i ESCs (turquois). White diamonds represent averages.

(E) Boxplots of H3K9me3 and H3K27me3 in WT and KO serum and 2i ESCs at target region categories defined in (D). Dotted lines reflect median WT levels of H3K9me3 (red for serum ESCs, pink for 2i ESCs) and H3K27me3 (orange for serum ESCs, yellow for 2i ESCs). White diamonds represent averages.

(F) Scatterplot of ERVKs in 2i ESCs showing WT DNAme and WT H3K9me3 levels, and the difference in WT H3K9me3 between 2i and serum ESCs (color scale). The number of SetDB1 ChIP-seq peaks per ERVK copy in serum ESCs (within 1 kb proximity) are displayed as point size. Dotted lines represent averages of corresponding axis parameters (WT H3K9me3: red for serum ESCs, pink for 2i ESCs and WT DNAme: turquois for 2i ESCs).

(G) Scatterplot of ERVKs in 2i ESCs upon induction of *Setdb1* KO, similar to Figure S4A. Dotted lines represent averages of corresponding axis parameters (red for serum ESCs, pink for 2i ESCs).

(H) Scatterplot similar to (G), but color scale now represents the difference in expression upon induction of *Setdb1* KO, denoted as $\log_2$ fold-change.

(I) Heatmap showing expression difference of 10 most upregulated ERVKs between *Setdb1* KO and WT ESCs, cultured in serum and 2i. Expression difference is denoted as $\log_2$ fold-change.

targets underwent a profound loss of H3K9me3 (Figure 4E), despite their unaffected DNAme levels (Figure 4D). In general, H3K27me3 levels in 2i ESCs were slightly elevated at ΔDNAme↑ SetDB1 target regions as compared to ΔDNAme↓ SetDB1 targets (Figure 4E). However, this is unrelated to SetDB1 activity, as coverage remained similar following *Setdb1* KO.

Examining individual loci revealed that SetDB1-targeted ERVKs, primarily IAPs, are enriched with DNAme in 2i ESCs, albeit at much lower levels compared to serum ESCs (Figure 4F; cf; Figures S3E and S5A) (Habibi et al., 2013). Mirroring the observations from SetDB1 target regions, low DNAme levels were maintained on SetDB1-targeted IAPs and other ERVKs after *Setdb1* KO in 2i ESCs

(Figures 4G and S3E), in contrast to serum ESCs (Figures S3E and S4A), suggesting that DNMT1 maintains DNAme at these loci independently of SetDB1. These ERVKs also exhibited higher WT H3K9me3 levels in 2i ESCs compared to serum ESCs (Figure 4F; color scale), potentially offsetting the lack of extensive DNAme. The H3K9me3 enrichments that covered these ERVKs were erased after *Setdb1* KO. Integration with RNA-seq data (Wu et al., 2020) showed that SetDB1-targeted IAPs and other ERVKs exhibited much stronger transcriptional activation following *Setdb1* KO in 2i ESCs than in serum ESCs, consistent with previous studies (Figures 4H and 4I) (Deniz et al., 2018). A similar difference between 2i and serum ESCs was observed for SetDB1-bound genes regulated through the DNAme-H3K9me3 mechanism (Figure S6; cf; Figures S9D and S9E). These observations indicate a strong reliance on SetDB1-mediated H3K9me3 silencing in the hypomethylated ground-state of pluripotency. We conclude that SetDB1-dependent DNAme acts as a repressive buffer to SetDB1-mediated H3K9me3 silencing in serum ESCs, with the absence of DNAme in 2i ESCs leaving SetDB1-silenced ERVKs and genes susceptible to strong reactivation. Of note, although ERVKs are strongly reactivated after *Setdb1* KO, they are unlikely to drive the rapid cell death observed in 2i ESCs through genomic instability. Compared to delayed cell death in serum ESCs following *Setdb1* KO (Figures 1A, S1D, S1G, and S1H), loss of SetDB1 in 2i ESCs induces expression of *Ripk3*, triggering RIPK1/3-mediated necrosome assembly and activation of the necroptosis pathway (Jin et al., 2023; Wu et al., 2020).

Domain-level AlphaFold interaction modeling of SetDB1 elucidates coupling mechanisms of its regulatory axes

Our analyses above demonstrated that SetDB1-mediated DNAme and H3K9me3 are tightly coupled, whereas SetDB1 functions through PRC2, CTCF, and SMAD3 appear to be uncoupled from the DNAme-H3K9me3 axis, and/or from each other. Previous studies have suggested that protein-protein interactions between SetDB1 and DNMT enzymes (Li et al., 2006), PRC2 subunits (Fei et al., 2015), CTCF subunits (Warrier et al., 2022), and SMAD3 (Du et al., 2018; Wakabayashi et al., 2011) may underlie the regulatory functions of SetDB1. This raises the hypothesis that competitive binding for SetDB1 protein domains might dictate the coupling or uncoupling of its regulatory functions. To investigate this, we used AlphaFold3 interaction modeling to examine how interactions at the level of SetDB1 protein domains underlie its diverse functions (Abramson et al., 2024).

As a positive control, we confirmed interactions between SetDB1 and HP1α (Figure S10A)—a subunit of the SetDB1-KAP1 repressor complex—mirroring previous analyses (Tang et al., 2025). We then investigated potential SetDB1 regulation of DNMT enzymes by modeling interactions between DNMTs and flexible SetDB1 regions (Tang et al., 2025), the SetDB1 MBD (previously shown to interact with DNMT3s; Li et al., 2006), and the triple Tudor domain (TTD), a known protein-protein interaction module (Chang et al., 2021). Based on the predicted alignment error (PAE) values, we found no evidence for a direct SetDB1-DNMT1 interaction (Figure S10B), consistent with prior reports (Li et al., 2006), and no robust interaction with DNMT3a (Figure S10C). In contrast, high-confidence interactions were predicted between the SetDB1 MBD and the DNMT3b ADD domain (Figures 5A and S10D; Table S2). The DNMT3b ADD domain has a more extended interface than that of DNMT3a, previously suggested to mediate protein-protein interactions (Boyko et al., 2022), and was modeled to form an extended β-sheet with SetDB1 that is stabilized by multiple hydrogen bonds (Figures 5B, S10E, and S10F). This complex showed a high iPTM score (0.76), supporting the robustness of this model (Abramson et al., 2024). Importantly, DNMT3b binding to the SetDB1 MBD may occur independently of SetDB1 interactions with KAP1 and HP1α via its TTD and flexible regions (Tang et al., 2025; Zhang and He, 2024), and thus may not disrupt the SetDB1-KAP1 repressor complex. Together, these results suggest a potential structural mechanism for direct coupling between SetDB1-dependent DNAme and H3K9me3 deposition.

We next modeled interactions between SetDB1 and PRC2, SMAD3, and CTCF. Fei et al. (2015) reported a minor co-immunoprecipitation of EZH2 with SetDB1, and stronger associations with SUZ12. Consistent with this, we did not detect a confident SetDB1-EZH2 interaction (Figure S11A), whereas stronger interactions were predicted between SUZ12 and two SetDB1 flexible regions (396-610 and 683-1307) (Figure S11B; Table S3). The 396-610 region was also predicted to interact with SMAD3 (Figure S11C; Table S4), although modeling their structure is limited by the intrinsic disorder of flexible regions (Majila et al., 2026). Nevertheless, interactions between SetDB1 and SUZ12, and SMAD3, could interfere with HP1α binding, and with each other, potentially decoupling these interactions from the H3K9me3-DNAme axis, and from each other. In addition, strong interactions were predicted between the SetDB1 TTD and the C-terminal tail of CTCF, with a high iPTM score of 0.80 (Figures 5C, 5D, S11D, S11G, and S11H; Table S5), consistent with known roles of these domains in protein-protein interactions (Chang et al., 2021; Chernukhin et al., 2007; Ohlsson et al., 2010; Xiao et al., 2011). This CTCF-TTD interaction may contribute to uncoupling from the DNAme-H3K9me3 function of SetDB1, which relies on TTD-mediated KAP1-binding (Zhang and He, 2024). No

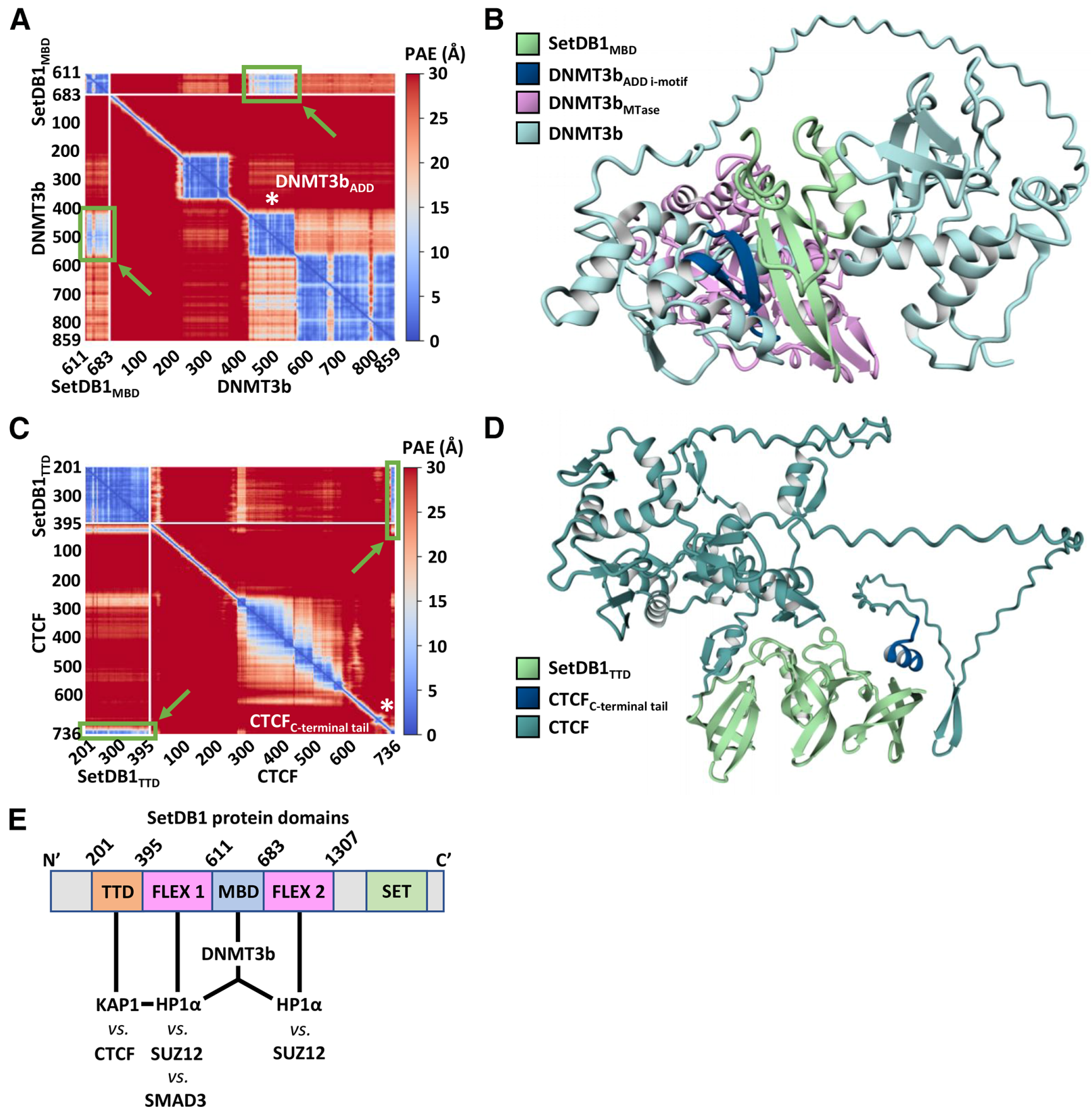

Figure 5. AlphaFold3 modeling SetDB1 protein domains reveals potential direct interactions with DNMT3b and CTCF

(A) Heatmaps of AlphaFold3-predicted interactions between MBD of SetDB1 and full-length DNMT3b from mouse. Colors represent predicted aligned error (PAE) values in angstrom, with blue indicating high-confidence and red indicating low-confidence predictions. Predicted interactions are indicated with green boxes and arrows.

(B) Modeled structure of MBD of SetDB1 (green) that interacts with DNMT3b (light blue). Interaction within the DNMT3b ADD ($DNMT3b_{ADD\ i\text{-}motif}$) and Mtase domain of DNMT3b highlighted in distinct colors (dark blue and pink, respectively).

(C) Heatmaps of AlphaFold3-predicted interactions between TTD of SetDB1 and full-length CTCF from mouse, similar to (A).

(D) Modeled structure of TTD of SetDB1 (green) that interacts with CTCF (light blue), similar to (B).

(E) Model summarizing interactions of CTCF, DNMT3b, SUZ12, and SMAD3 with the protein domains of SetDB1, as predicted by AlphaFold3. Previously reported interactions with KAP1 and HP1α are also indicated. Competitive interactions are indicated by "*vs.*".

interactions were predicted between SetDB1 and Cohesin subunits SMC1a or SMC3 (Figures S11E and S11F), suggesting that SetDB1-CTCF association is likely direct. Together, these results outline potential competitive binding at SetDB1 domains that may regulate its functional outputs (Figure 5E).

To further investigate the SetDB1 interactome, we performed co-immunoprecipitation followed by label-free quantitative LC-MS (coIP MS), employing a HeLa cell line expressing GFP-tagged SetDB1 under its endogenous promoter (Poser et al., 2008), and WT HeLa cells as control. GFP-affinity purification resulted in a strong enrichment of SetDB1 (Figure S12A), as well as its nuclear import factors ATF7IP and ATF7IP2. Consistently, our ChEP-MS showed a strong co-depletion of SetDB1 with ATF7IP/ATF7IP2 after *Setdb1* KO (Figures S12B and S12C), supporting their stable interaction. Canonical SetDB1 complex components, including KAP1/TRIM28 and HP1α/CBX3, were detected but fell just below the enrichment thresholds, although both were significantly depleted from chromatin by day 10 of *Setdb1* KO, along with associated zinc-finger proteins ($\log_2$ fold-changes ≈ −1.4 to −1.5; Figure S12D), indicating disruption of the repressor complex (Henckel et al., 2009; Kauzlaric et al., 2017; Liu et al., 2012; Quenneville et al., 2011; Takahashi et al., 2019; Wolf et al., 2020). Other interactors of SetDB1, as annotated in BioGRID, including ARL14EP, EXOC4, and EHMT2, were also enriched in our coIP MS. Notably, we identified KANSL2 as a novel SetDB1 interactor (Figure S12A). KANSL2 is a component of the NSL-type HAT complex. This observation aligns with our motif analysis (Figure 3C), which pointed to a potential role for SetDB1 in modulating histone acetylation patterns, uncoupled from its DNAme-H3K9me3 mechanism. Regarding the proteins modeled using AlphaFold3, CTCF was detected in our SetDB1 coIP MS just below the enrichment thresholds, while DNMT3b and SUZ12 showed no significant enrichment, and SMAD3 was not detected (Figure S12A). In view of the fact that interactions of SetDB1 with DNMT3b, CTCF, SUZ12, and SMAD3 were predicted by AlphaFold3 and have previously been evidenced by coIP followed by immunoblotting, but were not significantly enriched using the current coIP MS workflow, we conclude that their association with SetDB1 is likely of low affinity, requires intermediary proteins, or occurs at low stoichiometry, possibly due to a limited number of co-regulated genomic sites.

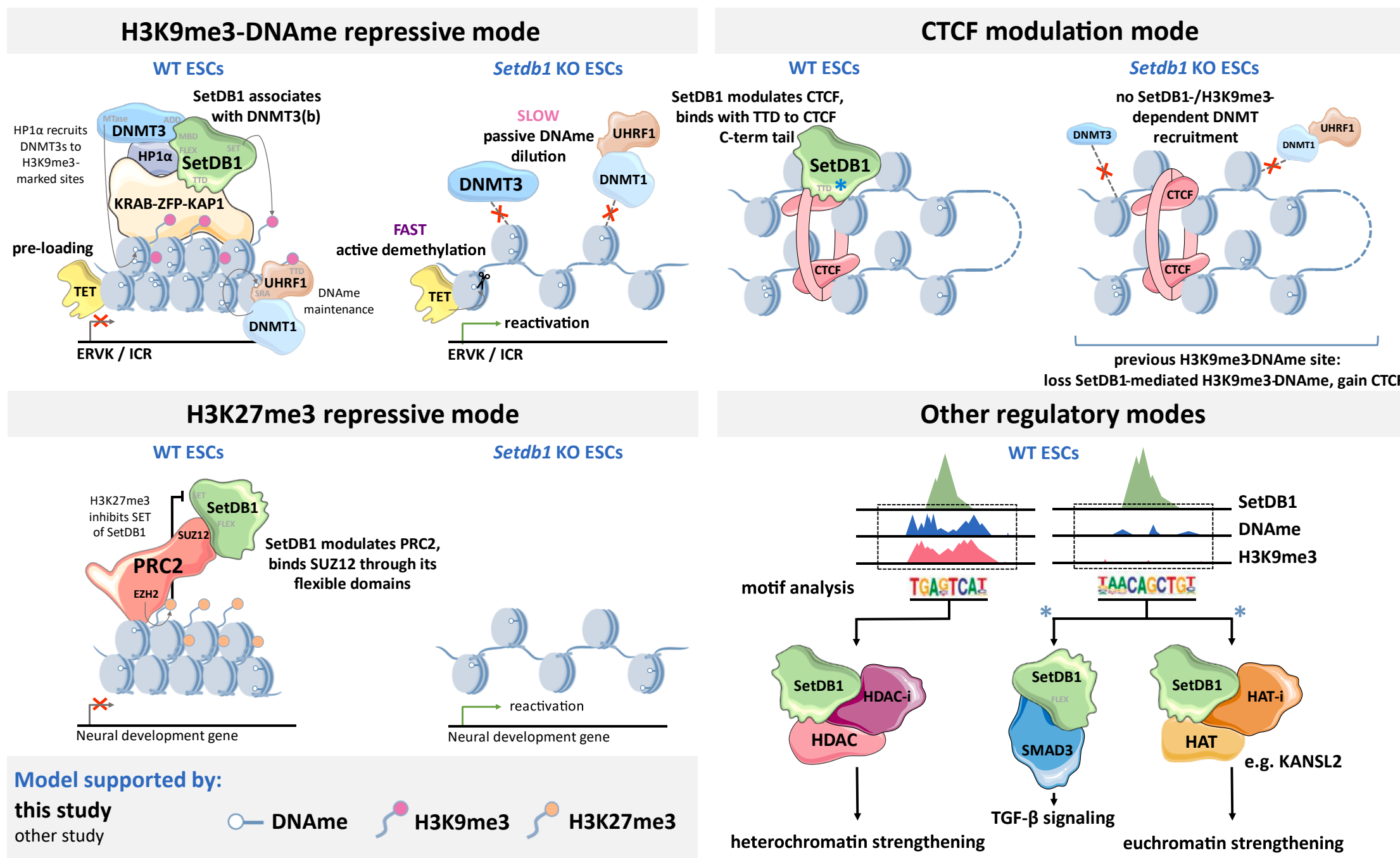

Figure 6. Hypothesized model of SetDB1-mediated regulatory mechanisms in mouse ESCs

DISCUSSION

SetDB1 has been linked to multiple epigenetic functions in mouse ESCs, including deposition of H3K9me3 and H3K27me3 (Fei et al., 2015; Karimi et al., 2011; Matsui et al., 2010), CTCF-binding (Warrier et al., 2022), and as suggested in this study, to SMAD3 regulation, and histone (de-)acetylation. However, (1) the regulation of DNAme by SetDB1 and its interplay with the other functions, and (2) the molecular interactions coordinating these functions, have largely remained enigmatic thus far. We find that ~50% of SetDB1 targets carry SetDB1-dependent DNAme, which exclusively overlaps with H3K9me3 deposition to silence ERVKs, as well as ICRs. This tight coupling suggests that SetDB1 does not regulate DNAme independently, but as part of an integrated epigenetic mechanism with H3K9me3, requiring that multiple domains of SetDB1 act in concert: SetDB1 is recruited to chromatin through its TTD-mediated interaction with KAP1 in KRAB-ZFP-KAP1 complexes (Zhang and He, 2024). These complexes contain diverse ZFPs that recognize specific DNA sequences in ERVK and ICR targets (Henckel et al., 2009; Kauzlaric et al., 2017; Liu et al., 2012; Quenneville et al., 2011; Takahashi et al., 2019; Wolf et al., 2020). The SET domain catalyzes H3K9me3 deposition (Markouli et al., 2021) at these targets, and we propose that the SetDB1 MBD may subsequently mediate interactions with DNMT enzymes.

This raises the question of which DNMT enzymes are recruited by SetDB1 or its deposited H3K9me3. DNMT1 has been shown to partially maintain DNAme at SetDB1-targeted IAPs in endoderm cells (Wang et al., 2022). However, we find no evidence for a direct SetDB1-DNMT1 interaction, consistent with previous reports (Li et al., 2006). DNMT1 may therefore reinforce SetDB1-dependent DNAme only indirectly, through UHRF1-mediated recruitment to H3K9me3-marked sites and hemimethylated DNA during replication (Fang et al., 2016; Liu et al., 2013; Ren et al., 2020; Zhao et al., 2016). With regards to the DNMT3 enzymes, while AlphaFold modeling predicted a direct interaction between SetDB1 and DNMT3b, neither our study, nor that of Warrier et al. (2022) found an enrichment of DNMT3b in SetDB1 pull-downs. Notably, DNMT3 enzymes are strongly anchored to their chromatin substrates, even under high-salt conditions (Jeong et al., 2009). Therefore, we conclude that if a direct interaction between SetDB1 and DNMT3b exists, it is likely weak. Notably, HP1α has been reported to recruit DNMT3b to H3K9me3-marked sites (Taglini et al., 2024), and our modeling suggests that their interaction could be reinforced by a potential MBD-ADD interaction between SetDB1 and DNMT3b, respectively. The H3K9me3-DNAme repressive mechanism of SetDB1 and the underlying interactions are summarized in Figures 5E and 6.

We also provide insights into the H3K9me3-DNAme mechanism of SetDB1. IAPs are enriched in SetDB1 target sites that undergo slow and limited demethylation, whereas other ERVKs are associated with rapid and extensive DNAme loss, suggesting that IAPs are protected from demethylation. These dynamics appear independent of endogenous H3K9me3 levels and its loss after KO, indicating that H3K9me3 alone does not confer protection against DNAme erasure. Instead, rapid demethylation in ESCs correlates with TET pre-loading, consistent with previous observations at the *Dazl* locus (Leung et al., 2014). Here, we show that slowly demethylating IAPs lack TET2 binding (and TET1 at internal regions), whereas rapidly demethylated ERVKs are pre-loaded with TET1 and TET2 and exhibit active DNAme turnover proportional to TET occupancy. These results support a model in which TET pre-loading determines demethylation kinetics following SetDB1 loss. It remains to be investigated what chromatin or DNA sequence-specific features shield certain ERVKs from TET pre-loading and subsequent rapid enzymatic demethylation. Nonetheless, our findings suggest that SetDB1, together with DNMT recruitment, protects against DNAme loss in WT ESCs by counteracting TET-mediated demethylation.

DNAme is tightly linked to H3K9me3, and SetDB1 associates with PRC2 to promote H3K27me3 at a subset of sites where SetDB1 does not catalyze H3K9me3 (Fei et al., 2015). This aligns with the mutual exclusivity of H3K27me3 and DNAme (Brinkman et al., 2012; van Mierlo et al., 2019), indicating that SetDB1-dependent regulation of H3K9me3-DNAme and H3K27me3 are uncoupled. AlphaFold modeling predicts interactions between SUZ12 and two SetDB1 flexible regions, which may compete with HP1α binding (Tang et al., 2025), separating these regulatory pathways. In addition, H3K27me3 has been shown to inhibit the SET domain of SetDB1 (Fei et al., 2015), further reinforcing this uncoupling. Upon *Setdb1* KO, loss of SetDB1-mediated H3K27me3 leads to reactivation of proximal neural developmental genes (Figure 6) (Fei et al., 2015). SetDB1 also regulates CTCF binding (Tam et al., 2024; Warrier et al., 2022), a factor associated with active chromatin states (Ong and Corces, 2014), indicating functions of SetDB1 beyond heterochromatin formation. We detect enrichment of CTCF motifs and peaks at SetDB1 targets that lack DNAme, H3K9me3, and H3K27me3. Based on these collective observations, we propose that SetDB1 modulates CTCF when it does not engage in epigenetic silencing. Warrier et al. (2022) showed that deletion of the SET domain does not disrupt SetDB1-CTCF co-localization, implying their connection involves different domains. Through AlphaFold modeling, we predict a direct interaction between the SetDB1 TTD and the C-terminal tail of CTCF. We find no interaction between SetDB1 and Cohesin subunits SMC1a or SMC3 in our AlphaFold modeling and coIP MS, suggesting that the SetDB1-CTCF association is not Cohesin-mediated. Instead, CTCF binding at the SetDB1 TTD may displace KAP1, providing a mechanistic explanation for the uncoupling from the DNAme-H3K9me3 mechanism of SetDB1 (Figure 6).

In summary, our study offers a comprehensive characterization of SetDB1-dependent DNAme regulation in pluripotency. By integrating ChIP- and RNA-seq, ChEP-MS, AlphaFold modeling, and coIP MS, we define a complex regulatory network linking SetDB1-dependent DNAme to multiple epigenetic pathways. We cannot exclude additional regulatory modes, including SMAD3 involvement, as TGF-β production and signaling were disturbed at the chromatin level in SetDB1-deficient ESCs. Moreover, the SetDB1 396-610 region is predicted to interact with SMAD3, potentially uncoupling this axis from the H3K9me3-DNAme mechanism, similar to SUZ12. Consistently, SMAD3 motifs are enriched at SetDB1 targets lacking H3K9me3-DNAme. Beyond this, SetDB1 may also regulate histone acetylation. Motifs for HDAC- and HAT-associated TFs are differentially enriched at SetDB1 target sites with or without DNAme-H3K9me3, and coIP MS identifies enrichment of KANSL2, an NSL-type HAT complex component. Together with prior evidence for HDAC associations (Yang et al., 2003), and even potential non-nuclear functions of SetDB1 (Markouli et al., 2021; Rapone et al., 2023), these data suggest broader SetDB1 regulatory functions beyond heterochromatin formation. Future research efforts might further investigate the regulatory functions of SetDB1 and the underlying mechanisms at the level of SetDB1's protein domains, including mutant studies that disrupt interactions mediated by the domains identified in this study.

RESOURCE AVAILABILITY

Lead contact

Requests for further information should be directed to and will be fulfilled by the lead contact, Hendrik Marks (hendrik.marks@ru.nl).

Materials availability

This study did not generate new unique reagents.

Data and code availability

- The WGBS datasets produced in this study are available at NCBI Gene Expression Omnibus (GEO) through accession number GEO: GSE299458. The MS datasets from the ChEP-MS and coIP MS experiments generated in this study are available at the EMBL Proteomics IDEntification database (PRIDE) through the dataset identifiers PRIDE: PXD074325 and PRIDE: PXD074363, respectively.
- This study did not generate new code.

ACKNOWLEDGMENTS

We thank Yoichi Shinkai for providing the *Setdb1*$^{CKO/-}$ ESC (as used throughout this study), Cynthia Fisher for providing the alternative *Setdb1*$^{CKO/-}$ ESC, Ina Poser and Tony Hyman for providing the WT and SetDB1-GFP HeLa BAC clone cell line (used for the coIP MS assay), Pascal Jansen for help with the coIP, and Dick Zijlmans and Marius Joskin for mass spectrometric analyses. The Marks lab is supported by a Nederlandse Organisatie voor Wetenschappelijk Onderzoek (NWO) XL grant (OCENW.XL21.XL21.100), a ZonMW Open grant (09120232310096), and a Radboud Science faculty grant (IRP voucher). The Lorincz lab is supported by a CIHR grant (PJT-190055).

AUTHOR CONTRIBUTIONS

Conceptualization, N.G.P.B., M.L., H.G.S., and H.M.; data curation, N.G.P.B., E.H., Z.L., J.A., A.K.K., and J.W.; formal analysis, N.G.P.B., S.H.A.H., S.F., D.D., K.C.M.B., I.M., J.A., J.W., and H.M.; funding acquisition, H.G.S. and H.M.; methodology, N.G.P.B., S.H.A.H., S.F., D.D., K.C.M.B., J.A., I.M., J.W., J.H.J., and A.B.B.; project administration, H.M.; supervision, H.G.S. and H.M.; visualization, N.G.P.B.; writing – original draft, N.G.P.B. and H.M.; writing – review and editing, N.G.P.B., E.H., S.H.A.H., Z.L., S.F., D.D., K.C.M.B., J.A., I.M., A.K.K., J.W., J.H.J., A.B.B., K.M., M.V., M.L., H.G.S., and H.M.

DECLARATION OF INTERESTS

The authors declare no competing interests.

STAR★METHODS

Detailed methods are provided in the online version of this paper and include the following:

- KEY RESOURCES TABLE
- EXPERIMENTAL MODEL AND STUDY PARTICIPANT DETAILS
  - Cell culture of mouse ESCs
  - Cell culture of HeLa cells
- METHOD DETAILS
  - Quantitative PCR
  - DNAme measurements by MS
  - Hairpin bisulfite sequencing and data processing
  - Whole-genome bisulfite sequencing and data processing
  - Binning of whole-genome bisulfite sequencing data
  - ChIP-seq data processing
  - Transcription factor motif analysis
  - RNA-seq data processing
  - Nuclear isolation from HeLa cells and SetDB1 co-immunoprecipitation followed by MS
  - ChEP-MS
  - Processing of coIP and ChEP MS data
  - AlphaFold 3 multimer model predictions
- QUANTIFICATION AND STATISTICAL ANALYSIS
  - Replication and statistical procedures

SUPPLEMENTAL INFORMATION

Supplemental information can be found online at https://doi.org/10.1016/j.stemcr.2026.103075.

Received: August 6, 2025
Revised: August 2, 2026
Accepted: August 7, 2026
Published: September 3, 2026

REFERENCES

Abramson, J., Adler, J., Dunger, J., Evans, R., Green, T., Pritzel, A., Ronneberger, O., Willmore, L., Ballard, A.J., Bambrick, J., et al. (2024). Accurate structure prediction of biomolecular interactions with AlphaFold 3. Nature *630*, 493–500. https://doi.org/10.1038/s41586-024-07487-w.

Altschul, S.F., Gish, W., Miller, W., Myers, E.W., and Lipman, D.J. (1990). Basic local alignment search tool. J. Mol. Biol. *215*, 403–410. https://doi.org/10.1016/S0022-2836(05)80360-2.

Arand, J., Spieler, D., Karius, T., Branco, M.R., Meilinger, D., Meissner, A., Jenuwein, T., Xu, G., Leonhardt, H., Wolf, V., et al. (2012). In vivo control of CpG and non-CpG DNA methylation by DNA methyltransferases. PLoS Genet. *8*, e1002750. https://doi.org/10.1371/journal.pgen.1002750.

Barral, A., Pozo, G., Ducrot, L., Papadopoulos, G.L., Sauzet, S., Oldfield, A.J., Cavalli, G., Déjardin, J., and Déjardin, J. (2022). SETDB1/NSD-dependent H3K9me3/H3K36me3 dual heterochromatin maintains gene expression profiles by bookmarking poised enhancers. Mol. Cell *82*, 816–832.e12. https://doi.org/10.1016/j.molcel.2021.12.037.

Bilodeau, S., Kagey, M.H., Frampton, G.M., Rahl, P.B., and Young, R.A. (2009). SetDB1 contributes to repression of genes encoding developmental regulators and maintenance of ES cell state. Genes Dev *23*. 2484–9. https://doi.org/10.1101/gad.1837309.

Boyko, K., Arkova, O., Nikolaeva, A., Popov, V.O., Georgiev, P., and Bonchuk, A. (2022). Structure of the DNMT3B ADD domain suggests the absence of a DNMT3A-like autoinhibitory mechanism. Biochem. Biophys. Res. Commun. *619*, 124–129. https://doi.org/10.1016/j.bbrc.2022.06.036.

Brinkman, A.B., Gu, H., Bartels, S.J.J., Zhang, Y., Matarese, F., Simmer, F., Marks, H., Bock, C., Gnirke, A., Meissner, A., et al. (2012). Sequential ChIP-bisulfite sequencing enables direct genome-scale investigation of chromatin and DNA methylation cross-talk. Genome Res. *22*, 1128–1138. https://doi.org/10.1101/gr.133728.111.

Bruse, N., and Heeringen, S.J.V. (2018). GimmeMotifs: an analysis framework for transcription factor motif analysis. Preprint at bioRxiv, 474403. https://doi.org/10.1101/474403.

Chadwick, B.P., and Willard, H.F. (2004). Multiple spatially distinct types of facultative heterochromatin on the human inactive X chromosome. Proc. Natl. Acad. Sci. USA *101*, 17450–17455. https://doi.org/10.1073/pnas.0408021101.

Chang, L., Campbell, J., Raji, I.O., Guduru, S.K.R., Kandel, P., Nguyen, M., Liu, S., Tran, K., Venugopal, N.K., Taylor, B.C., et al. (2021). Discovery of small molecules targeting the tandem Tudor domain of the epigenetic factor UHRF1 using fragment-based ligand discovery. Sci. Rep. *11*, 1121. https://doi.org/10.1038/s41598-020-80588-4.

Chernukhin, I., Shamsuddin, S., Kang, S.Y., Bergström, R., Kwon, Y.W., Yu, W., Whitehead, J., Mukhopadhyay, R., Docquier, F.,

Farrar, D., et al. (2007). CTCF Interacts with and Recruits the Largest Subunit of RNA Polymerase II to CTCF Target Sites Genome-Wide. Mol. Cell. Biol. *27*, 1631–1648. https://doi.org/10.1128/MCB.01993-06.

de la Rica, L., Deniz, Ö., Cheng, K.C.L., Todd, C.D., Cruz, C., Houseley, J., and Branco, M.R. (2016). TET-dependent regulation of retrotransposable elements in mouse embryonic stem cells. Genome Biol. *17*, 234. https://doi.org/10.1186/s13059-016-1096-8.

Demichev, V., Messner, C.B., Vernardis, S.I., Lilley, K.S., and Ralser, M. (2020). DIA-NN: neural networks and interference correction enable deep proteome coverage in high throughput. Nat. Methods *17*, 41–44. https://doi.org/10.1038/s41592-019-0638-x.

Deniz, Ö., de la Rica, L., Cheng, K.C.L., Spensberger, D., and Branco, M.R. (2018). SETDB1 prevents TET2-dependent activation of IAP retroelements in naïve embryonic stem cells. Genome Biol. *19*, 6–11. https://doi.org/10.1186/s13059-017-1376-y.

Dodge, J.E., Kang, Y.K., Beppu, H., Lei, H., and Li, E. (2004). Histone H3-K9 methyltransferase ESET is essential for early development. Mol. Cell. Biol. *24*, 2478–2486. https://doi.org/10.1128/MCB.24.6.2478-2486.2004.

Du, D., Katsuno, Y., Meyer, D., Budi, E.H., Chen, S.H., Koeppen, H., Wang, H., Akhurst, R.J., Derynck, R., and Derynck, R. (2018). Smad3-mediated recruitment of the methyltransferase SETDB1/ESET controls Snail1 expression and epithelial–mesenchymal transition. EMBO Rep. *19*, 135–155. https://doi.org/10.15252/embr.201744250.

Fang, J., Cheng, J., Wang, J., Zhang, Q., Liu, M., Gong, R., Wang, P., Zhang, X., Feng, Y., Lan, W., et al. (2016). Hemi-methylated DNA opens a closed conformation of UHRF1 to facilitate its histone recognition. Nat. Commun. *7*, 11197. https://doi.org/10.1038/ncomms11197.

Fei, Q., Yang, X., Jiang, H., Wang, Q., Yu, Y., Yu, Y., Yi, W., Zhou, S., Chen, T., Lu, C., et al. (2015). SETDB1 modulates PRC2 activity at developmental genes independently of H3K9 trimethylation in mouse ES cells. Genome Res. *25*, 1325–1335. https://doi.org/10.1101/gr.177576.114.

Ficz, G., Hore, T.A., Santos, F., Lee, H.J., Dean, W., Arand, J., Krueger, F., Oxley, D., Paul, Y.L., Walter, J., et al. (2013). FGF signaling inhibition in ESCs drives rapid genome-wide demethylation to the epigenetic ground state of pluripotency. Cell Stem Cell *13*, 351–359. https://doi.org/10.1016/j.stem.2013.06.004.

Fisher, C.L., Marks, H., Cho, L.T.Y., Andrews, R., Wormald, S., Carroll, T., Iyer, V., Tate, P., Rosen, B., Stunnenberg, H.G., et al. (2017). An efficient method for generation of bi-allelic null mutant mouse embryonic stem cells and its application for investigating epigenetic modifiers. Nucleic Acids Res. *45*, e174. https://doi.org/10.1093/nar/gkx811.

Habibi, E., Brinkman, A.B., Arand, J., Kroeze, L.I., Kerstens, H.H.D., Matarese, F., Lepikhov, K., Gut, M., Brun-Heath, I., Hubner, N.C., et al. (2013). Whole-genome bisulfite sequencing of two distinct interconvertible DNA methylomes of mouse embryonic stem cells. Cell Stem Cell *13*, 360–369. https://doi.org/10.1016/j.stem.2013.06.002.

Hekkelman, M.L., and Vriend, G. (2005). MRS: a fast and compact retrieval system for biological data. Nucleic Acids Res. *33*, W766–W769. https://doi.org/10.1093/nar/gki422.

Henckel, A., Nakabayashi, K., Sanz, L.A., Feil, R., Hata, K., and Arnaud, P. (2009). Histone methylation is mechanistically linked to DNA methylation at imprinting control regions in mammals. Hum. Mol. Genet. *18*, 3375–3383. https://doi.org/10.1093/hmg/ddp277.

Huber, W., Von Heydebreck, A., Sültmann, H., Poustka, A., and Vingron, M. (2002). Variance stabilization applied to microarray data calibration and to the quantification of differential expression. Bioinformatics *18*, S96–S104. https://doi.org/10.1093/bioinformatics/18.suppl_1.s96.

Jeong, S., Liang, G., Sharma, S., Lin, J.C., Choi, S.H., Han, H., Yoo, C.B., Egger, G., Yang, A.S., Jones, P.A., and Jones, P.A. (2009). Selective anchoring of DNA methyltransferases 3A and 3B to nucleosomes containing methylated DNA. Mol. Cell. Biol. *29*, 5366–5376. https://doi.org/10.1128/MCB.00484-09.

Jin, L., He, J., Feng, H., Li, S., Liu, H., Dong, H., Hu, M., Huang, J., Wu, H., Chen, J., et al. (2023). Transposable elements activation triggers necroptosis in mouse embryonic stem cells. Cell Death Dis. *14*, 184. https://doi.org/10.1038/s41419-023-05705-3.

Karimi, M.M., Goyal, P., Maksakova, I.A., Bilenky, M., Leung, D., Tang, J.X., Shinkai, Y., Mager, D.L., Jones, S., Hirst, M., et al. (2011). DNA methylation and SETDB1/H3K9me3 regulate predominantly distinct sets of genes, retroelements, and chimeric transcripts in mESCs. Cell Stem Cell *8*, 676–687. https://doi.org/10.1016/j.stem.2011.04.004.

Kauzlaric, A., Ecco, G., Cassano, M., Duc, J., Imbeault, M., and Trono, D. (2017). The mouse genome displays highly dynamic populations of KRAB-zinc finger protein genes and related genetic units. PLoS One *12*, e0173746. https://doi.org/10.1371/journal.pone.0173746.

Krieger, E., and Vriend, G. (2014). YASARA View—molecular graphics for all devices—from smartphones to workstations. Bioinformatics *30*, 2981–2982. https://doi.org/10.1093/bioinformatics/btu426.

Krieger, E., and Vriend, G. (2015). New ways to boost molecular dynamics simulations. J. Comput. Chem. *36*, 996–1007. https://doi.org/10.1002/jcc.23899.

Krueger, F., and Andrews, S.R. (2011). Bismark: a flexible aligner and methylation caller for Bisulfite-Seq applications. Bioinformatics *27*, 1571–1572. https://doi.org/10.1093/bioinformatics/btr167.

Kulis, M., Heath, S., Bibikova, M., Queirós, A.C., Navarro, A., Clot, G., Martínez-Trillos, A., Castellano, G., Brun-Heath, I., Pinyol, M., et al. (2012). Epigenomic analysis detects widespread gene-body DNA hypomethylation in chronic lymphocytic leukemia. Nat. Genet. *44*, 1236–1242. https://doi.org/10.1038/ng.2443.

Kustatscher, G., Wills, K.L.H., Furlan, C., and Rappsilber, J. (2014). Chromatin enrichment for proteomics. Nat. Protoc. *9*, 2090–2099. https://doi.org/10.1038/nprot.2014.142.

Land, H., and Humble, M.S. (2017). YASARA: a tool to obtain structural guidance in biocatalytic investigations. In Protein

engineering: methods and protocols, U.T. Bornscheuer and M. Höhne, eds. (Springer New York), pp. 43–67.

Langmead, B., and Salzberg, S.L. (2012). Fast gapped-read alignment with Bowtie 2. Nat. Methods *9*, 357–359. https://doi.org/10.1038/nmeth.1923.

Leitch, H.G., McEwen, K.R., Turp, A., Encheva, V., Carroll, T., Grabole, N., Mansfield, W., Nashun, B., Knezovich, J.G., Smith, A., et al. (2013). Naive pluripotency is associated with global DNA hypomethylation. Nat. Struct. Mol. Biol. *20*, 311–316. https://doi.org/10.1038/nsmb.2510.

Lerdrup, M., Johansen, J.V., Agrawal-Singh, S., and Hansen, K. (2016). An interactive environment for agile analysis and visualization of ChIP-sequencing data. Nat. Struct. Mol. Biol. *23*, 349–357. https://doi.org/10.1038/nsmb.3180.

Leung, D.C., and Lorincz, M.C. (2012). Silencing of endogenous retroviruses: when and why do histone marks predominate? Trends Biochem. Sci. *37*, 127–133. https://doi.org/10.1016/j.tibs.2011.11.006.

Leung, D., Du, T., Wagner, U., Xie, W., Lee, A.Y., Goyal, P., Li, Y., Szulwach, K.E., Jin, P., Lorincz, M.C., et al. (2014). Regulation of DNA methylation turnover at LTR retrotransposons and imprinted loci by the histone methyltransferase Setdb1. Proc. Natl. Acad. Sci. USA *111*, 6690–6695. https://doi.org/10.1073/pnas.1322273111.

Li, H., Rauch, T., Chen, Z.X., Szabó, P.E., Riggs, A.D., and Pfeifer, G.P. (2006). The histone methyltransferase SETDB1 and the DNA methyltransferase DNMT3A interact directly and localize to promoters silenced in cancer cells. J. Biol. Chem. *281*, 19489–19500. https://doi.org/10.1074/jbc.M513249200.

Lio, C.W.J., Yue, X., López-Moyado, I.F., Tahiliani, M., Aravind, L., and Rao, A. (2020). TET methylcytosine oxidases: new insights from a decade of research. J. Biosci. *45*, 21.

Liu, S., Brind'Amour, J., Karimi, M.M., Shirane, K., Bogutz, A., Lefebvre, L., Sasaki, H., Shinkai, Y., Lorincz, M.C., and Lorincz, M.C. (2014). Setdb1 is required for germline development and silencing of H3K9me3-marked endogenous retroviruses in primordial germ cells. Genes Dev. *28*, 2041–2055. https://doi.org/10.1101/gad.244848.114.

Liu, T., Xu, P., Ke, S., Dong, H., Zhan, M., Hu, Q., and Li, J. (2022). Histone methyltransferase SETDB1 inhibits TGF-β-induced epithelial–mesenchymal transition in pulmonary fibrosis by regulating SNAI1 expression and the ferroptosis signaling pathway. Arch. Biochem. Biophys. *715*, 109087. https://doi.org/10.1016/j.abb.2021.109087.

Liu, X., Gao, Q., Li, P., Zhao, Q., Zhang, J., Li, J., Koseki, H., Wong, J., and Wong, J. (2013). UHRF1 targets DNMT1 for DNA methylation through cooperative binding of hemi-methylated DNA and methylated H3K9. Nat. Commun. *4*, 1563. https://doi.org/10.1038/ncomms2562.

Liu, Y., Toh, H., Sasaki, H., Zhang, X., and Cheng, X. (2012). An atomic model of Zfp57 recognition of CpG methylation within a specific DNA sequence. Genes Dev. *26*, 2374–2379. https://doi.org/10.1101/gad.202200.112.

Love, M.I., Huber, W., and Anders, S. (2014). Moderated estimation of fold change and dispersion for RNA-seq data with DESeq2. Genome Biol. *15*, 550. https://doi.org/10.1186/s13059-014-0550-8.

Madeira, F., Madhusoodanan, N., Lee, J., Eusebi, A., Niewielska, A., Tivey, A.R.N., Lopez, R., Butcher, S., and Butcher, S. (2024). The EMBL-EBI Job Dispatcher sequence analysis tools framework in 2024. Nucleic Acids Res. *52*, W521–W525. https://doi.org/10.1093/nar/gkae241.

Majila, K., Ullanat, V., and Viswanath, S. (2026). Disobind: A sequence-based, partner-dependent contact map and interface residue predictor for intrinsically disordered regions. Cell Syst *17*, 101486. https://doi.org/10.1016/j.cels.2025.101486.

Maksakova, I.A., Romanish, M.T., Gagnier, L., Dunn, C.A., Van de Lagemaat, L.N., and Mager, D.L. (2006). Retroviral elements and their hosts: insertional mutagenesis in the mouse germ line. PLoS Genet. *2*, e2. https://doi.org/10.1371/journal.pgen.0020002.

Markouli, M., Strepkos, D., and Piperi, C. (2021). Structure, activity and function of the SETDB1 protein methyltransferase. Life *11*, 817. https://doi.org/10.3390/life11080817.

Matsui, T., Leung, D., Miyashita, H., Maksakova, I.A., Miyachi, H., Kimura, H., Tachibana, M., Lorincz, M.C., Shinkai, Y., and Shinkai, Y. (2010). Proviral silencing in embryonic stem cells requires the histone methyltransferase ESET. Nature *464*, 927–931. https://doi.org/10.1038/nature08858.

Monteagudo-Sánchez, A., Noordermeer, D., and Greenberg, M.V.C. (2024). The impact of DNA methylation on CTCF-mediated 3D genome organization. Nat. Struct. Mol. Biol. *31*, 404–412. https://doi.org/10.1038/s41594-024-01241-6.

Ohlsson, R., Lobanenkov, V., and Klenova, E. (2010). Does CTCF mediate between nuclear organization and gene expression? Bioessays *32*, 37–50. https://doi.org/10.1002/bies.200900118.

Ong, C.T., and Corces, V.G. (2014). CTCF: an architectural protein bridging genome topology and function. Nat. Rev. Genet. *15*, 234–246. https://doi.org/10.1038/nrg3663.

Pauler, F.M., Sloane, M.A., Huang, R., Regha, K., Koerner, M.V., Tamir, I., Sommer, A., Aszodi, A., Jenuwein, T., Barlow, D.P., and Barlow, D.P. (2009). H3K27me3 forms BLOCs over silent genes and intergenic regions and specifies a histone banding pattern on a mouse autosomal chromosome. Genome Res. *19*, 221–233. https://doi.org/10.1101/gr.080861.108.

Poser, I., Sarov, M., Hutchins, J.R.A., Hériché, J.K., Toyoda, Y., Pozniakovsky, A., Weigl, D., Nitzsche, A., Hegemann, B., Bird, A.W., et al. (2008). BAC TransgeneOmics: a high-throughput method for exploration of protein function in mammals. Nat. Methods *5*, 409–415. https://doi.org/10.1038/nmeth.1199.

Quenneville, S., Verde, G., Corsinotti, A., Kapopoulou, A., Jakobsson, J., Offner, S., Baglivo, I., Pedone, P.V., Grimaldi, G., Riccio, A., et al. (2011). In embryonic stem cells, ZFP57/KAP1 recognize a methylated hexanucleotide to affect chromatin and DNA methylation of imprinting control regions. Mol. Cell *44*, 361–372. https://doi.org/10.1016/j.molcel.2011.08.032.

Rapone, R., Del Maestro, L., Bouyioukos, C., Albini, S., Cruz-Tapias, P., Joliot, V., Cosson, B., Ait-Si-Ali, S., and Ait-Si-Ali, S. (2023). The cytoplasmic fraction of the histone lysine methyltransferase Setdb1 is essential for embryonic stem cells. iScience *26*, 107386. https://doi.org/10.1016/j.isci.2023.107386.

Ren, W., Fan, H., Grimm, S.A., Guo, Y., Kim, J.J., Yin, J., Li, L., Petell, C.J., Tan, X.F., Zhang, Z.M., et al. (2020). Direct readout of heterochromatic H3K9me3 regulates DNMT1-mediated maintenance DNA methylation. Proc. Natl. Acad. Sci. USA *117*, 18439–18447. https://doi.org/10.1073/pnas.2009316117.

Shen, L., Wu, H., Diep, D., Yamaguchi, S., D'Alessio, A.C., Fung, H.L., Zhang, K., Zhang, Y., and Zhang, Y. (2013). Genome-wide analysis reveals TET-and TDG-dependent 5-methylcytosine oxidation dynamics. Cell *153*, 692–706. https://doi.org/10.1016/j.cell.2013.04.002.

Sievers, F., Wilm, A., Dineen, D., Gibson, T.J., Karplus, K., Li, W., Lopez, R., McWilliam, H., Remmert, M., Söding, J., et al. (2011). Fast, scalable generation of high-quality protein multiple sequence alignments using Clustal Omega. Mol. Syst. Biol. *7*, 539. https://doi.org/10.1038/msb.2011.75.

Squazzo, S.L., O'Geen, H., Komashko, V.M., Krig, S.R., Jin, V.X., Jang, S.W., Margueron, R., Reinberg, D., Green, R., Farnham, P.J., and Farnham, P.J. (2006). Suz12 binds to silenced regions of the genome in a cell-type-specific manner. Genome Res. *16*, 890–900. https://doi.org/10.1101/gr.5306606.

Taglini, F., Kafetzopoulos, I., Rolls, W., Musialik, K.I., Lee, H.Y., Zhang, Y., Marenda, M., Kerr, L., Finan, H., Rubio-Ramon, C., et al. (2024). DNMT3B PWWP mutations cause hypermethylation of heterochromatin. EMBO Rep. *25*, 1130–1155. https://doi.org/10.1038/s44319-024-00061-5.

Takahashi, N., Coluccio, A., Thorball, C.W., Planet, E., Shi, H., Offner, S., Turelli, P., Imbeault, M., Ferguson-Smith, A.C., Trono, D., and Trono, D. (2019). ZNF445 is a primary regulator of genomic imprinting. Genes Dev. *33*, 49–54. https://doi.org/10.1101/gad.320069.118.

Tam, P.L.F., Cheung, M.F., Chan, L.Y., and Leung, D. (2024). Cell-type differential targeting of SETDB1 prevents aberrant CTCF binding, chromatin looping, and cis-regulatory interactions. Nat. Commun. *15*, 15. https://doi.org/10.1038/s41467-023-44578-0.

Tang, Q., Zhang, A., Sullivan, M., Fejes Toth, K., and Aravin, A.A. (2025). Auto-methylation of the histone methyltransferase SetDB1 at its histone-mimic motifs ensures the spreading and maintenance of heterochromatin. Preprint at bioRxiv, 2025. https://doi.org/10.1101/2025.01.21.634156.

Teissandier, A., Servant, N., Barillot, E., and Bourc'his, D. (2019). Tools and best practices for retrotransposon analysis using high-throughput sequencing data. Mob. DNA *10*, 52. https://doi.org/10.1186/s13100-019-0192-1.

van der Sande, M., Frölich, S., Schäfers, T., Smits, J.G.A., Snabel, R.R., Rinzema, S., and van Heeringen, S.J. (2023). Seq2science: an end-to-end workflow for functional genomics analysis. PeerJ *11*, e16380. https://doi.org/10.7717/peerj.16380.

van Mierlo, G., Dirks, R.A.M., De Clerck, L., Brinkman, A.B., Huth, M., Kloet, S.L., Saksouk, N., Kroeze, L.I., Willems, S., Farlik, M., et al. (2019). Integrative proteomic profiling reveals PRC2-dependent epigenetic crosstalk maintains ground-state pluripotency. Cell Stem Cell *24*, 123–137.e8. https://doi.org/10.1016/j.stem.2018.10.017.

Wakabayashi, Y., Tamiya, T., Takada, I., Fukaya, T., Sugiyama, Y., Inoue, N., Kimura, A., Morita, R., Kashiwagi, I., Takimoto, T., et al. (2011). Histone 3 lysine 9 (H3K9) methyltransferase recruitment to the interleukin-2 (IL-2) promoter is a mechanism of suppression of IL-2 transcription by the transforming growth factor-β-Smad pathway. J. Biol. Chem. *286*, 35456–35465. https://doi.org/10.1074/jbc.M111.236794.

Wang, Z., Fan, R., Russo, A., Cernilogar, F.M., Nuber, A., Schirge, S., Shcherbakova, I., Dzhilyanova, I., Ugur, E., Anton, T., et al. (2022). Dominant role of DNA methylation over H3K9me3 for IAP silencing in endoderm. Nat. Commun. *13*, 5447. https://doi.org/10.1038/s41467-022-32978-7.

Warrier, T., El Farran, C., Zeng, Y., Ho, B.S.Q., Bao, Q., Zheng, Z.H., Bi, X., Ng, H.H., Ong, D.S.T., Chu, J.J.H., et al. (2022). SETDB1 acts as a topological accessory to Cohesin via an H3K9me3-independent, genomic shunt for regulating cell fates. Nucleic Acids Res. *50*, 7326–7349. https://doi.org/10.1093/nar/gkac531.

Waskom, M. (2021). Seaborn: statistical data visualization. J. Open Source Softw. *6*, 3021. https://doi.org/10.21105/joss.03021.

Waterhouse, A.M., Procter, J.B., Martin, D.M.A., Clamp, M., and Barton, G.J. (2009). Jalview Version 2—a multiple sequence alignment editor and analysis workbench. Bioinformatics *25*, 1189–1191. https://doi.org/10.1093/bioinformatics/btp033.

Williams, K., Christensen, J., Pedersen, M.T., Johansen, J.V., Cloos, P.A.C., Rappsilber, J., and Helin, K. (2011). TET1 and hydroxymethylcytosine in transcription and DNA methylation fidelity. Nature *473*, 343–348. https://doi.org/10.1038/nature10066.

Wolf, G., de Iaco, A., Sun, M.A., Bruno, M., Tinkham, M., Hoang, D., Mitra, A., Ralls, S., Trono, D., Macfarlan, T.S., and Macfarlan, T.S. (2020). KRAB-zinc finger protein gene expansion in response to active retrotransposons in the murine lineage. eLife *9*, e56337. https://doi.org/10.7554/eLife.56337.

Wu, K., Liu, H.E., Wang, Y., He, J., Xu, S., Chen, Y., Kuang, J., Liu, J., Guo, L., Li, D., et al. (2020). SETDB1-mediated cell fate transition between 2C-like and pluripotent states. Cell Rep. *30*, 25–36.e6. https://doi.org/10.1016/j.celrep.2019.12.010.

Xiao, T., Wallace, J., and Felsenfeld, G. (2011). Specific sites in the C terminus of CTCF interact with the SA2 subunit of the cohesin complex and are required for cohesin-dependent insulation activity. Mol. Cell. Biol. *31*, 2174–2183. https://doi.org/10.1128/MCB.05093-11.

Xiong, J., Zhang, Z., Chen, J., Huang, H., Xu, Y., Ding, X., Zheng, Y., Nishinakamura, R., Xu, G.L., Wang, H., et al. (2016). Cooperative action between SALL4A and TET proteins in stepwise oxidation of 5-methylcytosine. Mol. Cell *64*, 913–925. https://doi.org/10.1016/j.molcel.2016.10.013.

Yang, L., Mei, Q., Zielinska-Kwiatkowska, A., Matsui, Y., Blackburn, M.L., Benedetti, D., Krumm, A.A., Taborsky, G.J., Jr., Chansky, H.A., and Chansky, H.A. (2003). An ERG (ets-related gene)-associated histone methyltransferase interacts with histone deacetylases 1/2 and transcription co-repressors mSin3A/B. Biochem. J. *369*, 651–657. https://doi.org/10.1042/BJ20020854.

Ying, Q.L., Wray, J., Nichols, J., Batlle-Morera, L., Doble, B., Woodgett, J., Cohen, P., Smith, A., and Smith, A. (2008). The

ground state of embryonic stem cell self-renewal. nature *453*, 519–523. https://doi.org/10.1038/nature06968.

Yuan, P., Han, J., Guo, G., Orlov, Y.L., Huss, M., Loh, Y.H., Yaw, L.P., Robson, P., Lim, B., Ng, H.H., and Ng, H.H. (2009). Eset partners with Oct4 to restrict extraembryonic trophoblast lineage potential in embryonic stem cells. Genes Dev. *23*, 2507–2520. https://doi.org/10.1101/gad.1831909.

Yu, G., Wang, L.G., and He, Q.Y. (2015). ChIPseeker: an R/Bioconductor package for ChIP peak annotation, comparison and visualization. Bioinformatics *31*, 2382–2383. https://doi.org/10.1093/bioinformatics/btv145.

Zhang, Y., Liu, T., Meyer, C.A., Eeckhoute, J., Johnson, D.S., Bernstein, B.E., Nusbaum, C., Myers, R.M., Brown, M., Li, W., et al. (2008). Model-based analysis of ChIP-Seq (MACS). Genome Biol. *9*, R137. https://doi.org/10.1186/gb-2008-9-9-r137.

Zhang, E., and He, P. (2024). The function of histone methyltransferase SETDB1 and its roles in liver cancer. Front. Cell Dev. Biol. *12*, 1500263. https://doi.org/10.3389/fcell.2024.1500263.

Zhao, Q., Zhang, J., Chen, R., Wang, L., Li, B., Cheng, H., Duan, X., Zhu, H., Wei, W., Li, J., et al. (2016). Dissecting the precise role of H3K9 methylation in crosstalk with DNA maintenance methylation in mammals. Nat. Commun. *7*, 12464. https://doi.org/10.1038/ncomms12464.

Zijlmans, D.W., Talon, I., Verhelst, S., Bendall, A., Van Nerum, K., Javali, A., Malcolm, A.A., van Knippenberg, S.S.F.A., Biggins, L., To, S.K., et al. (2022). Integrated multi-omics reveal polycomb repressive complex 2 restricts human trophoblast induction. Nat. Cell Biol. *24*, 858–871. https://doi.org/10.1038/s41556-022-00932-w.

STAR★METHODS

KEY RESOURCES TABLE

REAGENT or RESOURCE	SOURCE	IDENTIFIER
**Chemicals, peptides, and recombinant proteins**		
Leukemia inhibitory factor (LIF)	This study; home-made	N/A
PD0325901	Axon Medchem	Cat#1408
CHIR99021	Axon Medchem	Cat#1386
Fetal bovine serum (FBS)	Gibco	Cat#10270-106
**Critical commercial assays**		
PureLink Genomic DNA Mini Kit	Invitrogen	Cat#K182001
iQ SYBR Green Supermix	Bio-Rad	Cat#1708885
Cell Culture DNA Midi Kit	Qiagen	Cat#13343
Complete protease inhibitor (PIC)	Roche	Cat#45-4693132001
DNA Degradase Plus	Zymo Research	Cat#E2021
GFP Nano trap beads	Chromotek	Cat#gta-20
BCA protein assay kit	Pierce	Cat#23225
Sera-Mag Speed Beads A, B	ThermoFisher	Cat#09-981-121, Cat#09-981-123
Qubit Protein Assay	Invitrogen	Cat#A50669
**Deposited data**		
WGBS data	This study	GEO: GSE299458
ChEP-MS data	This study	PRIDE: PXD074325
coIP MS data	This study	PRIDE: PXD074363
**Public ChIP-seq datasets**; See also Table S9		
ChIP-seq SetDB1, WT serum mouse ESCs	Warrier et al., (2022)	GEO: GSM3498570
ChIP-seq H3K9me3, WT serum mouse ESCs	Fisher et al., (2017)	GEO: GSM788388
ChIP-seq H3K9me3, *Setdb1* KO (D6) serum mouse ESCs	Fisher et al., (2017)	GEO: GSM788389
ChIP-seq H3K27me3, WT serum mouse ESCs	Fei et al., (2015)	GEO: GSM1708667
ChIP-seq H3K27me3, *Setdb1* KO (D3) serum mouse ESCs	Fei et al., (2015)	GEO: GSM1708670
ChIP-seq TET1, WT serum mouse ESCs	Williams et al., (2011)	GEO: GSM611192
ChIP-seq input (for GSM611192), WT serum ESCs	Williams et al., (2011)	GEO: GSM611207
ChIP-seq TET2, WT serum mouse ESCs	Xiong et al., (2016)	GEO: GSM2065691
ChIP-seq input (for GSM2065691), WT serum ESCs	Xiong et al., (2016)	GEO: GSM2065692
ChIP-seq CTCF, WT serum mouse ESCs	Tam et al., (2024)	GEO: GSM5589517
ChIP-seq CTCF, *Setdb1* KO (D6) serum mouse ESCs	Tam et al., (2024)	GEO: GSM5589518

(*Continued on next page*)

Continued

REAGENT or RESOURCE	SOURCE	IDENTIFIER
ChIP-seq H3K9me3, WT 2i mouse ESCs	Wu et al., (2020)	SRA: SRX5891227
ChIP-seq H3K9me3, *Setdb1* KO (D3) 2i mouse ESCs	Wu et al., (2020)	SRA: SRX5891223
ChIP-seq H3K27me3, WT 2i mouse ESCs	Wu et al., (2020)	SRA: SRX5891230
ChIP-seq H3K27me3, *Setdb1* KO (D3) 2i mouse ESCs	Wu et al., (2020)	SRA: SRX5891235
**Public RNA-seq datasets**; See also Table S11		
Bulk RNA-seq, WT serum mouse ESCs replicate 1	Barral et al., (2022)	GEO: GSM5233362
Bulk RNA-seq, WT serum mouse ESCs replicate 2	Barral et al., (2022)	GEO: GSM5233363
Bulk RNA-seq, WT serum mouse ESCs replicate 3	Barral et al., (2022)	GEO: GSM5233364
Bulk RNA-seq, *Setdb1* KO (D5) serum mouse ESCs replicate 1	Barral et al., (2022)	GEO: GSM5233365
Bulk RNA-seq, *Setdb1* KO (D5) serum mouse ESCs replicate 2	Barral et al., (2022)	GEO: GSM5233366
Bulk RNA-seq, *Setdb1* KO (D5) serum mouse ESCs replicate 3	Barral et al., (2022)	GEO: GSM5233367
Bulk RNA-seq, WT 2i mouse ESCs replicate 1	Wu et al., (2020)	SRA: SRX5891234
Bulk RNA-seq, WT 2i mouse ESCs replicate 2	Wu et al., (2020)	SRA: SRX5891239
Bulk RNA-seq, *Setdb1* KO (D5) 2i mouse ESCs replicate 1	Wu et al., (2020)	SRA: SRX5891241
Bulk RNA-seq, *Setdb1* KO (D5) 2i mouse ESCs replicate 2	Wu et al., (2020)	SRA: SRX5891248
**Experimental models: Cell lines**		
Setdb1$^{CKO/-}$ mouse ESCs (1)	Matsui et al., (2010)	N/A
Setdb1$^{CKO/-}$ mouse ESCs (2)	Fisher et al., (2017)	N/A
SetDB1-GFP HeLa BAC clone cells	Poser et al., (2008)	N/A
"Kyoto" wild-type HeLa cells	Poser et al., (2008)	N/A
**Oligonucleotides**		
IAP Hairpin bisulfite primer #1; TTTTTTTTTTAGGAGAGTTATATTT	This study	N/A
IAP Hairpin bisulfite primer #2; ATCACTCCCTAATTAACTACAAC	This study	N/A
qPCR primers; Table S6	This study	N/A
**Software and algorithms**		
Trim Galore	Krueger and Andrews, (2011)	https://github.com/FelixKrueger/TrimGalore
Bowtie2	Langmead and Salzberg, (2012)	http://bowtie-bio.sourceforge.net/bowtie2/index.shtml
Bismark	Krueger and Andrews, (2011)	https://github.com/FelixKrueger/Bismark
Seq2Science	van der Sande et al., (2023)	https://vanheeringen-lab.github.io/seq2science/
Macs2	Zhang et al., (2008)	https://pypi.org/project/MACS2/

(*Continued on next page*)

Continued

REAGENT or RESOURCE	SOURCE	IDENTIFIER
EaSeq	Lerdrup et al., (2016)	https://easeq.net
Gimme Maelstrom	Bruse and Heeringen, (2018)	https://simonvh.github.io/gimmemotifs-manuscript/
DESeq2	Love et al., (2014)	https://bioconductor.org/packages/release/bioc/html/DESeq2.html
DIA-NN	Demichev et al., (2020)	https://github.com/vdemichev/diann
DEP	N/A	https://rdrr.io/bioc/DEP/
Maarten's Retrieval System	N/A	https://github.com/cmbi/mrs
Clustal Omega	Sievers et al., (2011)	https://github.com/GSLBiotech/clustal-omega
JalView	Waterhouse et al., (2009)	https://gitlab.jalview.org/jalview/jalview
YASARA version Structure	Land and Humble, (2017)	https://www.yasara.org
WHAT IF	N/A	https://swift.cmbi.umcn.nl/servers/html/index.html
ChIPseeker	Yu et al., (2015)	https://github.com/YuLab-SMU/ChIPseeker
**Other**		
Genomic locations of ICRs; Table S8	This study	N/A

EXPERIMENTAL MODEL AND STUDY PARTICIPANT DETAILS

Cell culture of mouse ESCs

The *Setdb1*$^{CKO/-}$ mouse ESCs used throughout the study have been described in Matsui et al. (2010), and the alternative *Setdb1*$^{CKO/-}$ mouse ESCs in Fisher et al. (2017). ESCs were maintained in a humidified incubator at 37°C and 5% CO_2, and routinely tested negative for mycoplasma. Serum ESCs were cultured on 0.1% gelatin-coated culture plates in DMEM-Glutamax basal medium (Gibco) supplemented with 0.1 mM non-essential amino acids (Gibco), 1 mM sodium pyruvate (Gibco), 0.1 mM 2-mercaptoethanol, 5×10^5 U leukemia inhibitory factor (LIF) (home-made), 10% fetal bovine serum (FBS) (Gibco) and 25 U/mL Penstrep (Gibco). Serum ESCs were passaged every 3–4 days or when ~80% confluent in a 1:1-15 ratio (lower ratio after *Setdb1* KO induction). Thereto, attached cells were washed once with DPBS, and incubated with Trypsin-EDTA (Gibco) for 5 min in a humidified incubator. Trypsin was inactivated with an excess of FBS-containing medium. Dissociated cells were pelleted for 5 min at 300xg, and transferred to new plates as single cells. Serum *Setdb1*$^{CKO/-}$ mouse ESCs were converted to 2i conditions for >2 weeks.

2i ESCs were maintained on 0.2% gelatin-coated culture plates in Ndiff 227 medium (Takara) supplemented with 1 μM MEK inhibitor PD0325901 (Axon Medchem), 3 μM GSK3 inhibitor CHIR99021 (Axon Medchem), together known as '2i' (Ying et al., 2008), 5×10^5 U LIF (home-made) and 25 U/mL Penstrep (Gibco). 2i ESCs were passaged every 2–3 days in a 1:1-12 ratio (lower ratio after *Setdb1* KO induction). Briefly, attached cells were washed once with DPBS, and incubated with accutase (StemPro) for 3–5 min in a humidified incubator, until visually detached. Dissociated cells were pelleted for 5 min at 300xg, dissolved in 2i medium, and transferred to new plates as single cells.

Setdb1 KO was induced by supplementing the culture medium with 800 nM 4-hydroxytamoxifen (4-OHT; prepared from a 1 mM stock in ethanol) for 4 days, as described by Matsui et al. (2010).

Cell culture of HeLa cells

SetDB1-GFP stable BAC clone HeLa cells have been described in Poser et al. (2008). The BAC clone and "Kyoto" WT HeLa cells were grown on tissue culture treated plates in DMEM-Glutamax basal medium (Gibco) supplemented with 0.1 mM non-essential amino acids (Gibco), 10% FBS (Gibco) and 25 U/mL Penstrep (Gibco). HeLa cells were passaged every 3–4 days or when confluent in a 1:10 ratio. Thereto, attached cells were washed once with DPBS, and incubated with Trypsin-EDTA (Gibco) for 5 min in a humidified incubator. Trypsin was inactivated with an excess of FBS-containing medium. Dissociated cells were pelleted for 5 min at 300xg, and transferred to new plates as single cells.

METHOD DETAILS

Quantitative PCR

Quantitative PCR (qPCR) was performed on extracted genomic DNA from ESCs and used as a template for amplification. Genomic DNA was isolated using a PureLink Genomic DNA Mini Kit (Invitrogen) according to the manufacturer's instructions. qPCR was then conducted with iQ SYBR Green Supermix (Bio-Rad) on a CFX96 Real-Time system (Bio-Rad). The DNA input was normalized to the *Act*, *Rex* and *Setdb1* exon 7 genomic loci. Primer sequences are provided in Table S6.

DNAme measurements by MS

DNA measurements by MS were performed as previously described (Habibi et al., 2013). Briefly, DNA was extracted from ESCs using the PureLink Genomic DNA Mini Kit (Invitrogen) following the manufacturer's instructions. The samples were diluted to a final DNA concentration of 100 ng/μL, with a minimum volume of 3 μL. DNA degradation into individual nucleosides was achieved using DNA Degradase Plus (Zymo Research). These nucleosides were analyzed using liquid chromatography-mass spectrometry (LC-MS) on an Exploris 480 platform (ThermoFisher). Quantification was performed based on a standard curve generated from calibration standards. The levels of 5mC and 5hmC were expressed as percentage concentration ratios: % 5-methyl-2′-deoxycytidine to 2′-deoxyguanosine (%mdC/dG) and % 5-hydroxymethyl-2′-deoxycytidine to 2′-deoxyguanosine (%5hmdC/dG), respectively.

Hairpin bisulfite sequencing and data processing

Hairpin bisulfite sequencing was performed as described in Arand et al. (2012), with minor adjustments, similar to Habibi et al. (2013). Briefly, BsaWI was used as restriction enzyme of L1. PCR amplification was performed as follows: 40 cycles of 60 s at 95°C for denaturation, 90 s at 51°C for annealing, and 60 s at 72°C for extension. Sequencing of PCR amplicons was performed on either a GS FLX Titanium or Illumina MiSeq platform. Read mapping and calculation of cytosine methylation levels were performed as previously described in Habibi et al. (2013).

Whole-genome bisulfite sequencing and data processing

DNA from ESCs was isolated using a Cell Culture DNA Midi Kit (Qiagen), following the manufacturer's instructions. WGBS on isolated DNA was conducted as previously described (Kulis et al., 2012). Libraries were sequenced on an Illumina HiSeq 2000 platform in paired-end mode (2×100 nucleotides). All WGBS (and other sequencing) analyses were performed using the *Mus musculus* NCBI GRCm39 genome assembly (mm39). Paired-end reads from the FASTQ files were trimmed using Trim Galore (Krueger and Andrews, 2011) with the flags *–paired –trim1*. The trimmed reads were then aligned to the reference genome using the Bismark package (Krueger and Andrews, 2011) in combination with Bowtie 2 (Langmead and Salzberg, 2012) with the flag *-n 1 –bowtie2*. BAM files were deduplicated using Deduplicate Bismark with the flag *-p*. Cytosine methylation levels were calculated using the Bismark Methylation Extractor with the flags *-p –no_overlap* and applying a minimum read coverage threshold of ≥5 (Krueger and Andrews, 2011). The fraction of CpGs that were sequenced with ≥5 coverage, and the mean CpG coverage are summarized in Table S7. Methylation levels of genomic features were determined by intersecting them with genomic coordinates of the mm39 reference genome, including bins (see below), ChIP-seq target regions, loci of genes, repeats and ICRs (Table S8). Repeat locations were obtained from the UCSC mm39 Repeatmask dataset.

Binning of whole-genome bisulfite sequencing data

The DNAme output data generated by Bismark, as described above, were partitioned into fixed 10kb genomic bins, a similar scale as used previously for SetDB1 peak-centered analyses (Fei et al., 2015). Binning was performed in R. Briefly, for each chromosome, the dataset was first converted into a *data.table* object to enable efficient subsetting. Starting from the minimum genomic coordinate present in each chromosome, consecutive 10kb windows were scanned until the maximum coordinate of that chromosome was reached. Within each 10kb window, all sequenced CpGs with start and end positions within the bin (Start ≥ i and End < i + 10,000) were extracted. Bins with >25 sequenced CpGs, of which >10 were sequenced in all samples, were retained for downstream processing. For each retained bin, the mean DNAme level was calculated across all time points using *colMeans* with *na.rm = TRUE*. Bins were accumulated into a final genome-wide dataset. Empty rows generated during matrix initialization were removed using *na.omit*.

ChIP-seq data processing

SetDB1, H3K9me3, H3K27me3, TET1, TET2, and CTCF ChIP-seq datasets were obtained from previous studies (Table S9). ChIP-seq FASTQs were processed with our in-house Seq2Science package (van der Sande et al., 2023) using the mm39 reference genome and custom ChIP-seq settings (Table S10) to allow read mapping against repetitive regions. Seq2Science's Macs2 (Zhang et al., 2008) output provided the genomic ranges of peaks along with TMM-normalized values, which served as an approximation of peak sizes. To calculate RPKM values for each genomic feature discussed in this article, the BAM files generated by Seq2Science were imported into EaSeq (Lerdrup et al., 2016). The standard ChIP-seq import settings were used, except "*only allow reads with unique positions*" was disabled. In addition to intersecting the ChIP-seq data with methylome bins and SetDB1 peak regions, the data were directly mapped to the chromosomal locations of genes, repeats and ICRs. RPKM values for genomic features were calculated as follows: (1) The number of overlapping DNA fragments from ChIP-targets were counted within an area ranging from the start of the regions with an offset of 0bp, to end of the regions with an offset of 0bp. (2) Quantities were normalized to a dataset size of one million reads. (3) The number of fragments was derived from the count by dividing it with (1 + DNA fragment size/size of the area). The RPKM values were subsequently tabulated and exported from EaSeq as tab-separated TXT-files and further processed, analyzed and visualized in R.

Transcription factor motif analysis

Transcription factor binding motifs were identified within SetDB1 ChIP-seq peaks using Gimme Maelstrom and the non-redundant Gimme Vertebrate motif database to maximize the number of detectable motifs (Bruse and Heeringen, 2018). Briefly, SetDB1 peaks were standardized to 200 base pairs centered on their summits, and the number of reads in the standardized peak regions were log transformed and quantile normalized. Maelstrom applies an ensemble learning approach with eight different methods combined using a rank aggregation, in order to generate motif activity Z-scores, as displayed throughout the manuscript. The list of transcription factor binding motifs was further curated by retaining motifs with an activity exceeding one standard deviation above the mean, while excluding duplicates, unknown transcription factors and transcription factors without mouse orthologs. Full and curated lists are available as Table S1.

RNA-seq data processing

RNA-seq datasets were obtained from previous studies (Table S11). Similar to ChIP-seq data, RNA-seq FASTQs were processed with our in-house Seq2Science package (van der Sande et al., 2023) using the mm39 reference genome. The reference genome pulled by Seq2Science was supplemented through *-custom_annotation_extension* with a BED-like GTF-file containing the chromosomal locations of all repeats in the UCSC mm39 Repeatmask dataset. Custom RNA-seq settings were applied to map reads against genes and repetitive elements, as previously reported (Teissandier et al., 2019), and summarized in Table S12. The Seq2Science read count tables were filtered by omitting genes and repetitive elements with less than 3 reads. Fold-change values (WT vs. *Setdb1* KO) were generated using the DESeq2 package (Love et al., 2014), and further processed, analyzed and visualized in R.

Nuclear isolation from HeLa cells and SetDB1 co-immunoprecipitation followed by MS

Nuclear extracts were prepared from HeLa cells, i.e., SetDB1-GFP stable BAC-clone and WT, for performing co-immunoprecipitation of SetDB1-GFP. Thereto, cells were expanded in 10 × 15 cm dishes per cell line. After growing to confluency, cells were dissociated as described previously, and collected in 50 mL tubes. Dissociated cells were pelleted at 4°C for 5 min at 400xg. Cells were washed with ice-cold DPBS, combined into one 50 mL tube, and pelleted at 4°C for 5 min at 400xg. Cells were washed once more with ice-cold DPBS, transferred to one 15 mL tube, and pelleted at 4°C for 5 min at 400xg again. Cell pellets were gently resuspended in 5 pellet-volumes of ice-cold Buffer A (10 mM HEPES KOH pH 7.9, 1.5 mM $MgCl_2$, 10 mM KCl) using a p1000 pipette, and incubated for 10 min on ice. Cells were pelleted at 4°C for 5 min at 400xg. The volume of the cell pellet was subsequently determined (cell volume will increase due to osmotic uptake of liquid by cells), after which 2 volumes of ice-cold Buffer A supplemented with complete protease inhibitor (PIC) (Roche) and 0.15% NP40 were added. Cells were gently resuspended and transferred to glass dounce homogenizers placed on ice. Cells were lysed to release nuclei by performing 4 × 10 strokes with a type-B pestle (tight). After each set of 10 strokes, cell suspensions were rested on ice for 2 min to limit heating. Suspensions were then transferred to 15 mL tubes, after which nuclei were pelleted at 4°C for 15 min at 3200xg. Supernatants, i.e., cytoplasmic fractions, were then removed from nuclear pellets. Nuclear pellets were cleaned by rinsing (without resuspension) once with 10 pellet-volumes of ice-cold DPBS, followed by centrifugation at 4°C for 5 min at 3200xg. The obtained pellets are crude nuclei. The supernatant was completely removed by pouring, followed by inversion of tubes on paper towel. The volume of the nuclear pellet was determined, and

nuclei were resuspended in 2 pellet-volumes Buffer C.1 (420 mM NaCl, 20 mM HEPES KOH pH 7.9, 20% v/v glycerol, 2 mM $MgCl_2$, 0.2 mM EDTA, 0.1% NP40, PIC, and 0.5 mM DTT; last three components added fresh before use). Suspensions were rotated in a 4°C cold-room for 1 h. Due to the lysis of nuclei in Buffer C.1 and the release of chromatin, the suspension will turn viscous. Nuclear suspensions were then centrifuged at 4°C for 30 min at 14,000rpm in a table top centrifuge. The supernatants, i.e., nuclear extracts, were aliquoted and snap-frozen in liquid nitrogen, and stored at −80°C.

For the pull-down, frozen nuclear extracts (−80°C) were quickly thawed and placed on ice. GFP Nano trap beads (Chromotek) (15 μL per pull-down sample, transferred with cut pipette tip) were washed twice with 1 mL DPBS and once with 1 mL Buffer C.2 (300 mM NaCl, 20 mM HEPES KOH pH 7.9, 20% v/v glycerol, 2 mM $MgCl_2$, 0.2 mM EDTA, 0.25% NP40, PIC, and 0.5 mM DTT; last three components added fresh before use), using a p1000 pipette for resuspension and centrifugation at 2000xg for 2 min at RT. The beads were resuspended in Buffer C.2, and divided equally across individual tubes for each pull-down sample/replicate. The protein concentrations of the nuclear extracts were assessed with a BCA protein assay kit (ThermoFisher), and 750 μg of nuclear protein per replicate was added to the aliquoted beads. Each sample was supplemented with 2 μL ethidium bromide (10 mg/mL), after which Buffer C.2 was added to 400 μL volume per replicate. Pull-down samples were incubated on a rotating wheel in a 4°C cold room.

Incubated beads were washed twice with 1 mL Buffer C.3 (300 mM NaCl, 20 mM HEPES KOH pH 7.9, 20% v/v glycerol, 2 mM $MgCl_2$, 0.2 mM EDTA, 0.5% NP40, PIC, and 0.5 mM DTT; last three components added fresh before use), and twice with 1 mL of DPBS without NP40. Beads were dried by aspirating the supernatant with an insulin syringe. Beads were incubated with 50 μL elution buffer (EB) (made fresh: 120 urea dissolved in 100 μL Tris pH 8, topped-up to 1 mL, supplemented with 10 mM DTT) for 20 min at 25°C on a shaking heating block. Then, 5 μL 0.55 mM IAA was added, after which beads were incubated for 5 min at 25°C in the dark. Samples were digested upon supplementation with 2.5 μL trypsin (Promega) and incubation for 2 h at 25°C on a shaking heating block. Supernatants (i.e., peptides) were stored on ice, and beads were incubated with an additional 50 μL elution buffer (EB), the supernatant of which was later added to the previous supernatant.

Peptides were desalted and concentrated by stage-tipping. Briefly, C18 stage tips were flushed once with 50 μL methanol (centrifuged at 4000rpm for 2 min at RT on a table top centrifuge). Stage tips were then flushed once with Eluent B (80% acetonitrile, 0.1% TFA in water), and twice with Eluent A (0.1% TFA in water) in similar fashion. Samples with eluted peptides were then added to stage tips, and subsequently centrifuged at 2400rpm for 10 min at RT on a table top centrifuge (slower speed so that peptides can bind C18 column). Stage tip columns were flushed once with Eluent A, after which samples were eluted from the stage tips in 12 μL Eluent B. Acetonitrile was removed from the samples using a SpeedVac for 15 min. Eluent A was then added to a final volume of 20 μL, after which samples were analyzed by liquid chromatography-mass spectrometry on an Astral MS platform (ThermoFisher).

ChEP-MS

ChEP-MS was performed as previously described, with minor adjustments (Kustatscher et al., 2014; Zijlmans et al., 2022). Briefly, 1×10^7 cells were crosslinked with 1% formaldehyde for 10 min at RT, and then quenched with 0.25 M glycine. Cells were resuspended in 1 mL ice-cold cell lysis buffer (25 mM Tris pH 7.4, 0.1% Triton X-100, 85 mM KCl, and PIC) and centrifuged at 2,300xg for 5 min at 4°C. The supernatant (cytoplasmic fraction) was removed, and pellets were resuspended in 500 μL SDS buffer (50 mM Tris pH 7.4, 10 mM EDTA, 4% SDS, and PIC) and incubated for 10 min at RT. The volume was brought to 2 mL with urea buffer (10 mM Tris pH 7.4, 1 mM EDTA, 8 M urea), followed by centrifugation at 16,100xg for 30 min at RT. The supernatant was discarded, after which the previous wash step was repeated once. Pellets were then resuspended in 500 μL SDS buffer, topped-up to 2 mL with SDS buffer, and centrifuged again at 16,100xg for 30 min at RT. After removing the supernatant, the remaining protein pellet was resuspended in 250 μL storage buffer (10 mM Tris pH 7.4, 1 mM EDTA, 25 mM NaCl, 10% glycerol, and PIC) and sonicated using an NGS Bioruptor (Diagenode) for five cycles (30 s on/30s off) to solubilize the material. Protein concentrations were then measured using a Qubit Protein Assay (Invitrogen). For MS sample preparation, 10 μg of extract was de-crosslinked at 95°C for 30 min. Each ChEP condition was analyzed in three biological replicates.

De-crosslinked chromatin protein extracts (10 μg) were precipitated with 10 volumes of ice-cold 100% acetone at −20°C for 15 min and centrifuged at 16,000xg for 10 min at 4°C. Samples were washed once with 500 μL ice-cold acetone, centrifuged, and the supernatant discarded. Proteins were resuspended in 100 μL urea buffer (100 mM urea, 100 mM Tris pH 8.0, 10 mM DTT) and incubated at RT for 20 min. Iodoacetamide (10 μL) was then added, and samples were incubated in the dark at RT for 15 min. Protein purification and on-bead digestion were performed by adding 5 μL prewashed Sera-Mag Speed Beads (ThermoFisher) and 154 μL binding buffer (100% acetonitrile) for 18 min at RT. The protein solution was

washed twice with 180 µL 70% ethanol and once with 200 µL 100% acetonitrile. Proteins were eluted and digested in 100 µL ammonium bicarbonate (ABC) with 0.5 µg trypsin (Promega) for 2 h at 37°C. Beads were removed, and digestion was prolonged overnight at 37°C. Peptides were desalted and concentrated by stage-tipping, as described above for co-immunoprecipitation, prior to analysis by LC-MS on an Astral platform (ThermoFisher).

Processing of coIP and ChEP MS data

Raw MS spectra were processed using DIA-NN software (version 1.9.2) (Demichev et al., 2020). The FASTA files used for the coIP experiment were the human proteome with included isoforms (UniProtKB ID UP000005640; downloaded April 2022) and a FASTA file containing common contaminants from the cRAP database (April 2019 release). Similarly, FASTA files for the ChEP experiment were the mouse proteome with included isoforms (UniProtKB ID UP000000589; downloaded June 2024) and cRAP FASTA file (April 2019 release). First, a spectral library was generated from the FASTA files using DIA-NN's library-free search workflow, with Trypsin/P specified as the protease. Cysteine carbamidomethylation, methionine oxidation, N-terminal methionine excision, and N-terminal acetylation were included as modifications, with a maximum of five variable modifications per peptide, and up to two missed cleavages. The spectral library and FASTA files were then used for the main search. Mass accuracy and MS1 accuracy were set to 10 and 4 ppm, respectively, and match-between-runs was enabled. For the coIP samples, the optimal scan window was determined by analyzing the samples as unrelated runs prior to the main search. All other parameters remained at default settings.

Downstream processing of the DIA-NN output files was performed in R. Quantified proteins with more than two unique peptides in all three replicates of at least one experimental group were retained. Proteins annotated as contaminants, keratins, or cRAP entries were filtered out. Normalization was done using VSN (variance stabilizing normalization) (Huber et al., 2002). Missing values were imputed for statistical analysis using the "MinProb" method with default settings. Differential protein analysis was performed using DEP, which uses a Student's t test. Volcano plots were generated by plotting the -$\log_{10}$ of the adjusted *p*-value against the $\log_2$ fold-change of the differentially expressed proteins.

AlphaFold 3 multimer model predictions

The primary sequence of human and mouse SetDB1 (AC: Q15047, O88974, respectively), human HP1α (AC: P45973), mouse sequences of DNMT1 (AC: P13864), DNMT3a (AC: O88508), DNMT3b (AC: O88509), EZH2 (AC: Q61188), SUZ12 (AC: Q80U70), CTCF (AC: Q61164), SMC1a (AC: Q9CU62), SMC3 (AC: Q9CW03) and SMAD3 (AC: Q8BUN5) were obtained from the UniProt database. The BLAST function (Altschul et al., 1990) in Maarten's Retrieval System (MRS) was used to obtain the percentage identity and similarity between human and mouse SetDB1 (Hekkelman and Vriend, 2005). Multiple sequence alignments (MSA) were performed three times for SetDB1 and candidate interactors by Clustal Omega (Sievers et al., 2011) with default settings (Madeira et al., 2024). The MSAs were loaded into JalView.

AlphaFold3 was used to generate protein-protein interaction predictions. The protocol and evaluation were based on methods described by Tang et al. (2025). The predicted pairwise alignment error (PAE) in units angstrom (Å) indicates the uncertainty in the predicted positions between any two residue pairs, and was used to identify interacting domains. AlphaFold3 runs were conducted with residues of mouse SetDB1 and full-length sequences of the candidate interactors. The sequences of the SetDB1 domains and that of the interactors were provided to the AlphaFold3 webserver with default settings. The mouse SetDB1 protein was subdivided into the following domains and regions: TTD (aa 201–395), Flex1 (aa 396–610), MBD (aa 611–682), and Flex2 (aa 683–1307). All subsequent analyses were performed on the top-ranked model (model 0), as determined by AlphaFold3. Yet Another Scientific Artificial Reality Application (YASARA) version Structure and WHAT IF were used to visualize the models (Krieger and Vriend, 2014, 2015). The chain ID and residue numbers obtained from the AlphaFold3 runs were used to retrieve the native residue numbering. Extracted PAE values were visualized using a Seaborn heatmap (Waskom, 2021), and regions with a PAE value below five were identified as potential interaction sites.

QUANTIFICATION AND STATISTICAL ANALYSIS

Replication and statistical procedures

Data throughout manuscript are presented as means, unless otherwise stated in the figure legends or on axis titles. Boxplots displayed throughout manuscript indicate medians and quantiles, as well as means (white diamonds) and expected counts (red diamonds). Grouped data throughout manuscript are accompanied by their group sizes in percentages and/or total numbers, as stated in figures, legends and main text. Heatmap clustering was performed using the Ward-D method. The RNA-seq data are presented as $\log_2$ fold-changes (*Setdb1* KO vs. WT ESCs), accompanied with *p*-values

calculated using a Wald test by DESeq2. Significant differential genes were defined by $\log_2$ fold-change ≥ 1 and -$\log_{10}$ p-value ≥ 1 throughout the manuscript. Peak calling in ChIP-seq data was performed with our in-house Seq2Science package (van der Sande et al., 2023), using Macs2. The Macs2 package first uses a dynamic local lambda (λlocal) for each candidate peak, and a Poisson distribution to calculate a p-value for each region based on λlocal. Regions with p-values $\leq 10^{-5}$ are considered enriched (i.e., "a peak"). Overlapping peaks were merged into larger peaks. Peak summits were determined as coordinates within peaks with the highest read pile-up. GO enrichment analyses were based on Over Representation Analysis (ORA) of terms, and are accompanied by p-values of individual terms, calculated using a hypergeometric distribution. Adjusted p-values were subsequently calculated using the Benjamini-Hochberg (BH) method. Figures related to GO enrichments display groups of varying sizes with the top most significant terms. The significance of motifs identified by Gimme Maelstrom are displayed as Z-scores throughout the manuscript. This Z-score was calculated from the combined result of multiple statistical methods, as mentioned in Bruse and Heeringen (2018). The results from the individual methods are ranked from high-scoring motifs to low-scoring motifs, and converted to Z-scores using an inverse normal transformation. The Z-scores from individual methods are then combined using Stouffer's method. The scores that are shown are therefore aggregated Z-scores. A higher Z-score reflects a stronger presence of that motif in a specific sample category. Bins and peaks were annotated to genomics elements with transcription start sites defined as −3000 to 3000 bp of genic loci, using ChIPseeker, with an annotation priority of Promoter, 5′ UTR, 3′ UTR, Exon, Intron, Downstream, Intergenic in case overlaps with multiple elements were detected. ChEP-MS data are presented as bar plots of DIA-NN normalized (mean) peptide counts for individual proteins where dots represent individual values of replicates (n = 3) and error bars indicate the standard error from the mean, or as volcano plots showing $\log_2$ fold-changes (*Setdb1* KO vs. WT ESCs) and corresponding -$\log_{10}$ adjusted p-values. Significant differential proteins among ChEP-MS samples were defined by a $\log_2$ fold-change ≥ 1 and -$\log_{10}$ p-adjusted ≥ 1. The coIP MS data are presented as volcano plots with $\log_2$ fold-changes (SetDB1 BAC clone HeLa vs. WT HeLa), accompanied with -$\log_{10}$ adjusted p-values. Significantly enriched proteins in the coIP MS experiment were defined by a $\log_2$ fold-change ≥ 1 and -$\log_{10}$ p-adjusted ≥ 1.

Stem Cell Reports, Volume *21*

Supplemental Information

Methylome profiling of SetDB1-deficient ESCs reveals coordinated epigenetic cross-talk during pluripotency

Nick G.P. Bovee, Ehsan Habibi, Zicong Liu, Stefan H.A. Hoogland, Siebren Frölich, Daniëlle Diepenbroek, Kim C.M. Bosman, Julia Arand, Irina A. Maksakova, Anouk Klein Kranenbarg, Jörn Walter, Arie B. Brinkman, Klaas W. Mulder, Joop H. Jansen, Michiel Vermeulen, Matthew Lorincz, Hendrik G. Stunnenberg, and Hendrik Marks

Supplemental figures

Figure S1

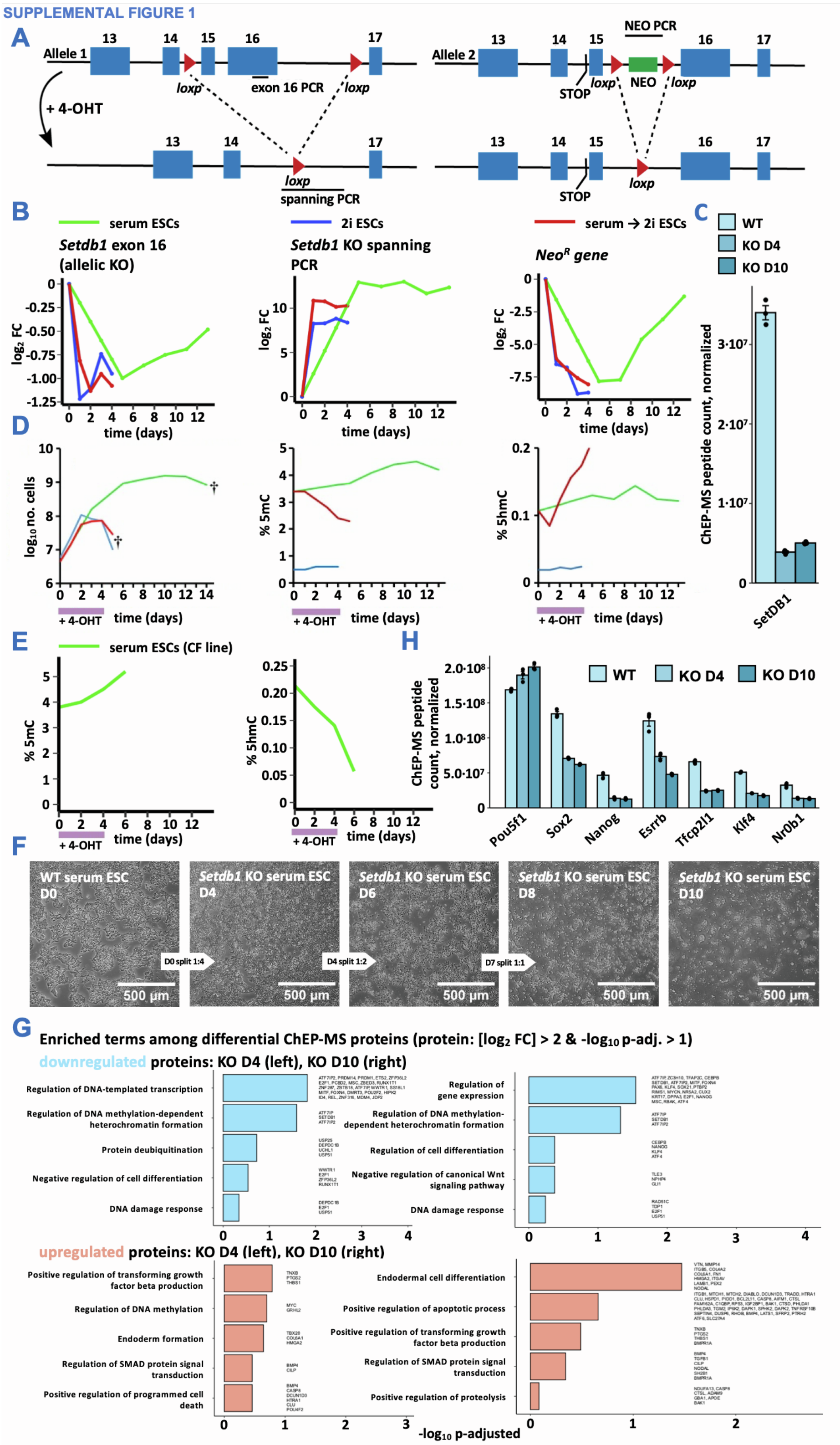

Figure S1: Loss of SetDB1 induces global DNAme increase, differentiation, induction of cell death and change of chromatin-proteome, related to Main Figure 1-4, and Supplemental Figure 2. A, Schematic representation of *Setdb1* KO strategy. Left side illustrates the conditional *Setdb1* KO strategy by exon deletion through Cre-lox recombination upon 4-OHT treatment. Right side illustrates the simultaneous deletion of a neomycin resistance gene (NEO) upon 4-OHT treatment on the other allele. **B**, qPCR data confirming functionality of 4-OHT treatment to induce *Setdb1* KO showing deletion of *Setdb1* exon 15 and 16 and removal of the NEO gene. **C**, Bar graph, similar to Fig. 3F, showing KO of SetDB1 at protein level. **D**, Line graphs of cell count (left), global 5mC (middle) and global 5hmC (right) upon induction of *Setdb1* KO in serum ESCs (green line) and 2i ESCs (blue line). 5(h)mC levels were determined by mass spectrometry. Red lines represent data of *Setdb1* KO ESCs undergoing simultaneous transition from serum to 2i culture. **E**, Line graphs similar to those of panel D (middle and right panel). Data collected from alternative $Setdb1^{CKO/-}$ ESC “CF line”. **F**, Microscopic images of WT and *Setdb1* KO serum ESCs on different time points post *Setdb1* KO induction. **G**, Curated lists of enriched biological processes from GO-analyses, shown as bar graphs, among differential proteins measured by ChEP-MS, defined by an absolute $\log_2$ fold-change > 2 and $-\log_{10}$ p-adjusted value > 1. Proteins related to terms indicated behind bars. Horizontal axis represents $-\log_{10}$ p-adjusted value of terms. **H**, Bar graph similar to Fig. 3F, of proteins involved in pluripotency network.

Figure S2

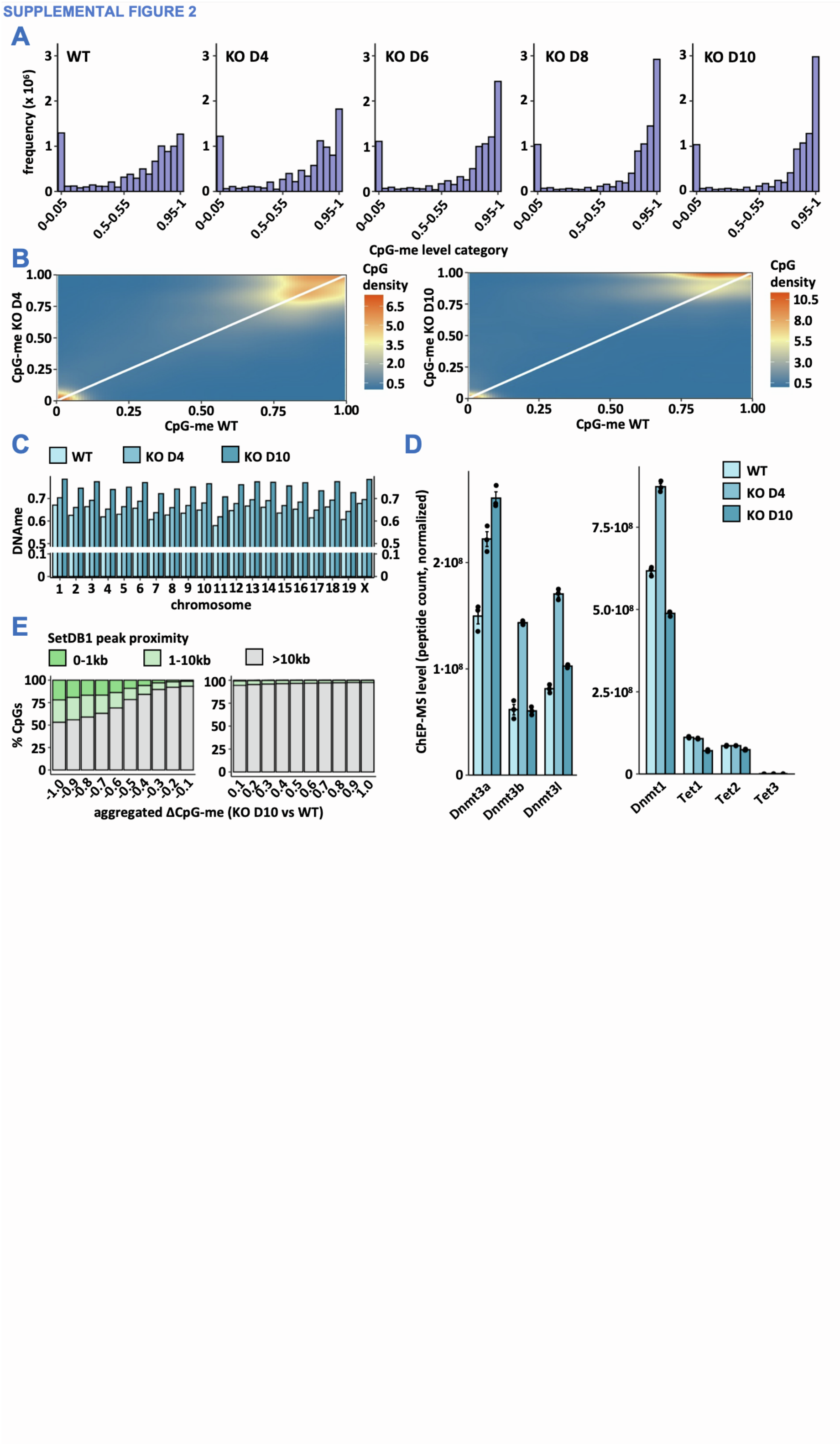

SUPPLEMENTAL FIGURE 2
A
WT
KO D4
KO D6
KO D8
KO D10
frequency (x 10⁶)
0-0.05
0.5-0.55
0.95-1
CpG-me level category
B
CpG-me KO D4
CpG-me WT
CpG density
6.5
5.0
3.5
2.0
0.5
CpG-me KO D10
CpG density
10.5
8.0
5.5
3.0
0.5
C
WT
KO D4
KO D10
DNAme
chromosome
D
ChEP-MS level (peptide count, normalized)
Dnmt3a
Dnmt3b
Dnmt3l
Dnmt1
Tet1
Tet2
Tet3
E
SetDB1 peak proximity
0-1kb
1-10kb
>10kb
% CpGs
aggregated ΔCpG-me (KO D10 vs WT)

Figure S2: Loss of SetDB1 induces global DNAme increase and demethylation near specific SetDB1 targets, as observed by WGBS time course, related to Main Figure 1-2, and Supplemental Figure 1. A, Bar graph of CpG methylation levels in WT and *Setdb1* KO serum ESCs, as in Fig. 1B. **B**, Heatmap showing methylation remodeling of individual CpGs upon *Setdb1* KO day 4 and day 10, relative to WT serum ESCs, as in Fig. 1E. **C**, Bar graph showing average methylation of individual chromosomes in WT and *Setdb1* KO serum ESCs (WT, day 4 and day 10). **D**, Bar graph similar to Fig. 3F, of proteins involved in deposition and enzymatic removal of DNAme. **E**, Bar graph illustrating the percentage of proximally SetDB1-bound CpGs (categorized as 0-1kb, 1-10kb and > 10kb distance between CpG and SetDB1 peak). CpGs were split in demethylated (left) and increasingly methylated (right) groups and subsequently ranked by aggregated ∆DNAme intervals on the horizontal axis.

Figure S3

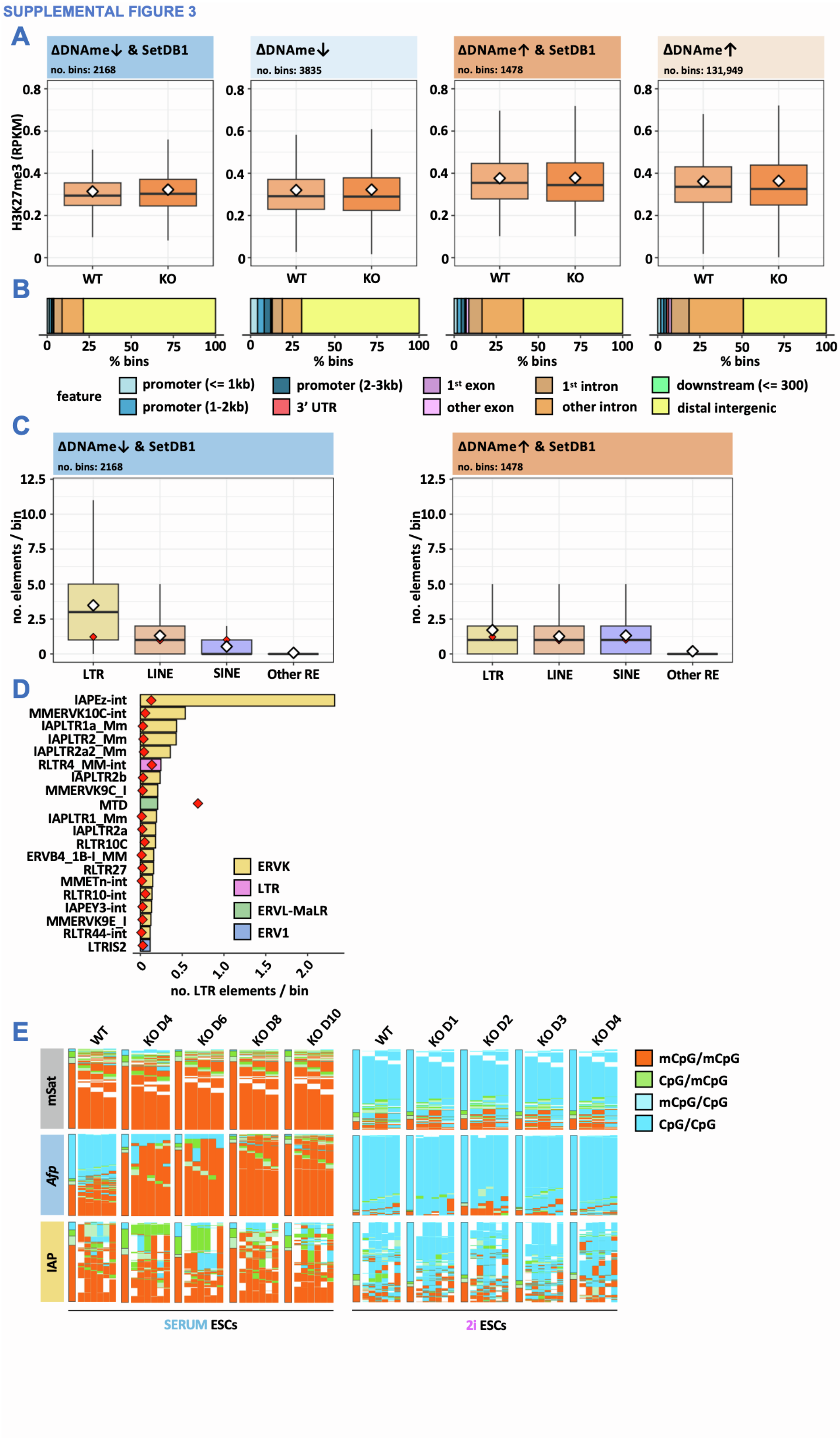

SUPPLEMENTAL FIGURE 3
A
ΔDNAme↓ & SetDB1
no. bins: 2168
ΔDNAme↓
no. bins: 3835
ΔDNAme↑ & SetDB1
no. bins: 1478
ΔDNAme↑
no. bins: 131,949
H3K27me3 (RPKM)
WT
KO
B
% bins
feature
promoter (<= 1kb)
promoter (1-2kb)
promoter (2-3kb)
3' UTR
1st exon
other exon
1st intron
other intron
downstream (<= 300)
distal intergenic
C
ΔDNAme↓ & SetDB1
no. bins: 2168
ΔDNAme↑ & SetDB1
no. bins: 1478
no. elements / bin
LTR
LINE
SINE
Other RE
D
IAPEz-int
MMERVK10C-int
IAPLTR1a_Mm
IAPLTR2_Mm
IAPLTR2a2_Mm
RLTR4_MM-int
IAPLTR2b
MMERVK9C_I
MTD
IAPLTR1_Mm
IAPLTR2a
RLTR10C
ERVB4_1B-I_MM
RLTR27
MMETn-int
RLTR10-int
IAPEY3-int
MMERVK9E_I
RLTR44-int
LTRIS2
ERVK
LTR
ERVL-MaLR
ERV1
no. LTR elements / bin
E
WT
KO D4
KO D6
KO D8
KO D10
WT
KO D1
KO D2
KO D3
KO D4
mSat
Afp
IAP
mCpG/mCpG
CpG/mCpG
mCpG/CpG
CpG/CpG
SERUM ESCs
2i ESCs

Figure S3: Targets with SetDB1-dependent DNAme and H3K9me3 are enriched with ERVKs, related to Main Figure 2 & 4. A, Boxplots of H3K27me3 levels, as in Fig. 2A. **B**, Bar plot showing the overlapping genomic features at methylome bin centers, as grouped in Fig. 2A. Genomic annotations determined by ChIPseeker. **C**, Boxplots showing the number of overlapping repeat elements per methylome bin from the SetDB1-targeted ∆DNAme↓ and ∆DNAme↑ bin groups. The other RE group includes scRNAs, rRNAs, snRNAs, srpRNAs, DNA repeats, satellites, RCs, Retroposons and tRNAs. White diamonds represent observed averages. Expected averages in any 10kb bin plotted as red diamonds. **D**, Bar graph of observed and expected frequency (red diamonds) of the 20-most-occurring LTRs in SetDB1-targeted ∆DNAme↓ bins. **E**, Heatmap representation of hairpin bisulfite sequencing data collected from WT and *SetDB1* KO serum and 2i ESCs, showing SetDB1 targeted IAPs, and control loci not targeted by SetDB1. Control loci include the *Afp* genic locus that shows an increase in DNAme following *Setdb1* KO in serum ESCs, and major satellite repeats (mSat), which remain hypermethylated in both WT and KO conditions in serum ESCs.

Figure S4

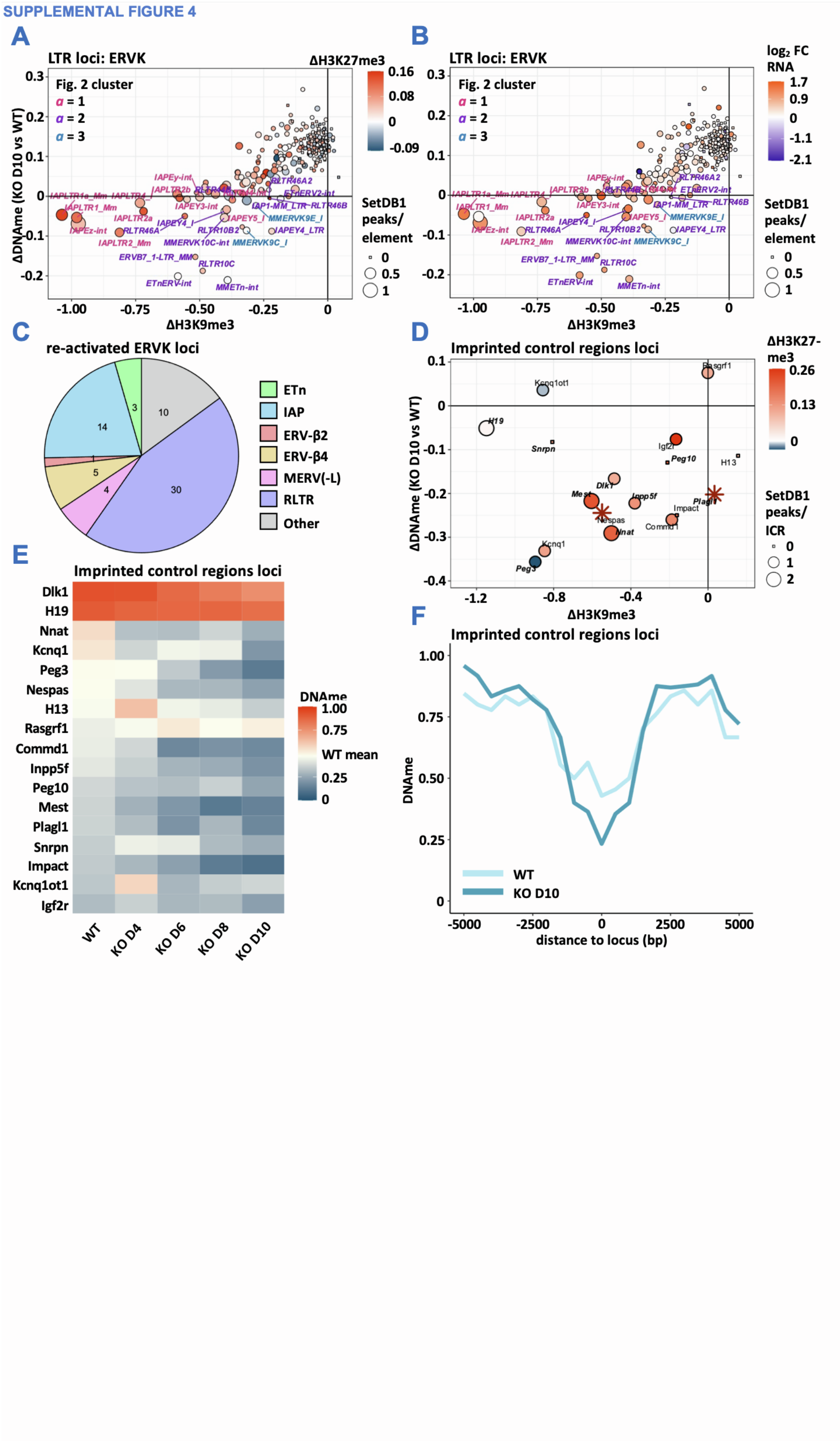
SUPPLEMENTAL FIGURE 4
A
LTR loci: ERVK
Fig. 2 cluster
α = 1
α = 2
α = 3
ΔH3K27me3
0.16
0.08
0
-0.09
SetDB1 peaks/ element
0
0.5
1
ΔDNAme (KO D10 vs WT)
ΔH3K9me3
B
LTR loci: ERVK
log2 FC RNA
1.7
0.9
0
-1.1
-2.1
C
re-activated ERVK loci
ETn
IAP
ERV-β2
ERV-β4
MERV(-L)
RLTR
Other
D
Imprinted control regions loci
ΔH3K27-me3
0.26
0.13
0
SetDB1 peaks/ ICR
E
Imprinted control regions loci
Dlk1
H19
Nnat
Kcnq1
Peg3
Nespas
H13
Rasgrf1
Commd1
Inpp5f
Peg10
Mest
Plagl1
Snrpn
Impact
Kcnq1ot1
Igf2r
WT
KO D4
KO D6
KO D8
KO D10
DNAme
1.00
0.75
WT mean
0.25
0
F
Imprinted control regions loci
DNAme
distance to locus (bp)
WT
KO D10

Figure S4: **ERVKs and ICRs with SetDB1-dependent DNAme-H3K9me3 are reactivated in serum ESCs following SetDB1 loss, related to Main Figure 2, and Supplemental Figure 5-6. A**, Scatter plot of LTR-ERVKs showing ΔH3K9me3 (ΔRPKM, KO vs WT), ΔDNAme (KO day 10 vs WT), and ΔH3K27me3 (ΔRPKM, KO vs WT) of ERVKs in serum ESCs upon *Setdb1* KO. Number of SetDB1 ChIP-seq peaks per ERVK copy (1kb proximity) displayed as point size. Color of name tag reflects clusters of Fig. 2B in which ERVK is predominantly found. **B**, Scatter plot similar to panel A, but color scale now represents difference of expression after *Setdb1* KO, as $\log_2$ fold-change. **C**, Pie chart illustrating the number of increasingly expressed ERVKs upon *Setdb1* KO in serum ESCs from different ERVK-groups (fold-change > 1.5). **D**, Scatter plot of ICRs, similar to panel A. Names in italic font if imprinted gene was transcriptionally upregulated after *Setdb1* KO. **E**, Heatmap of DNAme levels at ICRs in WT and *Setdb1* KO serum ESCs. ICRs are organized by WT DNAme levels. **F**, Line graph illustrating median DNAme levels at ICRs (zero distance) and their immediate genomic surroundings.

Figure S5

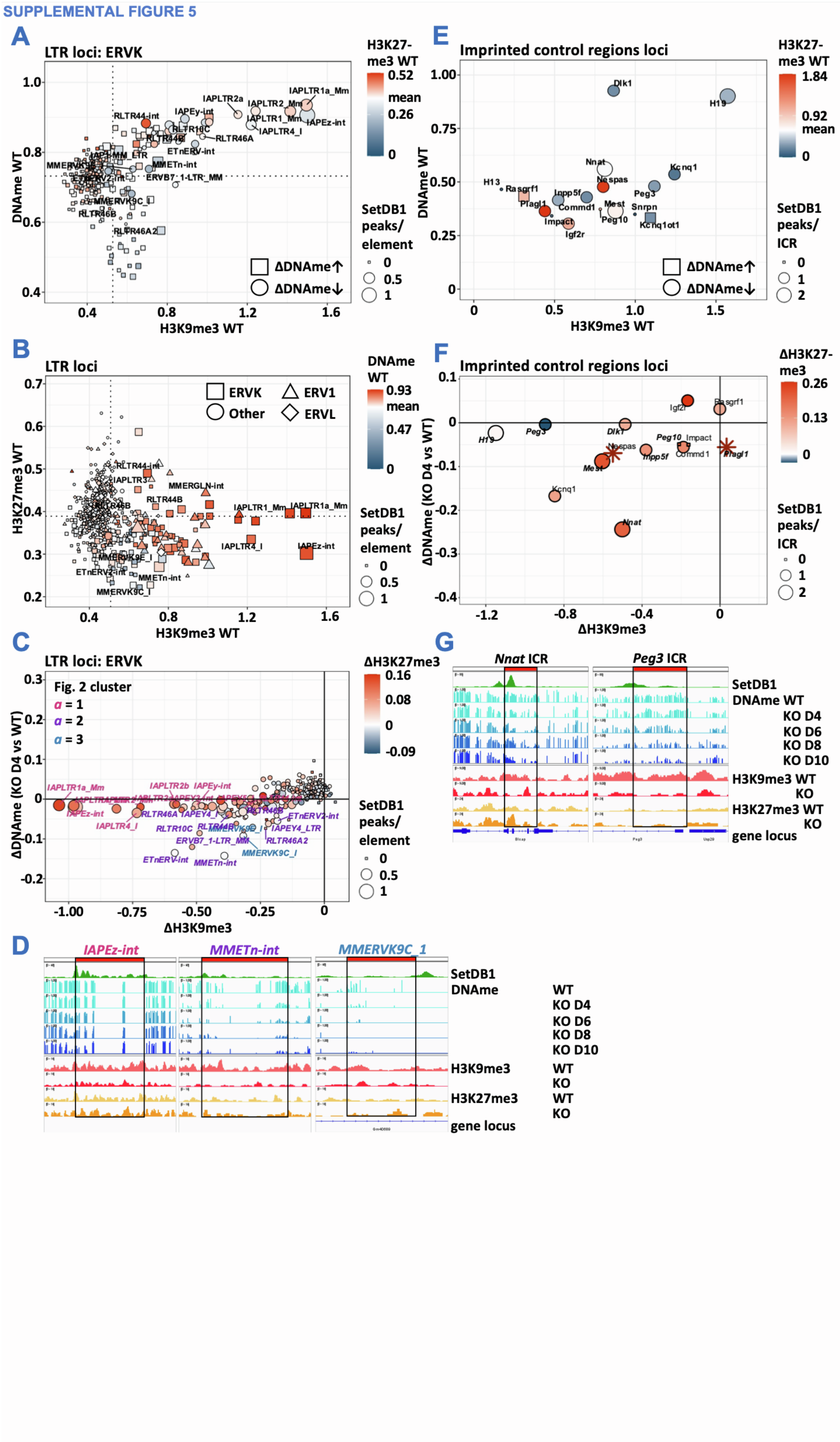

SUPPLEMENTAL FIGURE 5
A
LTR loci: ERVK
H3K9me3 WT
DNAme WT
H3K27-me3 WT
ΔDNAme↑
ΔDNAme↓
SetDB1 peaks/ element
B
LTR loci
ERVK
ERV1
Other
ERVL
H3K27me3 WT
DNAme WT
C
LTR loci: ERVK
Fig. 2 cluster
ΔH3K9me3
ΔDNAme (KO D4 vs WT)
ΔH3K27me3
D
IAPEz-int
MMETn-int
MMERVK9C_I
SetDB1
DNAme
WT
KO D4
KO D6
KO D8
KO D10
H3K9me3
H3K27me3
KO
gene locus
E
Imprinted control regions loci
SetDB1 peaks/ ICR
F
Imprinted control regions loci
ΔH3K27-me3
G
Nnat ICR
Peg3 ICR
DNAme WT
H3K9me3 WT
H3K27me3 WT

Figure S5: ERVK and ICR targets with SetDB1-dependent DNAme-H3K9me3 lose both repressive marks following *Setdb1* KO, related to Main Figure 2, and Supplemental Figure 4 & 6. **A**, Scatter plot of LTR-ERVKs showing WT H3K9me3 (RPKM), WT DNAme, and WT H3K27me3 (RPKM) levels at ERVKs in serum ESCs. Number of SetDB1 ChIP-seq peaks per ERVK copy (1kb proximity) displayed as point size. Dotted lines represent averages of their respectively axis parameters. **B**, Scatter plot of all LTRs, similar to panel A, but parameters of vertical axis and color scale swapped. **C**, Scatter plot similar to Supplemental Fig. 4A, but vertical axis changed to ΔDNAme KO day 4 vs WT. **D**, IGV screenshots of IAPEz-int (Chr11:120,212,299-120,218,751), MMETn-int (Chr10:83,236,248-83,240,631) and MMERVK9C_1 (Chr10:70,673,637-70,675,369) loci showing WGBS and ChIP-seq signals and overlap with genic loci. Color of name tags transferred from panel C. **E**, Scatter plot of ICRs, similar to panel A. **F** Scatter plot of ICRs, similar to Supplemental Fig. 4A. Names in italic font if imprinted gene was transcriptionally upregulated after *Setdb1* KO. **G**, IGV screenshots of *Nnat* and *Peg3* ICR, similar to panel D.

Figure S6

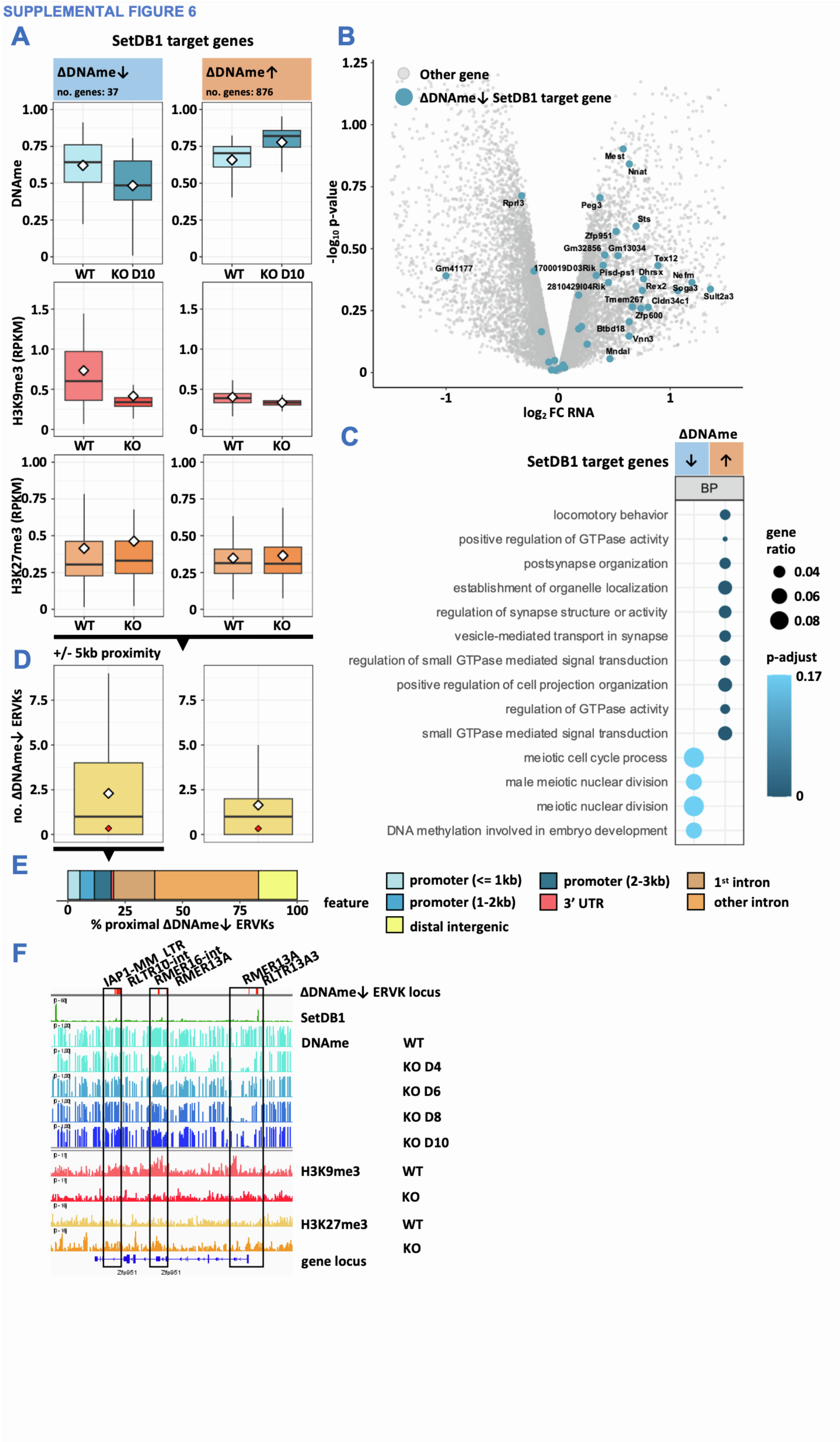

SUPPLEMENTAL FIGURE 6
A
SetDB1 target genes
ΔDNAme↓
no. genes: 37
ΔDNAme↑
no. genes: 876
DNAme
WT
KO D10
H3K9me3 (RPKM)
KO
H3K27me3 (RPKM)
+/- 5kb proximity
B
Other gene
ΔDNAme↓ SetDB1 target gene
-log10 p-value
log2 FC RNA
Mest
Nnat
Rprl3
Peg3
Sts
Zfp951
Gm32856
Gm13034
Gm41177
1700019D03Rik
Tex12
Plsd-ps1
Dhrsx
Nefm
2810429I04Rik
Rex2
Soga3
Sult2a3
Tmem267
Cldn34c1
Zfp600
Btbd18
Vnn3
Mndal
C
ΔDNAme
SetDB1 target genes
BP
locomotory behavior
positive regulation of GTPase activity
postsynapse organization
establishment of organelle localization
regulation of synapse structure or activity
vesicle-mediated transport in synapse
regulation of small GTPase mediated signal transduction
positive regulation of cell projection organization
regulation of GTPase activity
small GTPase mediated signal transduction
meiotic cell cycle process
male meiotic nuclear division
meiotic nuclear division
DNA methylation involved in embryo development
gene ratio
0.04
0.06
0.08
p-adjust
0.17
0
D
no. ΔDNAme↓ ERVKs
E
% proximal ΔDNAme↓ ERVKs
feature
promoter (<= 1kb)
promoter (1-2kb)
distal intergenic
promoter (2-3kb)
3' UTR
1st intron
other intron
F
IAP1-MM_LTR
RLTR10-int
RMER16-int
RMER13A
RMER13A
RLTR13A3
ΔDNAme↓ ERVK locus
SetDB1
DNAme
WT
KO D4
KO D6
KO D8
KO D10
H3K9me3
WT
KO
H3K27me3
WT
KO
gene locus

Figure S6: **Genes with SetDB1-dependent DNAme-H3K9me3 are derepressed in serum ESCs following SetDB1 loss, related to Main Figure 2, and Supplemental Figure 4-5. A**, Boxplots of DNAme, H3K9me3, and H3K27me3 levels of SetDB1-targeted genes in WT and *Setdb1* KO serum ESCs, grouped by methylation change at day 10 of *Setdb1* KO. White diamonds represent averages. **B**, Volcano plot of differentially expressed genes in *Setdb1* KO serum ESCs compared to WT serum ESCs, as observed in RNA-seq data. Demethylated SetDB1-target genes are highlighted in blue. **C**, Dot plot showing biological processes enriched among SetDB1-targeted genes (demethylated or increasingly methylated by day 10 of *Setdb1* KO), as determined by GO-analysis. **D**, Boxplots showing number of demethylated ERVK copies within 5kb distance of SetDB1-target genes, as categorized in panel A. White diamonds represent observed averages. Red diamonds represent expected number of demethylated ERVKs near any gene. **E**, Barplot showing genomic positions of demethylated ERVK elements near demethylated SetDB1 target genes. Genomic annotations determined by ChIPseeker. **F**, IGV screenshots of the *Zfp951* locus, showing WGBS and ChIP-seq signals. Demethylated ERVK (by day 10 of *Setdb1* KO) loci indicated in red above IGV tracks.

Figure S7

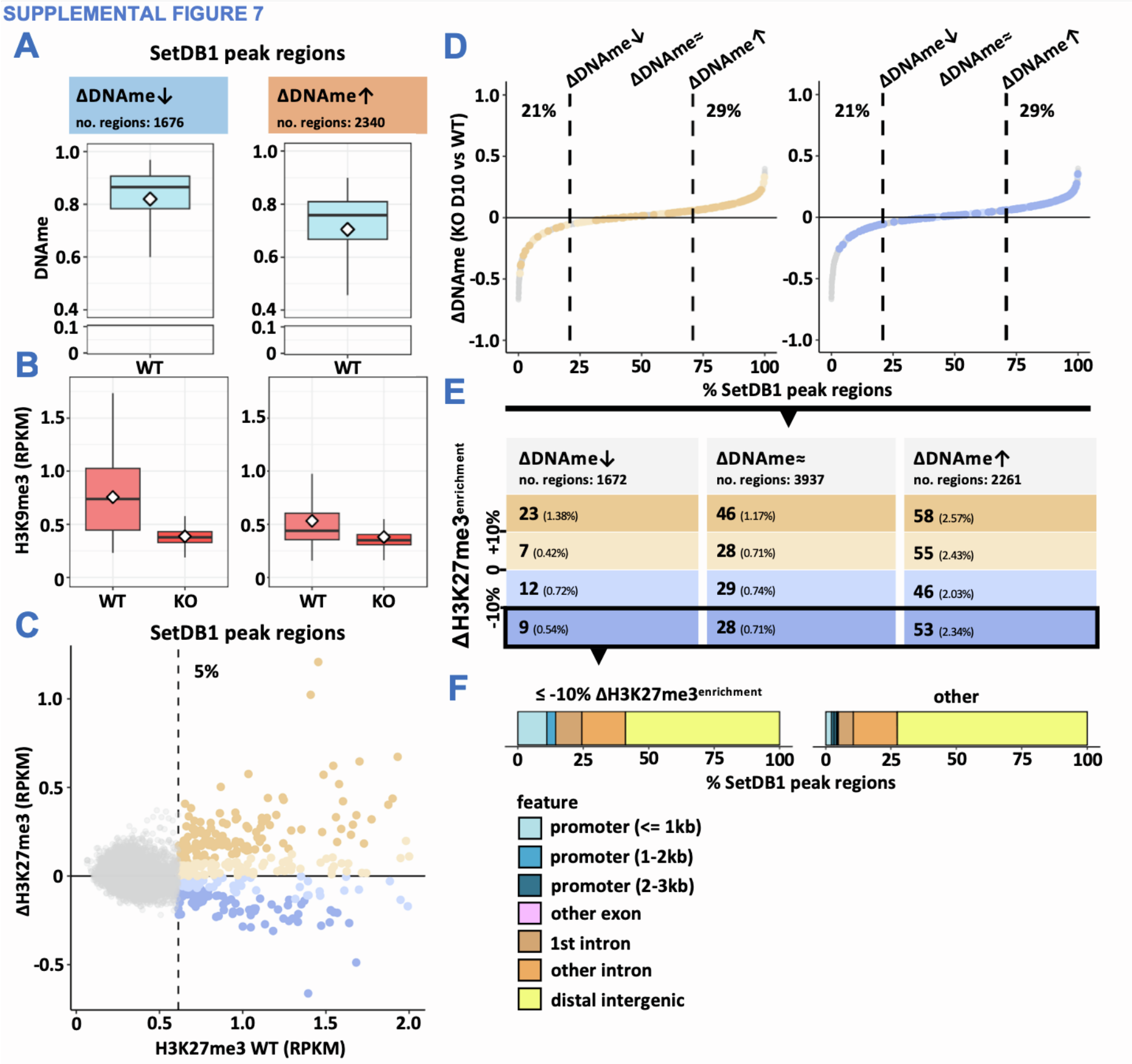

SUPPLEMENTAL FIGURE 7
A
SetDB1 peak regions
ΔDNAme↓
no. regions: 1676
ΔDNAme↑
no. regions: 2340
DNAme
WT
B
H3K9me3 (RPKM)
WT
KO
C
SetDB1 peak regions
5%
ΔH3K27me3 (RPKM)
H3K27me3 WT (RPKM)
D
ΔDNAme↓
ΔDNAme≈
ΔDNAme↑
21%
29%
ΔDNAme (KO D10 vs WT)
% SetDB1 peak regions
E
ΔDNAme↓
no. regions: 1672
ΔDNAme≈
no. regions: 3937
ΔDNAme↑
no. regions: 2261
ΔH3K27me3enrichment
23 (1.38%)
46 (1.17%)
58 (2.57%)
7 (0.42%)
28 (0.71%)
55 (2.43%)
12 (0.72%)
29 (0.74%)
46 (2.03%)
9 (0.54%)
28 (0.71%)
53 (2.34%)
F
≤ -10% ΔH3K27me3enrichment
other
% SetDB1 peak regions
feature
promoter (<= 1kb)
promoter (1-2kb)
promoter (2-3kb)
other exon
1st intron
other intron
distal intergenic

Figure S7: SetDB1-mediated H3K27me3-regulation is uncoupled from its DNAme-H3K9me3 axis, related to Main Figure 3. A, Boxplots of WT DNAme levels in serum ESCs within SetDB1 peak regions as defined in Fig. 3A (10kb centered on ChIP-seq peak summits). White diamonds represent averages. **B**, Boxplots of H3K9me3 levels in WT and *Setdb1* KO serum ESCs within SetDB1 peak regions as defined in Fig. 3A. White diamonds represent observed averages. **C**, Scatterplot of SetDB1 peak regions showing H3K27me3 in WT and *Setdb1* KO serum ESCs. Peak regions were further filtered relative to panels A and B to exclude regions with excessive H3K27me3 by removing the top 2% of WT H3K27me3 regions. Dashed vertical line represents 95th quantile of filtered regions. Colors match H3K27me3 remodeling category: orange, > 10% H3K27me3 gain; light orange, 0-10% gain; light blue, 0-10% loss; blue, >10% loss. **D**, Remapping of H3K27me3 remodeling categories from panel C to ΔDNAme-based ordering of SetDB1 peak regions of Fig. 3A. **E**, Table showing number of H3K27me3-enriched SetDB1 peak region categories from panel C within ΔDNAme-based categories of SetDB1 peak regions as shown in Fig. 3A. **F**, Barplot showing genomic positions of SetDB1 peaks, highlighting a subset of peaks whose regions lose H3K27me3 enrichment after *Setdb1* KO. Genomic annotations determined by ChIPseeker.

Figure S8

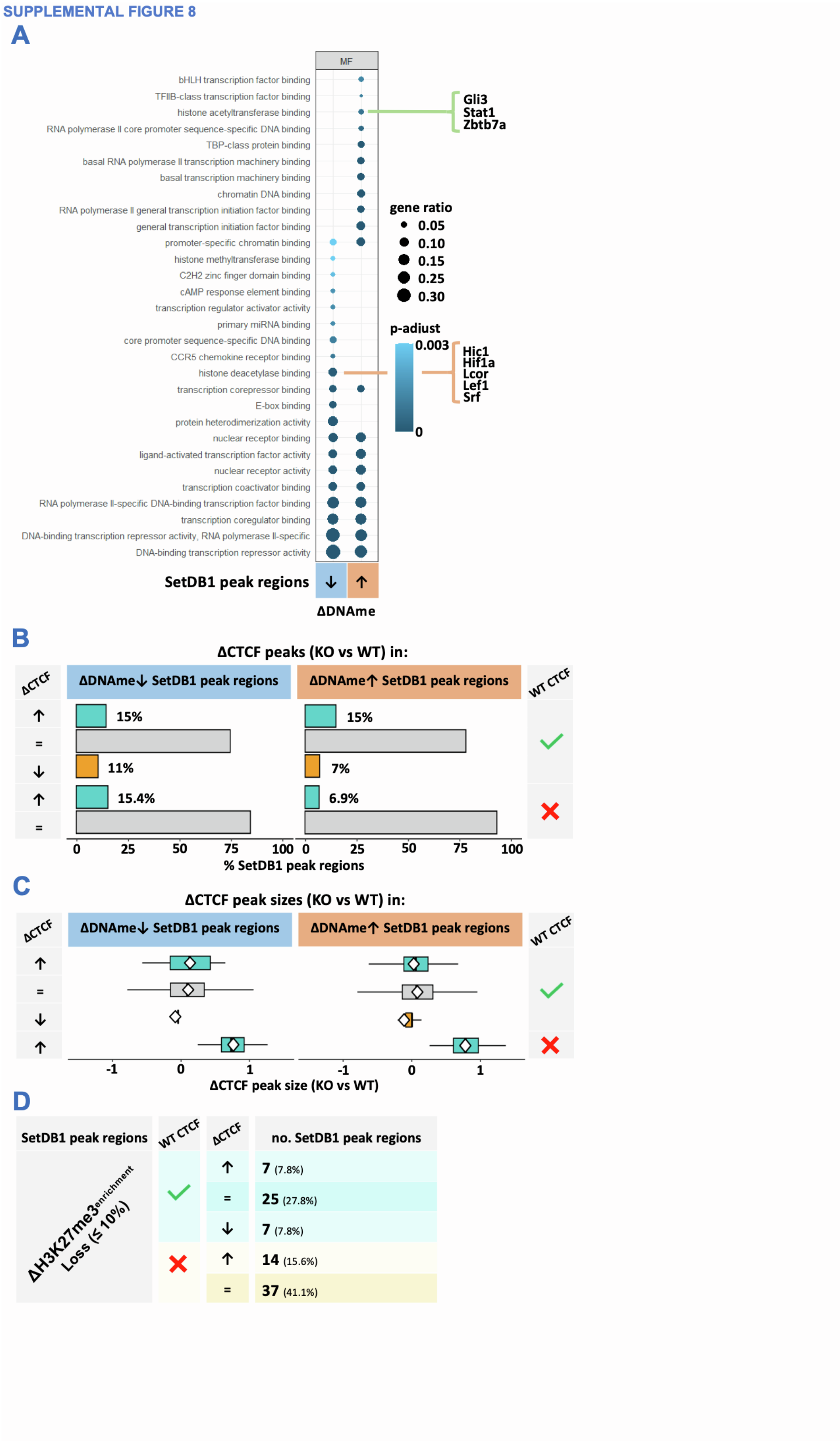

SUPPLEMENTAL FIGURE 8
A
MF
bHLH transcription factor binding
TFIIB-class transcription factor binding
histone acetyltransferase binding
RNA polymerase II core promoter sequence-specific DNA binding
TBP-class protein binding
basal RNA polymerase II transcription machinery binding
basal transcription machinery binding
chromatin DNA binding
RNA polymerase II general transcription initiation factor binding
general transcription initiation factor binding
promoter-specific chromatin binding
histone methyltransferase binding
C2H2 zinc finger domain binding
cAMP response element binding
transcription regulator activator activity
primary miRNA binding
core promoter sequence-specific DNA binding
CCR5 chemokine receptor binding
histone deacetylase binding
transcription corepressor binding
E-box binding
protein heterodimerization activity
nuclear receptor binding
ligand-activated transcription factor activity
nuclear receptor activity
transcription coactivator binding
RNA polymerase II-specific DNA-binding transcription factor binding
transcription coregulator binding
DNA-binding transcription repressor activity, RNA polymerase II-specific
DNA-binding transcription repressor activity
Gli3
Stat1
Zbtb7a
gene ratio
0.05
0.10
0.15
0.25
0.30
p-adjust
0.003
0
Hic1
Hif1a
Lcor
Lef1
Srf
SetDB1 peak regions ↓ ↑
ΔDNAme
B
ΔCTCF peaks (KO vs WT) in:
ΔCTCF
ΔDNAme↓ SetDB1 peak regions
ΔDNAme↑ SetDB1 peak regions
WT CTCF
15%
11%
15.4%
15%
7%
6.9%
0 25 50 75 100
% SetDB1 peak regions
C
ΔCTCF peak sizes (KO vs WT) in:
-1 0 1
ΔCTCF peak size (KO vs WT)
D
SetDB1 peak regions
WT CTCF
ΔCTCF
no. SetDB1 peak regions
ΔH3K27me3 enrichment Loss (≤ 10%)
7 (7.8%)
25 (27.8%)
7 (7.8%)
14 (15.6%)
37 (41.1%)

Figure S8: SetDB1-mediated CTCF-regulation is uncoupled from its DNAme-H3K9me3 and H3K27me3 axes, related to Main Figure 3. A, Dot plot showing molecular functions of TFs whose motifs are enriched in SetDB1 peak region categories from Fig. 3A, as determined by GO-analysis. **B**, Bar plots of SetDB1 peak regions, as defined in Fig. 3A and further grouped by the presence of CTCF WT peaks, showing the percentage of peak regions with changing (gain, loss) or equal (same) number of CTCF peaks upon *Setdb1* KO in serum ESCs. **C**, Boxplots showing change in average CTCF peak size between WT and *Setdb1* KO serum ESCs of categories denoted in panel B. White diamonds represent observed averages. **D**, Table summarizing CTCF peak remodeling at H3K27me3-mediated SetDB1 peak regions, as defined in Supplemental Fig. 7. Numbers reflect number and percentage of H3K27me3-mediated SetDB1 peak regions within categories.

Figure S9

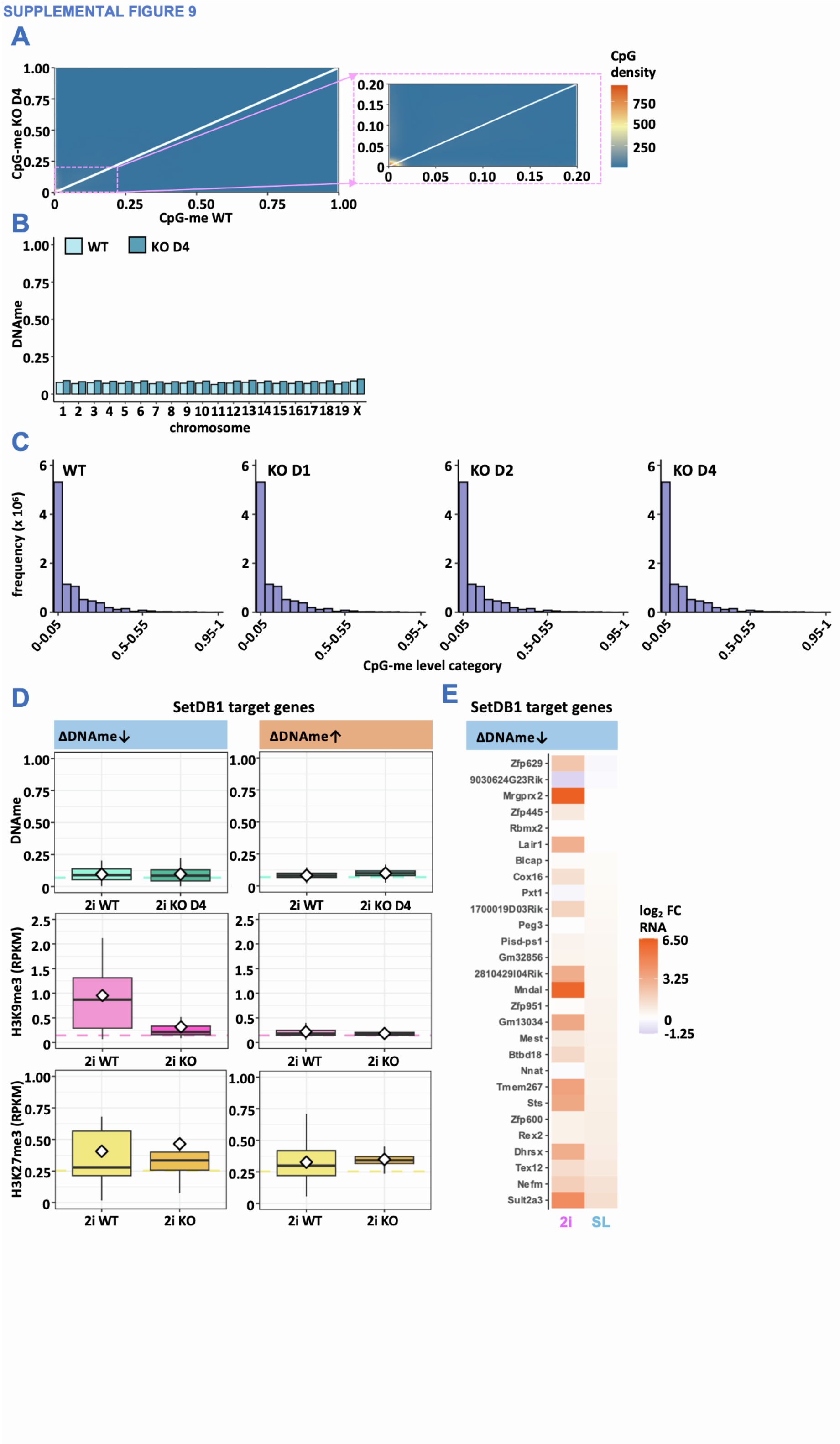

SUPPLEMENTAL FIGURE 9
A
CpG-me KO D4
CpG-me WT
CpG density
750
500
250
B
WT
KO D4
DNAme
chromosome
C
WT
KO D1
KO D2
KO D4
frequency (x 10⁶)
CpG-me level category
D
SetDB1 target genes
ΔDNAme↓
ΔDNAme↑
DNAme
2i WT
2i KO D4
H3K9me3 (RPKM)
2i WT
2i KO
H3K27me3 (RPKM)
E
SetDB1 target genes
ΔDNAme↓
Zfp629
9030624G23Rik
Mrgprx2
Zfp445
Rbmx2
Lair1
Blcap
Cox16
Pxt1
1700019D03Rik
Peg3
Pisd-ps1
Gm32856
2810429I04Rik
Mndal
Zfp951
Gm13034
Mest
Btbd18
Nnat
Tmem267
Sts
Zfp600
Rex2
Dhrsx
Tex12
Nefm
Sult2a3
2i
SL
log₂ FC RNA
6.50
3.25
0
-1.25

Figure S9: Hypomethylated 2i ESCs rely on SetDB1-mediated H3K9me3 to silence ERVK-proximal genes, related to Main Figure 4, and Supplemental Figure 1-3. A, Heatmaps showing methylation remodeling of individual CpGs upon *Setdb1* KO, relative to WT in 2i ESCs. CpG count density on color scale. Diagonal white lines represent slope = 1. **B**, Bar graph showing average methylation of chromosomes in WT and *Setdb1* KO in 2i ESCs (WT and day 4). **C**, Bar graph of CpG methylation levels in WT and *Setdb1* KO 2i ESCs, similar to Supplemental Fig. 2A. **D**, Boxplots of DNAme, H3K9me3, and H3K27me3 levels in WT and KO 2i ESCs at SetDB1 target genes from serum ESCs (Supplemental Fig. 6A: ΔDNAme↓ category). Dotted lines represent median WT levels in 2i ESCs. White diamonds represent averages. **E**, Heat map showing expression difference (KO vs WT) of SetDB1 target genes defined in panel D. Expression differences are denoted as $\log_2$ fold-change, and shown for ESCs cultured in serum and 2i.

Figure S10

SUPPLEMENTAL FIGURE 10

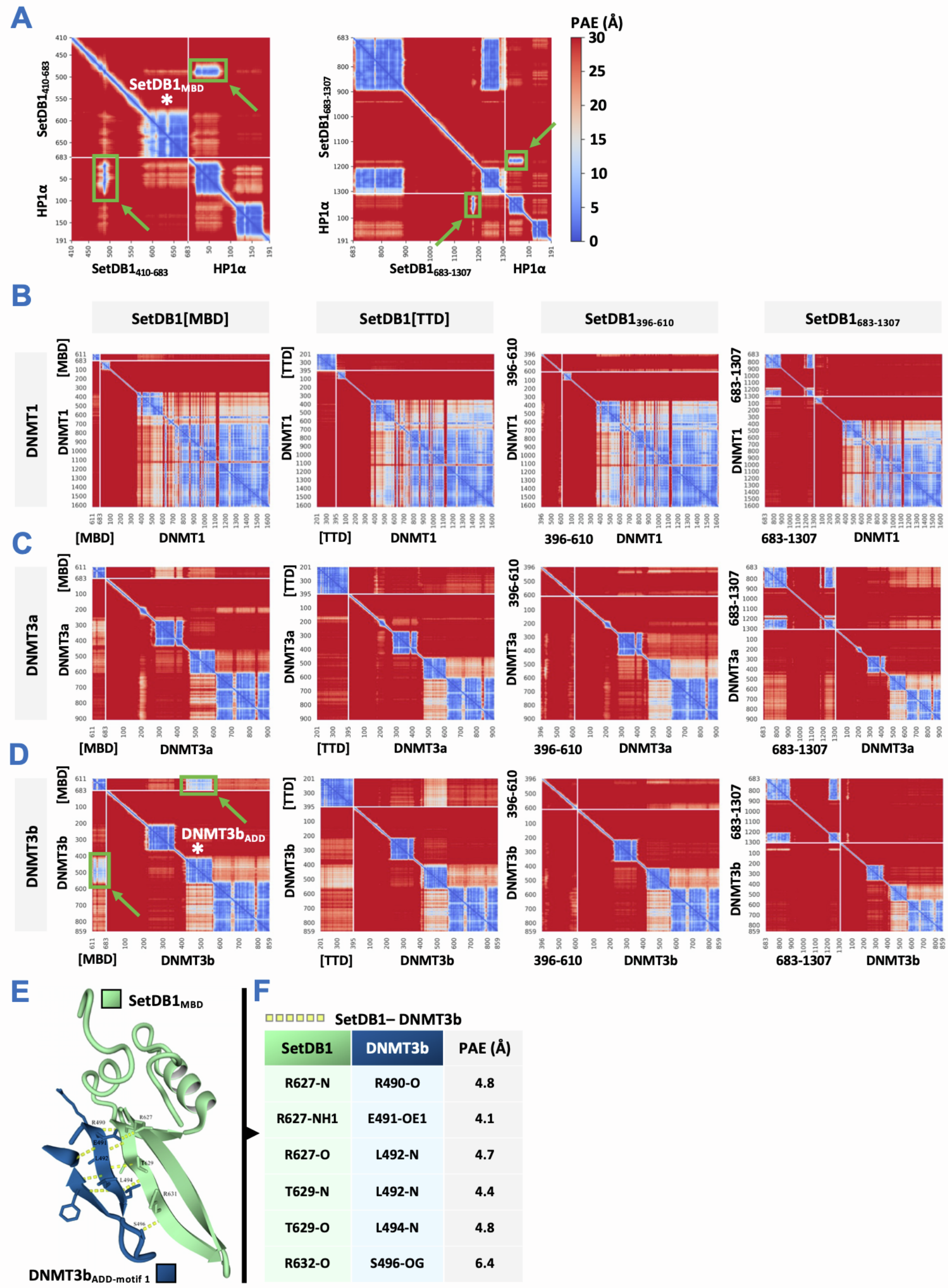

SetDB1	DNMT3b	PAE (Å)
R627-N	R490-O	4.8
R627-NH1	E491-OE1	4.1
R627-O	L492-N	4.7
T629-N	L492-N	4.4
T629-O	L494-N	4.8
R632-O	S496-OG	6.4

Figure S10: Modeling of interactions predicted by AlphaFold3 between domains of SetDB1 and full-length DNMT1, -3a, and -3b, related to Main Figure 5, and Supplemental Figure 11-12. A, PAE heatmaps, similar to Fig. 5A, between SetDB1 residues 410-683 and 683-1307 and full-length HP1α, as positive control. All amino acid sequences from mouse. **B-D**, PAE heatmaps, similar to Fig. 5A, between the MBD and TTD of SetDB1, as well as SetDB1 flexible regions spanning amino acids 396-610 (Flex 1) and 683-1307 (Flex 2), and full-length DNMT1, -3a, and -3b. All amino acid sequences from mouse. **E**, Close-up view of structure of interacting residues from Fig. 5B. Yellow-dotted lines indicate hydrogen bonds, and interacting residues are labeled. **F**, Summary of residues involved in SetDB1-DNMT3b interaction as modeled in panel E, including residue number, interacting atoms, and PAE values in ängstrom.

Figure S11

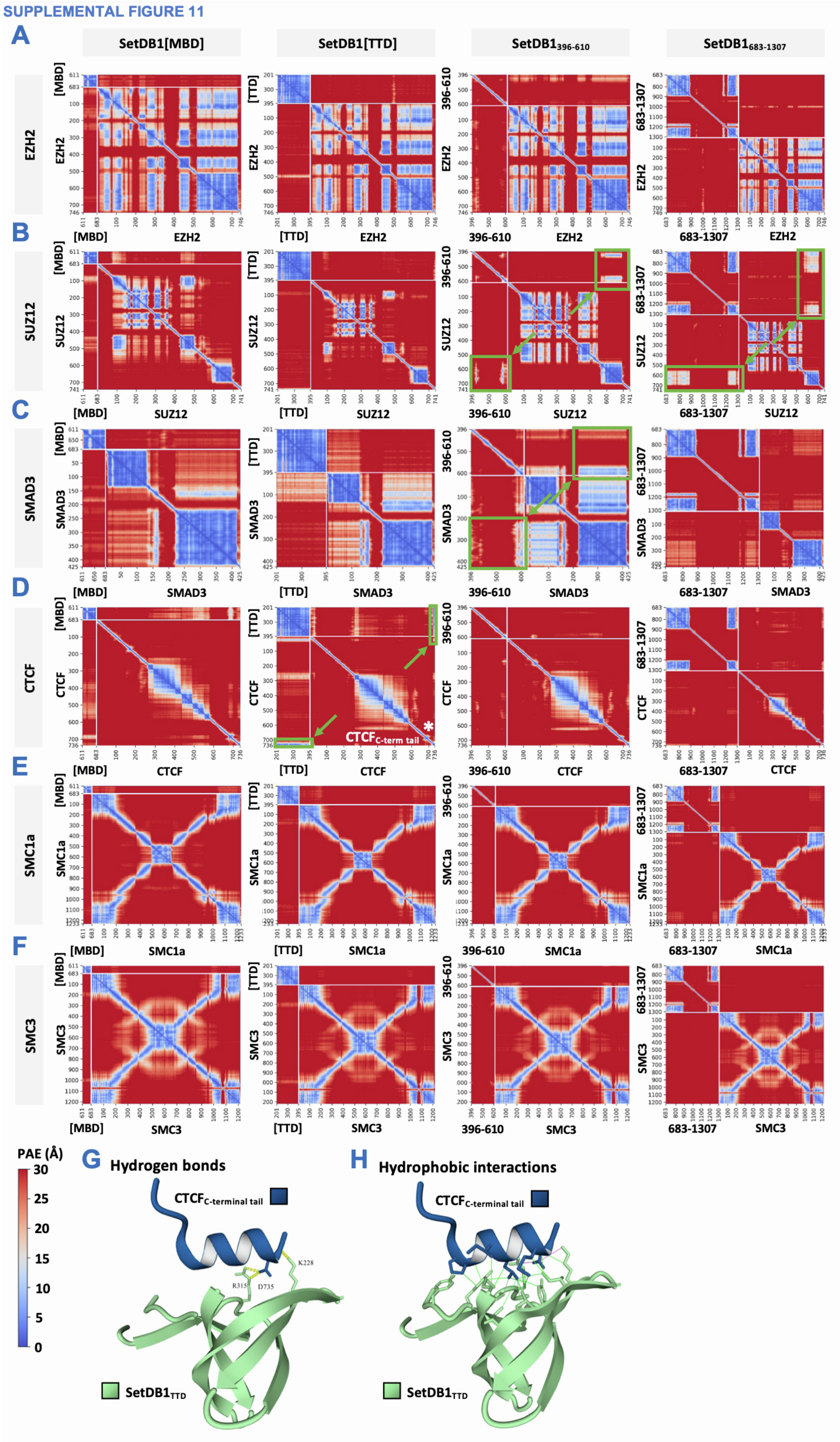
SUPPLEMENTAL FIGURE 11
A
SetDB1[MBD]
SetDB1[TTD]
SetDB1396-610
SetDB1683-1307
EZH2
B
SUZ12
C
SMAD3
D
CTCF
CTCFC-term tail
E
SMC1a
F
SMC3
PAE (Å)
30
25
20
15
10
5
0
G
Hydrogen bonds
CTCFC-terminal tail
K228
R315
D735
SetDB1TTD
H
Hydrophobic interactions
CTCFC-terminal tail
SetDB1TTD

Figure S11: Modeling of interactions predicted by AlphaFold3 between domains of SetDB1 and full-length EZH2, SUZ12, SMAD3, CTCF, and SMC1a and -3, related to Main Figure 5, and Supplemental Figure 10 & 12. A-F, PAE heatmaps, similar to Fig. 5A, between the MBD and TTD of SetDB1, as well as SetDB1 flexible regions spanning amino acids 396-610 (Flex 1) and 683-1307 (Flex 2), and full-length EZH2, SUZ12, SMAD3, CTCF, SMC1a, and SMC3. All amino acid sequences from mouse. **G-H**, Close-up view of structure of interacting residues from panel D. Yellow-dotted lines in panel G indicate hydrogen bonds, and interacting residues are labeled. Thin lines in panel H indicate hydrophobic interactions.

Figure S12

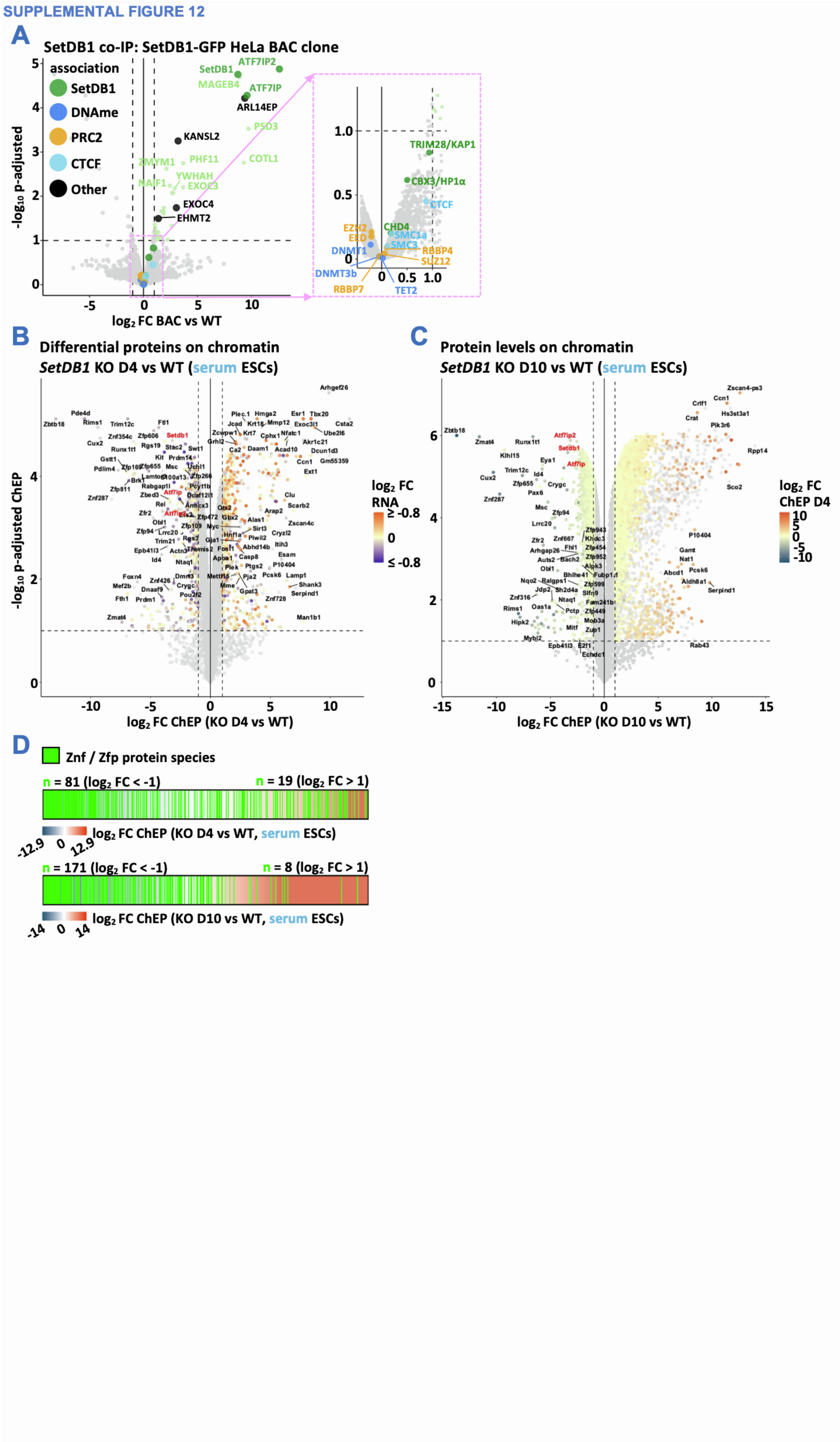
SUPPLEMENTAL FIGURE 12
A
SetDB1 co-IP: SetDB1-GFP HeLa BAC clone
association
SetDB1
DNAme
PRC2
CTCF
Other
-log₁₀ p-adjusted
log₂ FC BAC vs WT
B
Differential proteins on chromatin
SetDB1 KO D4 vs WT (serum ESCs)
-log₁₀ p-adjusted ChEP
log₂ FC ChEP (KO D4 vs WT)
log₂ FC RNA
C
Protein levels on chromatin
SetDB1 KO D10 vs WT (serum ESCs)
log₂ FC ChEP (KO D10 vs WT)
log₂ FC ChEP D4
D
Znf / Zfp protein species
n = 81 (log₂ FC < -1)
n = 19 (log₂ FC > 1)
log₂ FC ChEP (KO D4 vs WT, serum ESCs)
n = 171 (log₂ FC < -1)
n = 8 (log₂ FC > 1)
log₂ FC ChEP (KO D10 vs WT, serum ESCs)

Figure S12: Proteomics of SetDB1 reveal its interactome, as well as chromatome remodeling following SetDB1 loss, related to Main Figure 5, and Supplemental Figure 10-11. A, Volcano plot of proteins associated with SetDB1, as measured by co-IP MS on nuclear extracts from SetDB1-GFP BAC clone HeLa cells compared to WT HeLa cells. Enrichments are denoted as $\log_2$ fold-change in BAC vs WT extracts, accompanied by $-\log_{10}$ adjusted p-values (vertical axis). Thresholds for enrichment ($\log_2$ fold-change ≥ 1) and significance ($-\log_{10}$ p-adjusted ≥ 1) indicated as dashed lines. **B**, Volcano plot of differential proteins on chromatin after 4 days of *Setdb1* KO in serum ESCs, compared to WT serum ESCs, as measured by ChEP-MS. Significance and differentiality thresholds are indicated by dashed lines corresponding to a $-\log_{10}$ p-adjusted of 1, and $\log_2$ fold-change cutoffs of -1 and 1, respectively. Color of data points are indicated if proteins were significantly differential, and represents their $\log_2$ fold-change as observed in RNA-seq data (at day 5 of *Setdb1* KO induction: Barral et al. 2017). **C**, Volcano plot similar to panel A, but $\log_2$ fold-changes represent difference at 10 days of *Setdb1* KO in serum ESCs, compared to WT. Color scale represents $\log_2$ fold-change difference at 4 days of *Setdb1* KO. Grey dot above thresholds indicates protein is recruited/lost to chromatin at day 10, but not yet at day 4 post-KO induction. **D**, Heatmaps showing $\log_2$ fold-changes of chromatin-bound proteins at day 4 and 10 after *Setdb1* KO induction in serum ESCs, as measured by ChEP-MS. Proteins in heatmaps are ordered by their $\log_2$ fold-change, from low to high. If protein is encoded by *Znf* or *Zfp* gene, it is indicated in green.

Supplemental notes

Supplemental Note 1: SetDB1 loss triggers DNAme-H3K9me3 erasure and reactivation of targeted ERVKs and nearby genes, and ICRs, related to Main Figure 1-2, and Figure S3-S6.

Our binning approach applied to the methylome data collected from SetDB1-deficient serum ESCs does not easily allow investigation of transcriptional responses from individual genomic elements. To facilitate such analysis, we also projected the data on the genomic loci directly. Consistent with results at 10kb binning scale described in Figures 1 and 2, various ERVK loci (11-7710bp, median size: 440bp) are targeted by SetDB1 and enriched with DNAme and H3K9me3 (Supplemental Fig. 5A). Among these, IAPEz elements show the greatest SetDB1 targeting and enrichment of DNAme and H3K9me3. A minority of LTR regions of IAPs and other ERVKs are also marked with H3K27me3. A similar co-occurrence of H3K9me3 and H3K27me3 was observed in PGCs, including at hypermethylated IAPs (Liu et al., 2014). However, H3K27me3 coverage is generally greatest on ERVKs and other LTR subfamilies that lack H3K9me3 (and DNAme) enrichment in serum ESCs (Supplemental Fig. 5B). This aligns with previous findings that H3K9me3 and H3K27me3 mostly mark distinct genomic regions in ESCs (Fei et al., 2015), and supports the hypothesis that H3K27me3 may repress retrotransposons that are not marked by SetDB1-dependent H3K9me3 and DNAme (Prokopuk et al., 2017). Notably, H3K27me3-enriched LTRs are rarely bound by SetDB1 compared to DNAme-H3K9me3-enriched LTRs (Supplemental Fig. 5A), suggesting that SetDB1 preferentially regulates its retrotransposon targets via H3K9me3 in combination with DNAme. Furthermore, consistent with Figures 1 and 2, strongly targeted ERVKs were demethylated and completely lost their H3K9me3 enrichment after *Setdb1* KO (Supplemental Fig. 4A; Supplemental Fig. 5C-D). Following the loss of DNAme and H3K9me3 on ERVKs, we noticed a minor increase in H3K27me3 (Supplemental Fig. 4A), which is in line with our previous observation that loss of DNAme allows PRC2 to deposit diffuse H3K27me3 (Van Mierlo et al., 2019; Walter et al., 2016).

To assay whether the chromatin remodeling at ERVKs following *Setdb1* KO results in their reactivation, we re-analyzed RNA-seq data generated from serum ESCs after *Setdb1* KO (Barral et al., 2022). Over 80 percent of ERVK elements show an increase in transcription after *Setdb1* KO (Supplemental Fig. 4B). SetDB1-targeted ERVKs that lost DNAme and H3K9me3 post-KO were upregulated by up to 3.3-fold. This indicates that the minor increase of H3K27me3 after SetDB1 depletion (Supplemental Fig. 4A) is insufficient to keep these retrotransposons repressed. Notably, upregulated ERVKs (> 1.5-fold increase of expression) include a large range of IAP, ETn, ERV-β2/4 and MERV elements (Supplemental Fig. 4C), all of which are capable of retrotransposition and pose a threat to genome integrity through insertional mutagenesis (Gagnier et al., 2019; Maksakova et al., 2006).

The presence of both DNAme and H3K9me3 is not exclusive to LTR-ERVKs, as this combination has also been observed at imprinted control regions (ICRs) (Fisher et al., 2017; Habibi et al., 2013; Leung et al., 2014), suggesting their potential dependency on SetDB1-mediated regulation. Indeed, 13 out of 17 (76%) ICRs described in the literature (Betto et al., 2021; Santini et al., 2021) are targeted by SetDB1, as indicated by 1-2 SetDB1 peaks within 1kb distance (Supplemental Fig. 4D). The DNAme level across the ICR averaged 47% and many ICRs were densely marked with H3K9me3 (Supplemental Fig. 5E). After *Setdb1* KO in serum ESCs, most ICRs lost H3K9me3 as well as proximal DNAme, the epigenetic mark that is essential for classical imprinting (Supplemental Fig. 4D-F; Supplemental Fig. 5F-G). Some ICRs were more susceptible to demethylation than others (Supplemental Fig. 4E), akin to specific ERVKs. Deposition of H3K27me3 slightly increased following *Setdb1* KO (except on the *Kcnq1ot1* and *Peg3* ICR), but this was insufficient to prevent the derepression of several imprinted genes (Supplemental Fig. 4D; italic names). Overall, these findings highlight a strong resemblance between SetDB1-mediated regulation of ICRs and ERVKs through a repressive DNAme-H3K9me3 axis in serum ESCs.

Our binning approach revealed that the SetDB1-targeted ΔDNAme↓ regions also include a small number of genic elements (Supplemental Fig. 3B). To examine which genes are regulated by the DNAme-mediated component of SetDB1, we investigated SetDB1-targeted genic loci. We observed only 37 demethylated target genes (ΔDNAme < -0.05) within 1kb proximity of at least one SetDB1 ChIP-seq peak. Similar to ERVKs and ICRs, demethylated target genes also lost their H3K9me3 enrichment after *Setdb1* KO (Supplemental Fig. 6A). Following *Setdb1* KO, 78 percent of demethylated target genes were increasingly expressed, up to 2.6 times (i.e. *Sult2a3*) (Supplemental Fig. 6B). GO-

enrichment analyses revealed enriched terms related to meiosis (Supplemental Fig. 6C), associated with *Btbd18*, *Rbm46* and *Tex12*, and consistent with previously reported silencing of meiosis- and gametogenesis-related genes by SetDB1 through DNAme-H3K9me3 (Karimi et al., 2011; Leung et al., 2014; Mochizuki et al., 2021; Wang et al., 2022). To find out whether nearby ERVKs are involved in the regulation of the demethylated target genes, we probed the number of demethylated ERVKs within 5kb proximity of these genes (Supplemental Fig. 6D). An enrichment for demethylated ERVKs was found near demethylated target genes, with more than two copies near demethylated SetDB1 target genes on average. Several of these ERVKs are embedded within regulatory elements of these genes, such as promoters, though the majority is found in introns (Supplemental Fig. 6E). For example, the derepressed *Zfp951* gene, that lost DNAme and H3K9me3 after *Setdb1* KO, embeds six demethylated ERVKs within or near its locus (Supplemental Fig. 6F). Hence, the DNAme-H3K9me3-mediated repression of most of these genes may originate from the targeting and regulation of flanking ERVKs by SetDB1. Notably, Karimi et al. (2011) reported that aberrant reactivation of ERVKs upon *Setdb1* KO is associated with disturbed expression of neighboring genes. Taken together, these observations suggest that ERVKs exert a DNAme- and H3K9me3-dependent repressive influence on nearby target genes, which is disrupted upon SetDB1 depletion.

Supplemental references

Barral, A., Pozo, G., Ducrot, L., Papadopoulos, G. L., Sauzet, S., Oldfield, A. J., ... & Déjardin, J. (2022). SETDB1/NSD-dependent H3K9me3/H3K36me3 dual heterochromatin maintains gene expression profiles by bookmarking poised enhancers. Molecular cell, 82(4), 816-832.

Betto, R. M., Diamante, L., Perrera, V., Audano, M., Rapelli, S., Lauria, A., ... & Martello, G. (2021). Metabolic control of DNA methylation in naive pluripotent cells. Nature genetics, 53(2), 215-229.

Fei, Q., Yang, X., Jiang, H., Wang, Q., Yu, Y., Yu, Y., ... & Shou, J. (2015). SETDB1 modulates PRC2 activity at developmental genes independently of H3K9 trimethylation in mouse ES cells. Genome research, 25(9), 1325-1335.

Fisher, C. L., Marks, H., Cho, L. T. Y., Andrews, R., Wormald, S., Carroll, T., ... & Skarnes, W. C. (2017). An efficient method for generation of bi-allelic null mutant mouse embryonic stem cells and its application for investigating epigenetic modifiers. Nucleic acids research, 45(21), e174-e174.

Gagnier, L., Belancio, V. P., & Mager, D. L. Mouse germ line mutations due to retrotransposon insertions. Mobile DNA. 2019; 10: 15. Epub 2019/04/24. doi: 10.1186/s13100-019-0157-4. PubMed PMID: 31011371.

Habibi, E., Brinkman, A. B., Arand, J., Kroeze, L. I., Kerstens, H. H., Matarese, F., ... & Stunnenberg, H. G. (2013). Whole-genome bisulfite sequencing of two distinct interconvertible DNA methylomes of mouse embryonic stem cells. Cell stem cell, 13(3), 360-369.

Karimi, M. M., Goyal, P., Maksakova, I. A., Bilenky, M., Leung, D., Tang, J. X., ... & Lorincz, M. C. (2011). DNA methylation and SETDB1/H3K9me3 regulate predominantly distinct sets of genes, retroelements, and chimeric transcripts in mESCs. Cell stem cell, 8(6), 676-687.

Leung, D., Du, T., Wagner, U., Xie, W., Lee, A. Y., Goyal, P., ... & Ren, B. (2014). Regulation of DNA methylation turnover at LTR retrotransposons and imprinted loci by the histone methyltransferase Setdb1. Proceedings of the National Academy of Sciences, 111(18), 6690-6695.

Liu, S., Brind'Amour, J., Karimi, M. M., Shirane, K., Bogutz, A., Lefebvre, L., ... & Lorincz, M. C. (2014). Setdb1 is required for germline development and silencing of H3K9me3-marked endogenous retroviruses in primordial germ cells. Genes & development, 28(18), 2041-2055.

Maksakova, I. A., Romanish, M. T., Gagnier, L., Dunn, C. A., Van de Lagemaat, L. N., & Mager, D. L. (2006). Retroviral elements and their hosts: insertional mutagenesis in the mouse germ line. PLoS genetics, 2(1), e2.

Mochizuki, K., Sharif, J., Shirane, K., Uranishi, K., Bogutz, A. B., Janssen, S. M., ... & Lorincz, M. C. (2021). Repression of germline genes by PRC1. 6 and SETDB1 in the early embryo precedes DNA methylation-mediated silencing. Nature communications, 12(1), 7020.

Prokopuk, L., Stringer, J. M., Hogg, K., Elgass, K. D., & Western, P. S. (2017). PRC2 is required for extensive reorganization of H3K27me3 during epigenetic reprogramming in mouse fetal germ cells. Epigenetics & chromatin, 10, 1-20.

Santini, L., Halbritter, F., Titz-Teixeira, F., Suzuki, T., Asami, M., Ma, X., ... & Leeb, M. (2021). Genomic imprinting in mouse blastocysts is predominantly associated with H3K27me3. Nature Communications, 12(1), 3804.

van Mierlo, G., Dirks, R. A., De Clerck, L., Brinkman, A. B., Huth, M., Kloet, S. L., ... & Marks, H. (2019). Integrative proteomic profiling reveals PRC2-dependent epigenetic crosstalk maintains ground-state pluripotency. Cell stem cell, 24(1), 123-137.

Walter, M., Teissandier, A., Pérez-Palacios, R., & Bourc'His, D. (2016). An epigenetic switch ensures transposon repression upon dynamic loss of DNA methylation in embryonic stem cells. elife, 5, e11418.

Wang, Z., Fan, R., Russo, A., Cernilogar, F. M., Nuber, A., Schirge, S., ... & Schotta, G. (2022). Dominant role of DNA methylation over H3K9me3 for IAP silencing in endoderm. Nature communications, 13(1), 5447.
